# Supplementary material for: Defining Lineage Potential and Fate Behavior of Precursors during Pancreas Development
Source: Dev Cell. 2018 Aug 6;46(3):360–375.e5. doi: 10.1016/j.devcel.2018.06.028 (PMC6085117; doi:10.1016/j.devcel.2018.06.028)
Supplement: Document S1. Figures S1–S7 and Methods S1, Related to STAR Methods [file mmc1.pdf]

Developmental Cell, Volume *46*

Supplemental Information

Defining Lineage Potential and Fate Behavior of Precursors during Pancreas Development

Magdalena K. Sznurkowska, Edouard Hannezo, Roberta Azzarelli, Steffen Rulands, Sonia Nestorowa, Christopher J. Hindley, Jennifer Nichols, Berthold Göttgens, Meritxell Huch, Anna Philpott, and Benjamin D. Simons

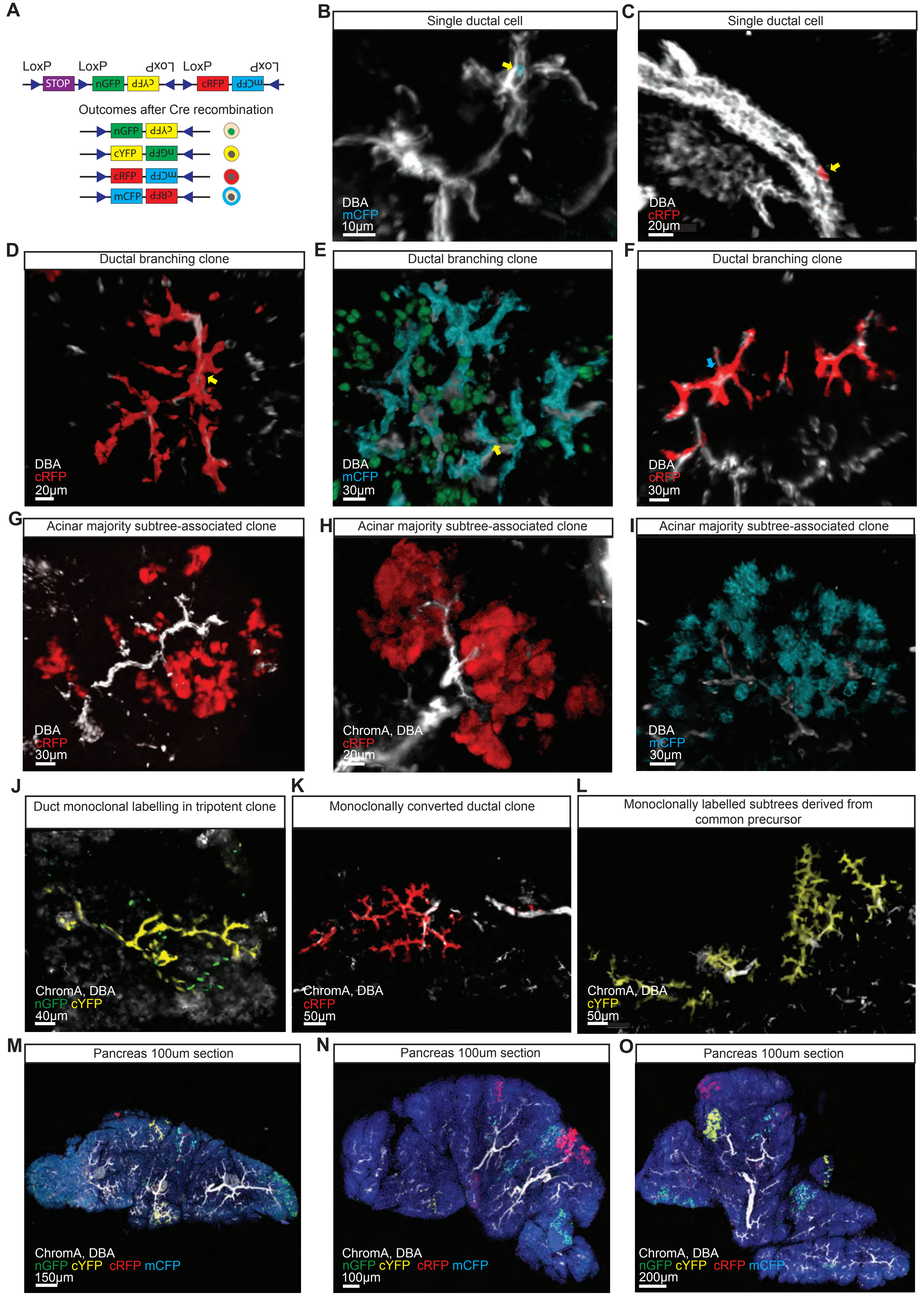

Supplemental Figure 1

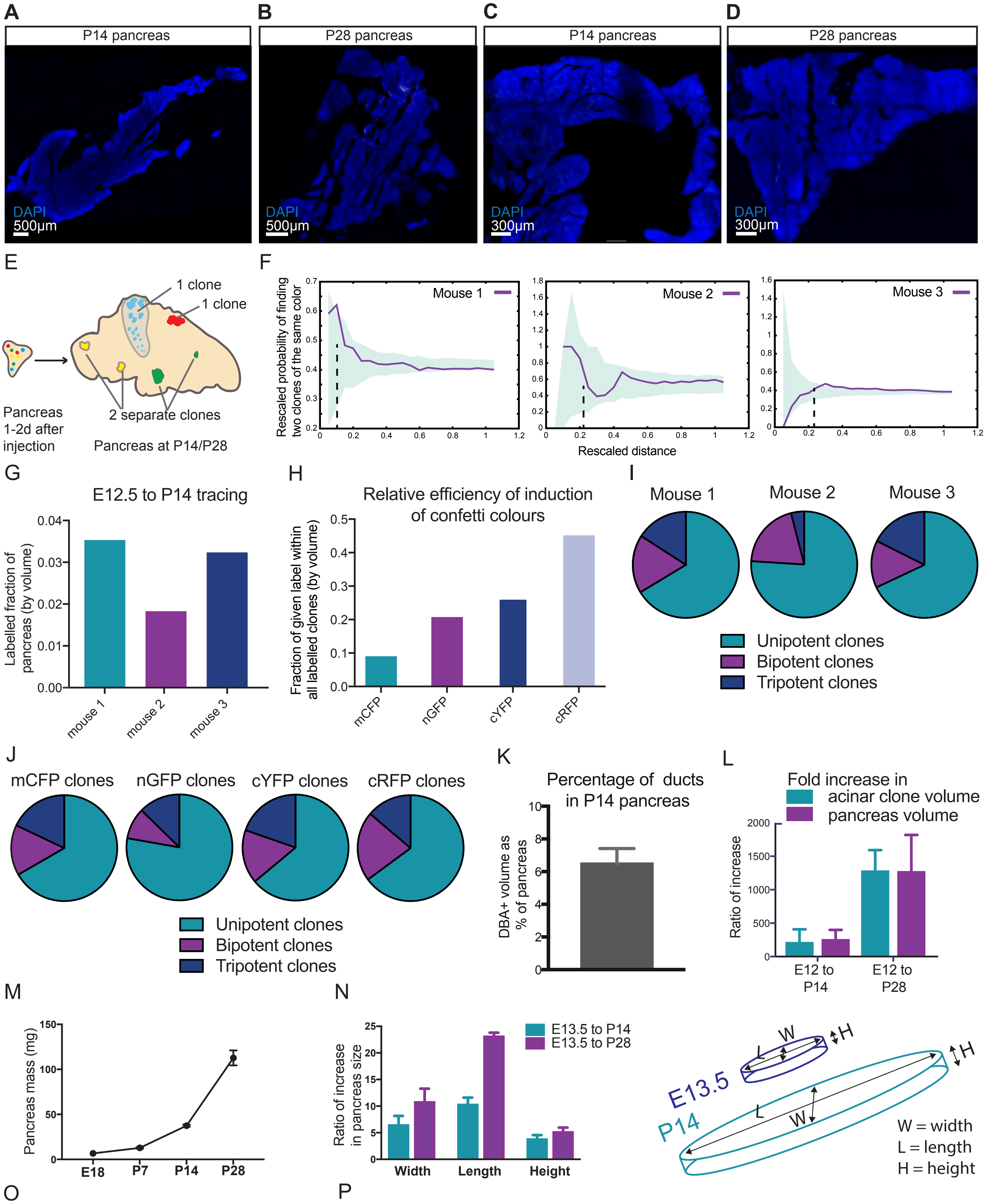

O

E12.5 to P14 - amylase control

Clone type	Frequency (%)	Number
Unipotent acinar	54.7	29
Unipotent ductal	7.5	4
Unipotent islet	3.8	2
Bipotent acinar-ductal	20.8	11
Bipotent acinar-islet	0.0	0
Bipotent ductal-islet	1.9	1
Tripotent	11.3	6

P

E12.5 to P14

Clone type	Frequency (%)	Number
Unipotent acinar	59.9	154
Unipotent ductal	3.1	8
Unipotent islet	5.1	13
Bipotent acinar-ductal	10.5	27
Bipotent acinar-islet	4.7	12
Bipotent ductal-islet	1.2	3
Tripotent	15.6	40

Supplemental Figure 2

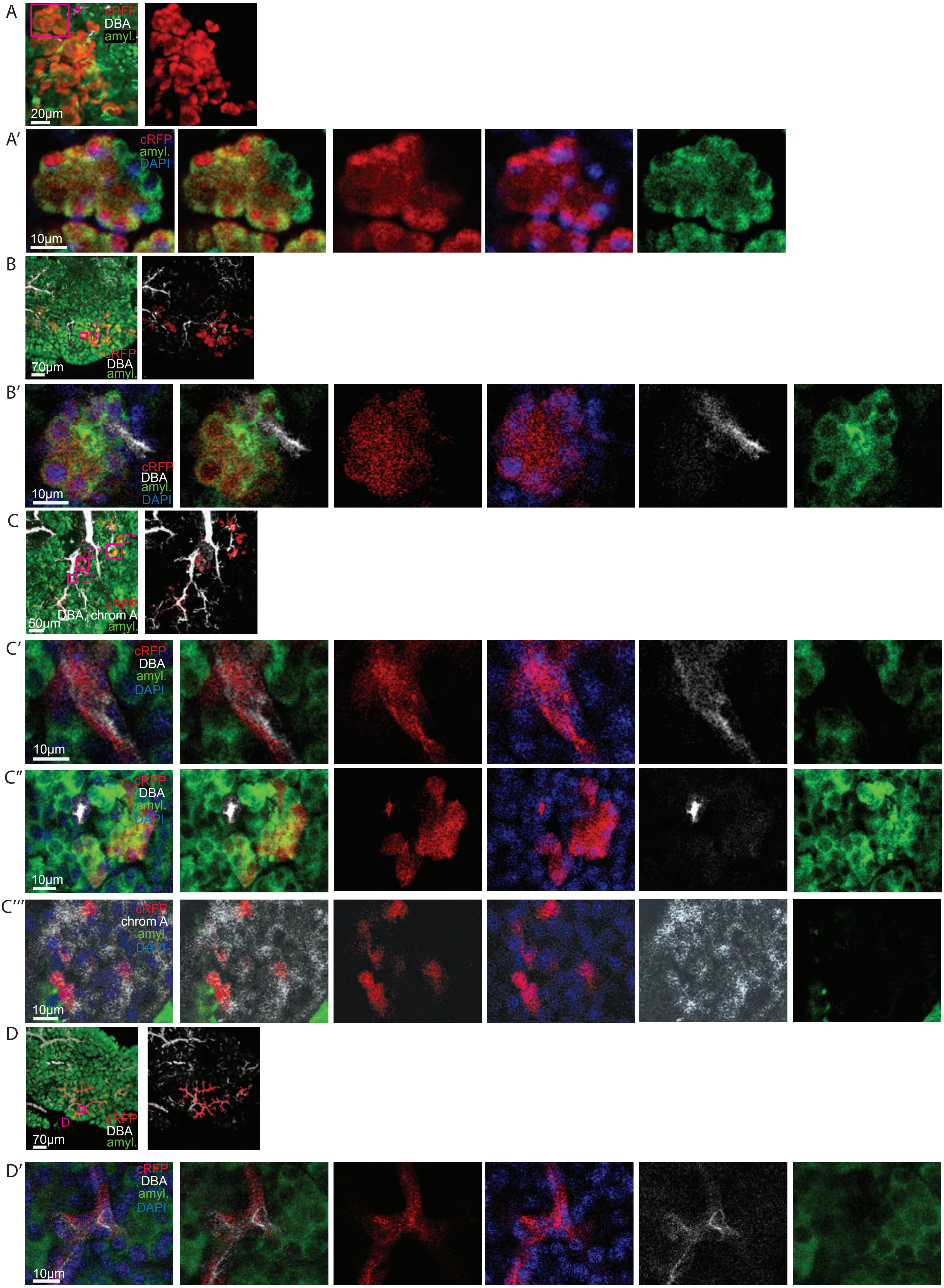

Supplemental Figure 3

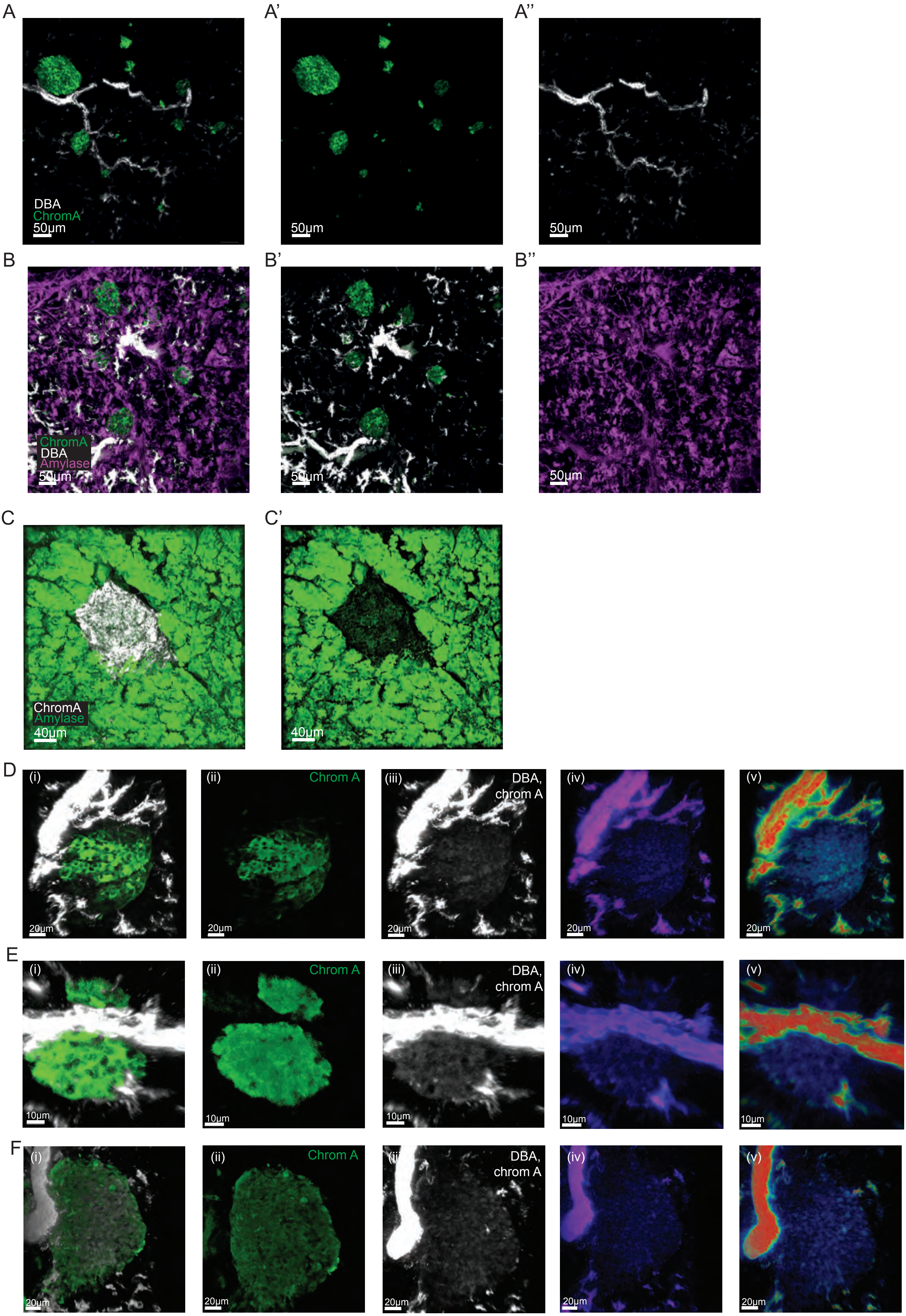

Supplemental Figure 4

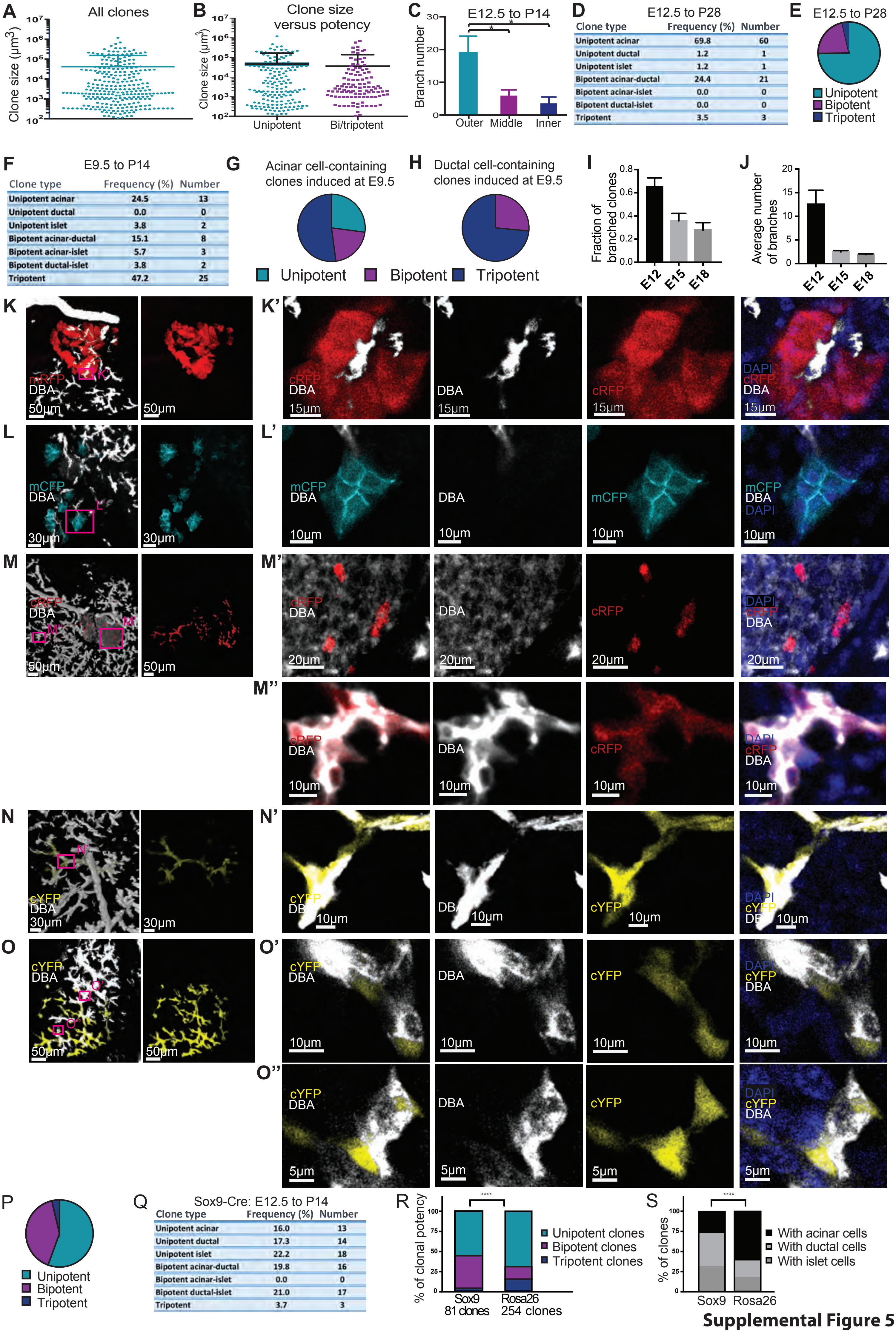

D — E12.5 to P28

Clone type	Frequency (%)	Number
Unipotent acinar	69.8	60
Unipotent ductal	1.2	1
Unipotent islet	1.2	1
Bipotent acinar-ductal	24.4	21
Bipotent acinar-islet	0.0	0
Bipotent ductal-islet	0.0	0
Tripotent	3.5	3

F — E9.5 to P14

Clone type	Frequency (%)	Number
Unipotent acinar	24.5	13
Unipotent ductal	0.0	0
Unipotent islet	3.8	2
Bipotent acinar-ductal	15.1	8
Bipotent acinar-islet	5.7	3
Bipotent ductal-islet	3.8	2
Tripotent	47.2	25

Q — Sox9-Cre: E12.5 to P14

Clone type	Frequency (%)	Number
Unipotent acinar	16.0	13
Unipotent ductal	17.3	14
Unipotent islet	22.2	18
Bipotent acinar-ductal	19.8	16
Bipotent acinar-islet	0.0	0
Bipotent ductal-islet	21.0	17
Tripotent	3.7	3

Supplemental Figure 5

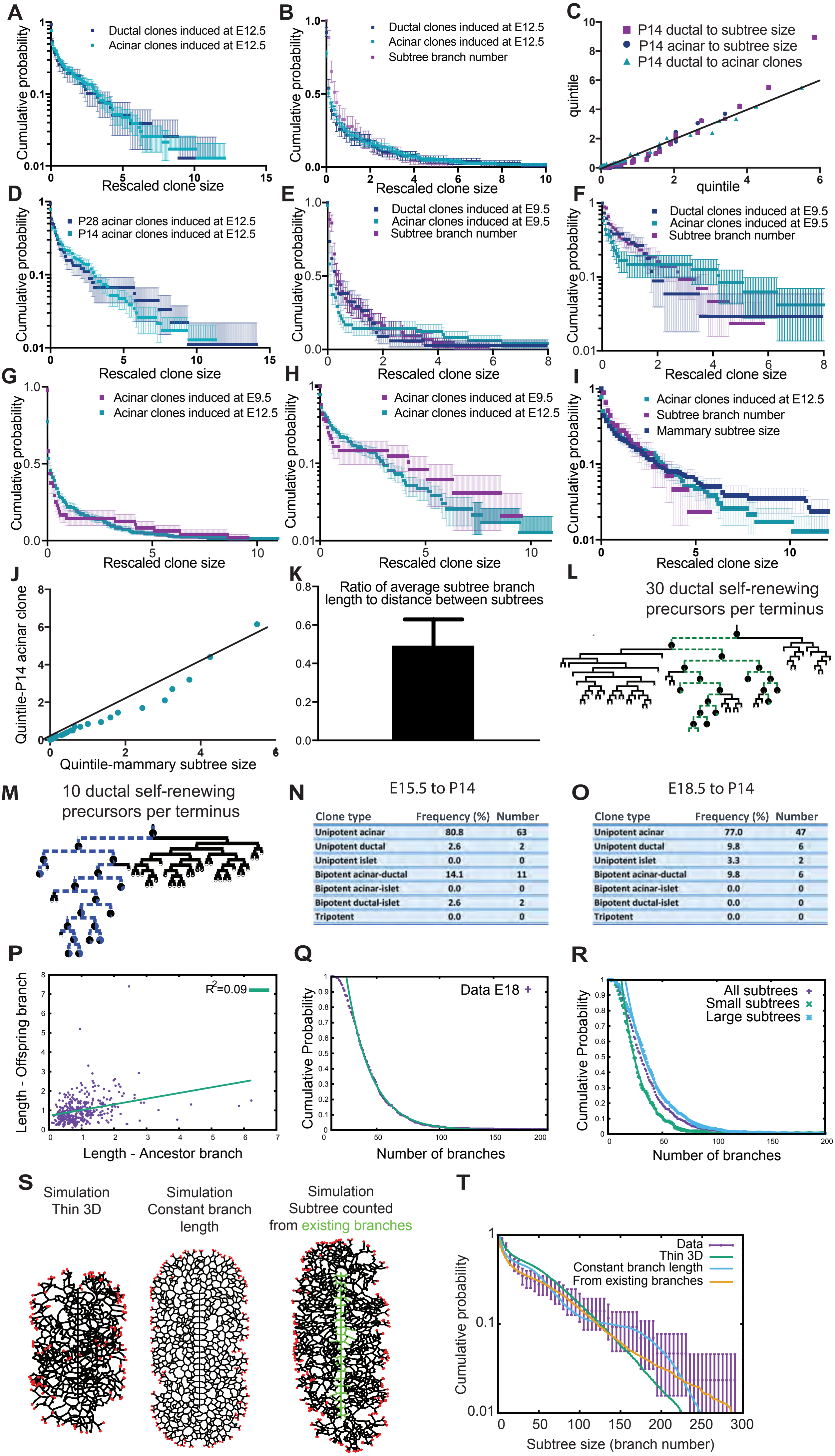

N E15.5 to P14

Clone type	Frequency (%)	Number
Unipotent acinar	80.8	63
Unipotent ductal	2.6	2
Unipotent islet	0.0	0
Bipotent acinar-ductal	14.1	11
Bipotent acinar-islet	0.0	0
Bipotent ductal-islet	2.6	2
Tripotent	0.0	0

O E18.5 to P14

Clone type	Frequency (%)	Number
Unipotent acinar	77.0	47
Unipotent ductal	9.8	6
Unipotent islet	3.3	2
Bipotent acinar-ductal	9.8	6
Bipotent acinar-islet	0.0	0
Bipotent ductal-islet	0.0	0
Tripotent	0.0	0

Supplemental Figure 6

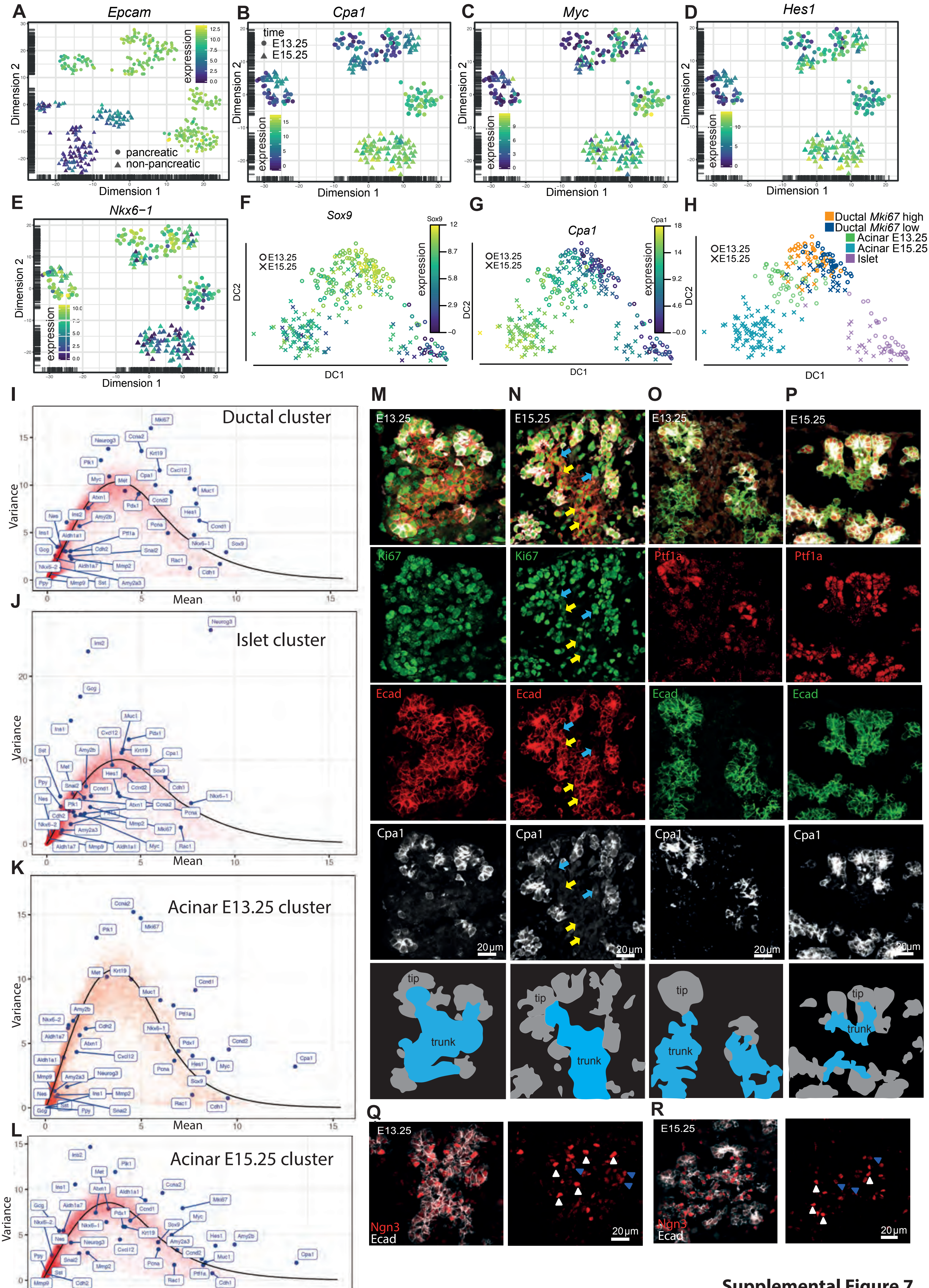

Supplemental Figure 7

Supplemental Figure 1. Related to Figures 1 and 2. Typical examples of acinar, ductal and islet clones.

(**A**) The basis of the *Rosa26*-Confetti reporter system and fluorescent protein expression (nuclear GFP – nGFP, cytoplasmic YFP – cYFP, cRFP, and membrane CFP – mCFP). (**B,C**) Single-cell ductal clones, (**D-F**) ductal monoclonal clones, (**G-I**) subtree-associated acinar-majority clones, and (**J-L**) ductal terminus-associated clones with (J) islet contribution – cYFP clone. (**M-O**) Thick 100µm sections of P14 pancreas of *Rosa26*-Cre; *Rosa26*-Confetti mice induced at E12.5 that were used for clonal analysis. The images were created with the tiling panel on a confocal microscope Leica SP5 and reveal the morphology of clones. Ducts, islets and acini indicated with arrow, arrowhead and asterisk, respectively.

Supplemental Figure 2. Related to Figure 1. Verifying the integrity of clonal assignments and robustness of the potency results.

(**A,B**) 100µm pancreatic sections from *Rosa26*-CreT2; *Rosa26*-Confetti (n=3mice) and (**C,D**) *Sox9*-CreERT2; *Rosa26*-Confetti (n=3mice) non-injected mouse at, respectively, P14 and P28 shows that the constructs were not leaky. (**E**) Schematic indicating methods of clonal assignment based on cohesiveness and distances between constituent labeled cell clusters (Theory Methods). (**F**) Rescaled probability (purple thick line) of finding another clone of the same color as a function of rescaled distance from a given clone for all three *Rosa26*-Confetti mice induced at E12.5 and traced until P14 (left, center and right panels). For each mouse, the clone-clone distance was rescaled by the average distance in three-dimensions between two clones randomly chosen across sections of a given pancreas. The probability of finding a clone of the same color at a given distance was rescaled by the probability of finding two clones of any color at the same distance. The shaded lines represent the 95% confidence interval of rescaled probability under the assumption that the data was clonal. The dashed black line shows the distance used for fragment grouping to define a clone. (**G**) Fraction of fluorescently labeled cells at E12.5 in the P14 pancreas of different mice. (**H**) Color balance of confetti clones induced at E12.5 and traced until P14, grouping all three pancreases. Cyan is the least represented color while Red is the most represented. (**I**) Global potency of P14 clones induced at E12.5, segregated for each individual mouse, showing that the potency of each is consistent with the ensemble average (Figure 1K), and independent of the frequency of induction shown in H. (**J**) Global potency of P14 clones induced at E12.5, segregated for each individual confetti color. (**K**) Percentage of pancreas that belongs to the ductal compartment at P14. (**L**) Fold increase in the size of the pancreas as a whole and in the acinar compartment of clones between E13.5 and P14, and E13.5

and P28, indicating that clones are representative of tissue. Bars indicate mean and S.E.M. Growth characteristics of the murine pancreas including (**M**) changes in pancreatic mass between E18.5 and P28; (**N**) fold-change in pancreas width, length and height at E13.5, P14 and P28, as defined in the schematic inset. Bars indicate mean and S.E.M. Frequency of different clone types by cell composition for (**O**) E12.5 induction with 53 clones (*n=4* mice) quantified with additional amylase co-immunostaining in additional to DBA and chromogranin A, and (**P**) E12.5 induction with 254 clones (*n=3* mice) quantified (same dataset as Figure 1K).

Supplemental Figure 3. Related to Figure 1. Additional verification of clone types with amylase immunostaining.

(**A,B**) Branch-associated cRFP clones in 3D projection and in (**A',B'**) individual Z-stack zoomed from the blue box in (A,B) reveal complete overlap with the acinar amylase immunostaining (green). (**C**) shows a 3D projection of a tripotent clone with a ductal branching component (re-stained clone from Figure 2I) with individual Z-stack sections presented in (**C',C''**) zoomed from a blue box in (C). (C') shows a clone part overlapping completely with the ductal DBA (white), and showing no overlap with acinar amylase (green). (C'') shows a clone part overlapping with amylase (green, arrowhead) and a part overlapping with islet (white, arrow). (**D**) 3D projection of ductal branching clone (re-stained clone from Figure S1K) with individual Z-stack sections presented in (**D',D''**) zoomed from the blue box in (D). (D',D'') show complete overlap of a clone with the DBA (white) ductal labeling. DBA, chromogranin A are grey, amylase is green, DAPI is blue. Note that individual nuclei can be observed with cytoplasmic amylase surrounding the nucleus in a cloud-like fashion.

Supplemental Figure 4. Related to Figure 1. Verification of marker staining.

(**A**) Combination of two markers for islet chromogranin A (green) and ductal DBA (white) alone shows that the two compartments display clear morphological differences and therefore can be stained in one channel for potency analysis of confetti+ cells. Since hallmark morphological differences distinguish ductal and islet compartments, in the clonal lineage study, these features can be imaged using the same channel. This allows us to reserve other channels for DAPI staining and to discriminate all of the fluorescent reporters used in the confetti tracing. (**B**) Combination of all three markers with islet chromogranin A (green), ductal DBA (white) and acinar amylase (pink) shows that all the markers are exclusive and the vast majority of DBA-

chromogranin A- tissue is amylase+. (**C**) The combination of two markers for islet chromogranin A (white) and acinar amylase (green), shows complete mutual exclusivity. Visualization showing exclusive intensity ranges distinguishing imaging of ducts and islets, following careful optimization of the immunostaining protocol. The islets were stained with DBA in 647nm and chromogranin A in both 488nm and 647nm. (**D-F**) Various examples of islets and ducts from left to right for the same section: (i) overlay of white (647nm channel) and green (488nm channel) shows the distinction between the islets and the ducts; (ii) green (488nm) shows islets only; (iii) white (647nm) raw image shows the combination of islet and ductal staining, revealing a lower intensity of islet staining (dimmer area) than the ducts (significantly brighter); (iv-v) representation of image from (iii) white (647nm) channel in (iv) ultra and (v) spectral intensity mode in Imaris, demonstrating that the two pancreatic structures are stained at distinct and exclusive intensities showing a perfect match with the chromogranin A and DBA stained in two distinct and well-separated imaging channels (488nm and 647nm), as in (i).

Supplemental Figure 5. Related to Figures 1 and 3. Quantification of clone types with their relative frequency and respective sizes.

(**A**) Complete size distribution for all clones induced from E12.5 to P14. (**B**) Comparison of the same clones as (A) but segregating unipotent and multipotent clones. Average size +/-S.D. are shown. (**C**) Comparison of branch number of branching clones in outer, middle and inner third of the thick tissue clones reveals statistically significant difference between outer and middle/inner thirds ($p < 0.01$ – Mann-Whitney). Average branch numbers +/-S.E.M. are presented. (**D**) Table of all clone types traced from E12.5 to P28. (**E**) Global potency of clones traced from E12.5 to P28. (**F**) Table of all clone types traced from E9.5 to P14. (**G**) Potency of acinar-containing clones traced from E9.5 to P14. (**H**) Potency of ductal-containing clones traced from E9.5 to P14. (**I,J**) Comparison of the fraction of branched clones (I) and average number of branches per clone (J) from the E12.5, E15.5 and E18.5 tracings (all traced until P14). Branched clones are defined as those that belong to individual subtrees with at least 3 ductal branches containing labeled cells of the same color, or 3 acini clusters at the termini of different branches containing labeled cells of the same color. Averages +/- S.E.M are shown. (**K-O**) Representative images of *Sox9*-CreERT2 traced clones recapitulating the acinar and ductal branching clones observed with *Rosa26*-CreERT2 tracing. (K,L) presents acinar tree shaped clones surrounding the network of ducts with individual Z-stacks presented in (**K',L'**). (M-O) Clones with a ductal branching element. (M) Ductal-islet bipotent clone with individual Z-stack images presented in (**M',M''**) – islet part in (M') and ductal part in (M''). (N,O) Ductal-only clones with individual Z-stacks presented in (**N'**) and (**O',O''**)

showing co-localization with DBA and no co-localization with amylase. (**P**) Potency quantification for *Sox9*-CreERT2 lineage tracing between E12.5 and P14. (**Q**) Frequency and number of individual clone types for *Sox9*-lineage tracing. (**R**) Comparison of E12.5 to P14 *Rosa26* and *Sox9* lineage tracing reveals statistically significant difference in the potency outcome. (**S**) Fraction of clones with individual compartments present (acinar, ductal, islet). *Sox9*-traced clones reveal an enrichment in the ductal fraction.

Supplemental Figure 6. Related to Figures 1,4 and 5. Comparison between subtree size distributions and clone size distribution at E9.5 and E12.5.

(**A**) Cumulative clone size distribution (plotted on a logarithmic scale) of ductal and acinar compartments from E12.5 induction. Clone sizes here and below were rescaled by their respective ensemble averages. S.D are shown in all cumulative clone size distributions. (**B**) Cumulative clone size distribution (plotted on linear scale) of ductal and acinar compartments from E12.5 induction overlaid with the cumulative subtree size distribution (number of branches) determined from E18.5 pancreas. (**C**) Quintile-quintile plot comparing cumulative size distribution of P14 ductal compartment clones (induced at E12.5) versus P14 acinar compartment clones (induced at E12.5) in cyan; P14 ductal compartment clones (induced at E12.5) versus pancreas subtree size (at E18.5) in pink; P14 acinar compartment clones (induced at E12.5) versus pancreas subtree size (at E18.5) in dark blue. (**D**) Cumulative size distribution (plotted on logarithmic scale) of rescaled P14 and P28 clone sizes both induced at E12.5. (**E,F**) Cumulative size distribution (plotted on linear and log y-axis) of ductal and acinar compartments in clones induced at E9.5 overlaid with the cumulative subtree size distribution (number of branches) determined from E18.5 pancreas. (**G**,**H**) Cumulative size distribution (plotted on linear and logarithmic scale) of acinar compartments in clones induced at E9.5 and E12.5. (**I**) Cumulative size distribution (plotted on logarithmic scale) of acinar compartments in clones induced at E12.5, pancreatic subtree size and mammary gland epithelium subtree size (Scheele et al. 2017), showing similar growth characteristics between developing pancreas and mammary gland. (**J**) Quintile-quintile plot comparing the cumulative size distribution of P14 acinar compartment clones (induced at E12.5) versus experimental mammary subtree size (Scheele et al. 2017). (**K**) Ratio of the average branch length (distance between two branch points) by the average distance between two neighboring subtrees (along the main duct), quantified in E18 pancreas, used as an input for the simulations of Figure 4F,G. Average ratio +/- S.E.M. are shown. (**L,M**) Simulation of a theoretical subtree containing (L) 30 and (M) 10 precursors per active ductal terminus with one labeled self-renewing precursor cell. (**N**) Table of all clone

types traced from E15.5 to P14. (**O**) Table of all clone types traced from E18.5 to P14. (**P**) Relationship between the length of a branch and the length of its offspring branch, showing little correlation. (**Q,R**) Branch length distribution across all subtrees (Q) or segregated between small and larger subtrees (R), showing a consistently good fit by an exponential with small waiting time (thick lines), indicative of a stochastic branching process. (**S,T**) Sensitivity analysis for the branching-annihilating random walk (BARW) simulations, considering alternative models, shown as characteristic simulation output (S) or subtree size distribution (right, 500 simulations each). For left to right: simulation in a thin 3D setting (green theory line), for constant branch length (blue theory line) and for subtrees arising from existing branches (generation 1, shown in green) (orange theory line). The simulations show that these small variations, keeping the core of BARW hypothesis intact, do not significantly impact the predictions.

Supplemental Figure 7. Related to Figure 6. Single-cell RNA sequencing – gene-specific graphs.

(**A-E**) t-SNE plots, where each cell is colored by the log-normalized expression level of (A) *Epcam,* (B) *Cpa1,* (C) *Myc,* (D) *Hes1,* (E) *Nkx6-1*, enabling recognition of (B,C) the acinar cluster together with Figure 6C, and (D,E) the ductal cluster together with Figure 6D. (B-E) t-SNE plots contain E13.25 (circle) and E15.25 (triangle) cells showing close clustering of cells from the two time-points; in (A) circles and triangles represent pancreatic and non-pancreatic cells. The shape indicates cluster association as determined by k-means clustering. (**F-H**) Diffusion pseudotime (DPT) plots with both E13.25 (circles) and E15.25 (crosses) cells indicating that (F) *Sox9* is anti-correlated ($P < 5.5\ 10^{-12}$) with DPT direction, i.e. expression is upregulated at the apex, while (G) *Cpa1* is correlated ($P < 5.5\ 10^{-12}$) with DPT direction, i.e. expression is upregulated in differentiating cells. (F-G) Each cell is colored by the log-normalized expression level. (H) DPT plot for *Mki67* showing *Mki67* high cells segregating towards acinar branch and *Mki67* low cells segregating towards islet branch, consistent with the lineage tracing data, which indicate that acinar and ductal cells retain full proliferative potential. (**I-L**) Variance of genes (red) in a given cluster as a function of the mean expression level for (I) ductal, (J) islet, (K) acinar E13.25, and (L) acinar E15.25 clusters, revealing that *Cpa1* and *Sox9* are indeed heterogeneously expressed in ductal cells (I), *Sox9* is homogeneous in acinar cells (K,L), and *Ptf1a* shows homogenous expression in the E15.25 acinar cluster (J). *Ngn3* is expressed heterogeneously within the islet cluster (J), consistent with the literature (Bankaitis et al. 2015). (**M-R**) Immunostained 7µm thin pancreatic sections of Ecadherin, Cpa1 and Ki67 at (M) E13.25 and (N) E15.25 showing enrichment in Ki67 in Cpa1+ tip areas at E15.25. In Ecadherin+ Cpa1- trunk regions,

there is a slight enrichment of Ki67-high cells at peripheral regions indicated by blue arrows, while Ki67-low cells in more central parts are indicated by yellow arrows. (**O,P**) E-cadherin, Cpa1 and Ptf1a immunostaining at (O) E13.25 and (P) E15.25 shows a relatively homogenous expression of Ptf1a, which also overlaps with Cpa1 expression. (**Q,R**) E-cadherin and Ngn3 immmunostaining reveals Ngn3 expression heterogeneity (as previously reported, Bankaitis et al. 2015) with Ngn3-high cells indicated by blue arrowhead and Ngn3-low cells indicated by white arrowheads at (Q) E13.25 and (R) E15.25.

Methods S1. Related to STAR Methods

Theoretical analysis of branching morphogenesis and monoclonal conversion

Pancreas development involves a two-stage process in which a phase of plexus formation and remodeling precedes a phase of branching morphogenesis (although some temporal overlaps may exist between the two phases, Bankaitis et al. 2015). Although cell fate behavior and clonal dynamics during the phase of plexus remodeling is challenging to resolve, the observed localization of clonal fragments to individual ductal subtrees (see main text) indicates that the large-scale structure of the pancreas is dominated by the secondary phase of branching morphogenesis involving serial rounds of dichotomous ductal bifurcation. In the following, the strategy to define the dynamics of pancreatic progenitors during this secondary phase of expansion is detailed, focusing on clonal data obtained from the E12.5 induction time-point. In developing this program, use was made of a model introduced recently to describe the process of branching morphogenesis in several mammalian organs, including the mouse mammary gland epithelium and kidney, as well as human prostate (Hannezo et al. 2017). Specifically, in the context of the mammary gland (Scheele et al. 2017), it has been shown that the statistics of the branched ductal network are consistent with a remarkably simple paradigm in which functionally equivalent (i.e. equipotent) “active” terminal end-buds of ducts choose stochastically between ductal bifurcation and termination (inactivation), where further bifurcation ceases. Based on the success of this model in predicting quantitatively the statistical distributions of branching patterns and clonal data (Hannezo et al. 2017), here the same basic paradigm was tested to determine if it could capture quantitatively the branching dynamics of pancreatic morphogenesis.

In particular, our modeling approach was underpinned by two key observations: First, at E18, the ductal network in pancreas appeared surprisingly two-dimensional, with very few “cross-overs”, where ducts would stand on top of one another in close proximity, a behavior reminiscent of the mammary gland epithelium. Second, there was huge subtree size heterogeneity within individual ductal trees emanating from the main central ducts (primordial structures derived from reorganization of the central plexus). In particular, some subtrees terminated after just a few branches, while others branch extensively, colonizing large fractions of a given pancreatic lobe (see Figure 4B,C for quantification). In the case of purely ductal end-driven morphogenesis, this suggests that some ductal ends “terminated” early during development, while others continued to branch and expand, with the history of branching decisions encoded in the topology of the ductal network.

A branch generation, *n*, was defined as the number of branch points separating a given branch from the central ducts from which branching morphogenesis initiates. This generation number could be defined uniquely since, in common with the mammary gland, pancreatic ductal trees form simple graphs, i.e. notably, loops or reconnections at E18.5 between two independent subtrees were not observed starting from the main trunk (although some loops can be found within the central plexus at earlier time-points). The central plexus was defined manually and subtrees were defined as deriving from the first generation of branches that bud from this central plexus. From a physical perspective, it was clear that there must be some termination events occurring; otherwise, the number of ductal termini at generation *n* would grow exponentially as 2^n. This is not tenable for ducts whose thickness and length are largely independent of the generation number, as observed in both mammary gland and pancreas. Therefore, some ducts must terminate, accounting for the extreme heterogeneity in subtree sizes.

Based on these findings, the further observation that branching morphogenesis takes place in a largely two-dimensional setting, without ductal cross-overs, offers a crucial simplification: Strikingly, when examining small ductal subtrees in pancreas, i.e. ones that had likely terminated early, they appeared to be "trapped" by the spatial constraints imposed by neighboring subtrees. A similar organization is visible in the mammary gland (where secreted TGF-β1 is thought to mediate a feedback signal between ducts and active terminal end-buds), and offers a simple heuristic explanation for how environmental signals can provide a feedback mechanism to arrest ductal bifurcation, viz. ductal termination (Hannezo et al. 2017).

Thus, based on these observations, branching morphogenesis in pancreas is proposed to occur according to three simple local "rules" for the dynamics of active ductal termini that together translate into the formation of the large-scale structure of the organ (see Figure 4E for a sketch):

- An active ductal terminus, *A*, undergoes a persistent random walk at speed *v* and persistence time, t_p, and thus randomly explores space is an isotropic (diffusive) manner, leaving behind a trail of "inactive" ducts via cell proliferation. Note that, while the further proliferation of cells in "inactive" ducts may contribute to local dilation and expansion of ducts, the large-scale organization of the ductal network is preserved.
- An active ductal terminus has a probability r_b, presumed constant in time, to branch into two new active ductal termini. One would thus predict, for constant speed *v*, an exponential distribution of branch size, a feature reproduced well by the experimental data at all length scales above a cut-off at around 20μm (presumably set by the tip/cell size, Figure S6Q).

- An active ductal terminus becomes inactive when it comes into “contact” with another duct, thereby preventing ductal crossovers in the predominantly two-dimensional geometry of the embryonic pancreas. The diffusing signals from maturing ducts may enlarge the contact radius, r_a, to length scales much larger than a cell diameter; but it is still local on the length-scale of the average distance between consecutive branching events.

Note that an important feature of such a model – known as a branching-annihilating random walk – is that it considers all ductal termini to be functionally equivalent, i.e. having an equal probability *a priori* to give rise to a small or large ductal subtree. It is only thought the chance dynamics of the random walk and competition for space with neighboring ductal termini and ducts that subtrees acquire their size heterogeneity. This results in subtree size distributions, expressed in terms of the total number of branches in a given subtree, which are characteristically broad with an exponential tail, fitting qualitatively with the experimental data. Within this framework, most subtrees stochastically stop contributing to branching morphogenesis early in development, while an ever-diminishing surviving population of active ductal termini end up giving rise to nearly all the peripheral regions of the organ.

Comparison between model and experiment

To go beyond such qualitative comparisons, full numerical simulations of the three processes described above were employed. To adapt to the specificity of pancreatic morphogenesis, instead of starting from a single active terminus, as in the study of mammary gland or kidney morphogenesis (Hannezo et al. 2017), an initial condition was taken as a situation in which a central duct is already formed, as in Figure 3H (Figure 4F, left-most panel). In particular, model simulations were performed with 20 active termini located at each side of the central duct, facing outwards, and with a constant spacing L. Note that the ratio, Lr_b/v, defines the only key parameter of the model, comparing the typical branch size to the initial spacing between subtrees. This parameter was calculated experimentally (Figure S6K) and set accordingly in the simulation.

Running the simulation until subtrees reached the average subtree size measured in E18 pancreas networks (Figure 4F, right-most panel), trees were found to self-organize into a pulse of active termini located at the periphery of the growing network, leaving in their wake an internal structure of roughly constant ductal density, as expected from previous work (Hannezo et al. 2017). This organization strongly echoes the results of the EdU kinetics experiments (Figure 4I-N), showing enhanced ductal proliferation at the

periphery of the pancreas during branching morphogenesis. Because of the initial condition chosen, the final configuration of the pancreas was an ellipsoid shape, with large subtree size heterogeneity. To compute with high accuracy the predicted subtree size distribution, averages were made over 500 such simulations. N=20 subtrees were simulated to grow on each side of a central duct. Strikingly, comparing the model prediction with the E18 subtree size distribution data, an excellent agreement (R^2=0.99, S=0.03) was found, arguing that this minimal model is sufficient to recapitulate the statistical heterogeneity of branching patterns and thus, as a consequence, the clone size distributions. Importantly, as Lr_b/v is the key parameter of the model, it was questioned how changes in this parameter would change the predictions of the model. The experimentally measured variance of this parameter (Lr_b/v=1.45+/-0.5, mean +/- 2S.D.) was used to build 95% confidence intervals for the model predictions (see thin lines on Figure 4G), keeping the total simulation time constant for all parameters. The other parameters of the model were set as $r_a r_b/v$=0.25 and a small rotational diffusion (dictating persistence) of π/10. Nevertheless, it should be noted that the exact values of these parameters are largely irrelevant on the resulting dynamics, as it was shown previously via sensitivity analysis that variations of these two parameters had only a very weak effect on the predicted branched structure (Hannezo et al. 2017).

To further demonstrate the robustness of the branching and annihilating random walk model, a number of additional simulations were performed as controls (Figure S6S,T). In particular, a small 3D exploration of space was allowed, to simulate ductal branching within a thin quasi 2D setting, where there is a minimal, but non-vanishing, probability for overlap between ducts. The case was also considered where subtrees where not defined as emerging from the main central duct, but one generation onwards (in green in Figure S6S), to test the effect of a different initial condition (representing for instance a central plexus). Finally, the effect of a constant branch length was simulated (instead of an exponential distribution resulting from stochastic branching at any time-point). Crucially, it was found that the key features of the model, i.e. patterning of the tree via a self-organized propagating wave of peripheral growth, and large-scale heterogeneity of the subtree branching patterns, were conserved in all these variations of the model, suggesting that branching and annihilation were the key features giving rise to the observed phenomenology.

Whether some of the heterogeneity in branch length could be explained by positional effects was also tested. In particular, the distribution of branch lengths in “small” (<30 branches) versus “large” (>60 branches) subtrees (Figure S6R) were analyzed. Interestingly, although there was a slight increase of branch length in large subtrees (Figure S6R), both distributions were well-fit by exponential distributions. We note that this difference in

average size could be due to a slightly larger *v* (or r_b) for some subtrees; but, alternatively, could be due to subsequent growth/remodeling events within the pancreas. Finally, to probe this question of neutrality further, the correlation in branch lengths of consecutive branches (across all subtrees) was probed and confirmed that it displayed very poor correlation (R^2=0.09 from linear regression, see Figure S6P).

Overall, these results argue that branching morphogenesis in the pancreas and mammary gland, which dominates the large-scale organization of both tissues, is controlled by a similar self-organizing principle, involving sequential rounds of ductal bifurcation checked by ductal termination mediated by local signals from neighboring ducts. Further analogy between mammary gland and pancreatic branching morphogenesis is found at the cellular level. Indeed, the gradual transition of self-renewing multipotent progenitors to fate-restricted ductal- and acinar-committed progenitors during pancreas development mirrors the behavior observed in mammary gland, where bipotent precursors segregate early into fate-restricted self-renewing basal and luminal progenitors, both residing within terminal end-buds and undergoing the same collective branching/termination dynamics.

Theoretical analysis of monoclonal conversion during branching morphogenesis

On this background, the clonal dynamics of self-renewing pancreatic progenitors was then assessed within each individual ductal terminus. Indeed, the above description only deals with an effective description of the collective fate choices made by all progenitors within a given terminus, without making any assumptions or predictions related to the actual number of such progenitors in each terminus. However, the clonal lineage tracing data provides a platform to address this question in further detail. Again, as described previously in the context of the mammary gland epithelium (Scheele et al. 2017), it is supposed that ducts consist of *N* equipotent self-renewing progenitors, which are randomly allocated to one of the two offspring ducts upon bifurcation, such that each new duct received *N/2* such self-renewing progenitors. One should note that, relaxing this equal repartition assumption does not give rise to qualitatively different behavior to the one we describe below. Each progenitor then divides once to expand the pool so that there are, again, *N* progenitors per terminus – the process of renewal. Interestingly, even in the absence of any active competition between progenitors within the terminus, this process would drive monoclonal conversion of lineage-labeled ducts. This is because, upon bifurcation, a clone will be randomly segregated, and thus enriched in one of the termini, and depleted in another. In the limit of randomly allocated cells, i.e. the limit of

strong cell mixing, which is relevant here (as we do not see cohesive streams of clones before monoclonal conversion), monoclonal conversion is expected to occur slowly, as average clone sizes increase only linearly with the number of ductal bifurcations. Therefore, with say $N=30$ progenitors per terminus, monoclonal conversion would almost never be observed over the time-scales relevant for pancreatic morphogenesis (Figure 5C and S6L,M). This is in stark contrast to the experimental data, which is characterized by a seemingly very rapid process of monoclonal conversion of ducts (Figure 5C and S1J-L), on the scale of just a few generations of ductal branching. Therefore, this heavily constrains the number of self-renewing progenitors that are located in each individual ductal terminus. Indeed, simulations of as few as $N=4$ progenitors per ductal terminus yield a rate of monoclonal conversion that is compatible with the experimental data (Figure 5B,C).

One should note that active cell competition during ductal bifurcation would speed up the process of monoclonal conversion, but would not yield qualitatively different results, as average clones sizes would still increase only linearly with generation number. Interestingly, such a behavior of monoclonal conversion of entire ductal subtrees of the pancreas, seen both from ductal-containing clones and acinar-containing clones, validates *a posteriori* such a ductal end-splitting mechanism of morphogenesis.

Lastly, in developing a model of branching morphogenesis, changes that occur during development in the lineage potential of self-renewing progenitors at the ductal termini have not been considered explicitly. From measurements of the clone composition, it is known that termini can play host to multipotent and/or fate-restricted self-renewing progenitors, with the abundance of the former diminishing through development. Clearly, as with the basal and luminal compartments in developing mammary gland epithelium, providing all progenitors at the termini are renewed during the bifurcation process, the branching-annihilating random walk model is agnostic to the potency of the self-renewing population. However, this condition provides a significant constraint on the regulation of the termini supported by purely fate-restricted progenitors – the majority at the later stages of branching morphogenesis. In this case, the niche environment at the ductal termini must be capable regulating independently the multiplicity of both ductal/islet progenitors and acinar-committed progenitors to ensure their renewal during bifurcation, a constraint most easily engineered by having one compartment provide a niche for the other. An interesting extension for further studies would be to refine this model to include explicitly the time-dependent dynamics of multipotency, i.e. how the fate transitions between different cell types occur and are regulated spatio-temporally during branching morphogenesis.
