# Supplementary material for: Defining Lineage Potential and Fate Behavior of Precursors during Pancreas Development
Source: Dev Cell. 2018 Aug 6;46(3):360–375.e5. doi: 10.1016/j.devcel.2018.06.028 (PMC6085117; doi:10.1016/j.devcel.2018.06.028)
Supplement: Document S2. Article plus Supplemental Information [file mmc7.pdf]

Article

Developmental Cell

Defining Lineage Potential and Fate Behavior of Precursors during Pancreas Development

Graphical Abstract

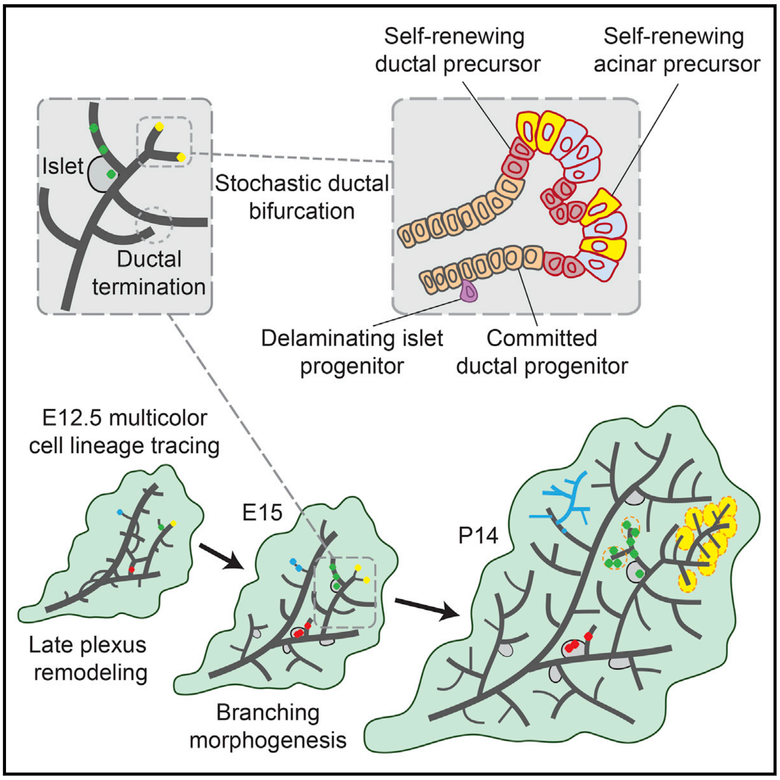

Authors

Magdalena K. Sznurkowska, Edouard Hannezo, Roberta Azzarelli, ..., Meritxell Huch, Anna Philpott, Benjamin D. Simons

Correspondence

ap113@cam.ac.uk (A.P.),
bds10@cam.ac.uk (B.D.S.)

In Brief

By combining clonal tracing and whole-mount reconstruction with proliferation kinetics and single-cell transcriptional profiling, Sznurkowska et al. show that the large-scale organization of mouse pancreas can be traced to the activity of self-renewing precursors positioned at the termini of growing ducts.

Highlights

- The pancreas undergoes rapid cell fate specification in development
- Ductal ends serve as a niche site for self-renewing progenitors
- These cells drive rounds of stochastic ductal bifurcation balanced by termination
- These results provide a model of coordinated development of acini, ducts, and islets

Sznurkowska et al., 2018, Developmental Cell *46*, 360–375
August 6, 2018 © 2018 The Author(s). Published by Elsevier Inc.
https://doi.org/10.1016/j.devcel.2018.06.028

CellPress

Developmental Cell
Article

Defining Lineage Potential and Fate Behavior of Precursors during Pancreas Development

Magdalena K. Sznurkowska,[1,2] Edouard Hannezo,[2,3,4,7] Roberta Azzarelli,[1,2] Steffen Rulands,[3,4] Sonia Nestorowa,[5] Christopher J. Hindley,[3] Jennifer Nichols,[2,6] Berthold Göttgens,[5] Meritxell Huch,[3] Anna Philpott,[1,2,*] and Benjamin D. Simons[2,3,4,8,*]
[1]Department of Oncology, University of Cambridge, Hutchison/MRC Research Centre, Cambridge Biomedical Campus, Cambridge CB2 0XZ, UK
[2]Wellcome-MRC Cambridge Stem Cell Institute, University of Cambridge, Tennis Court Road, Cambridge CB2 1QR, UK
[3]The Wellcome Trust/Cancer Research UK Gurdon Institute, University of Cambridge, Tennis Court Road, Cambridge CB2 1QN, UK
[4]Cavendish Laboratory, Department of Physics, University of Cambridge, JJ Thomson Avenue, Cambridge CB3 0HE, UK
[5]Department of Haematology, Cambridge Institute for Medical Research, Wellcome Trust/MRC Building, Cambridge Biomedical Campus Box 139, Hills Road, Cambridge CB2 0XY, UK
[6]Department of Physiology, Development, and Neuroscience, University of Cambridge, Tennis Court Road, Cambridge CB2 3EG, UK
[7]Institute of Science and Technology IST Austria, 3400 Klosterneuburg, Austria
[8]Lead Contact
*Correspondence: ap113@cam.ac.uk (A.P.), bds10@cam.ac.uk (B.D.S.)
https://doi.org/10.1016/j.devcel.2018.06.028

SUMMARY

Pancreas development involves a coordinated process in which an early phase of cell segregation is followed by a longer phase of lineage restriction, expansion, and tissue remodeling. By combining clonal tracing and whole-mount reconstruction with proliferation kinetics and single-cell transcriptional profiling, we define the functional basis of pancreas morphogenesis. We show that the large-scale organization of mouse pancreas can be traced to the activity of self-renewing precursors positioned at the termini of growing ducts, which act collectively to drive serial rounds of stochastic ductal bifurcation balanced by termination. During this phase of branching morphogenesis, multipotent precursors become progressively fate-restricted, giving rise to self-renewing acinar-committed precursors that are conveyed with growing ducts, as well as ductal progenitors that expand the trailing ducts and give rise to delaminating endocrine cells. These findings define quantitatively how the functional behavior and lineage progression of precursor pools determine the large-scale patterning of pancreatic sub-compartments.

INTRODUCTION

With the increasing prevalence of diabetes mellitus, interest in the development and function of the pancreas remains intense (Edlund, 2001). Although much has been learned about the molecular regulators of exocrine and endocrine specification, our understanding of the morphological events that generate the patterned organ remain limited. However, using a combination of immunohistochemistry and functional genetic lineage tracing assays in mice, the pathways involved in the differential fate specification of the embryonic pancreas have begun to emerge (Zhou et al., 2007; Johansson et al., 2007; Solar et al., 2009; Schaffer et al., 2010; Kopp et al., 2011; Kopinke et al., 2011; Pan et al., 2013).

In mice, the program of pancreas specification is initiated around embryonic day (E) 8.5. This is followed by the formation of ventral and dorsal buds from either side of the foregut at E9.5–10 (Pan and Wright, 2011; Shih et al., 2013). At this stage of development, epithelial cells are thought to retain multi-lineage potential, capable of differentiating into all three major pancreatic cell types: ductal, acinar, and islet (Pan and Wright, 2011; Shih et al., 2013). Around E12.5, the developing pancreas transfers into a "secondary transition" phase in which the expression profiles of precursors segregate into "tip-trunk" domains, orchestrated by Nkx6.1/Ptf1a cross-repression (Schaffer et al., 2010), forming a central multi-lumen tubular plexus (Pan and Wright, 2011) (Figure 1A). Then, through an extensive phase of branching morphogenesis, outgrowths from the plexus expand into an arbor of growing pancreatic ducts.

During the phase of tip-trunk segregation, tip cells are thought to become restricted to acinar fate (Zhou et al., 2007), while trunk cells give rise to ductal cells as well as Ngn3+ endocrine-committed cells (Solar et al., 2009; Johansson et al., 2007) (Figure 1A). However, lineage tracing studies based on Cpa1 expression in tip cells and Hnf1β expression in trunk cells suggest that both domains retain tripotent precursors until at least E13.5 (Zhou et al., 2007; Solar et al., 2009). Indeed, tracing studies of Sox9-expressing cells suggest that tripotent cells may persist even after birth (Kopp et al., 2011; Furuyama et al., 2011). Yet, since these genetic labeling studies have sought to address the lineage potential of cells at the population scale (Zhou et al., 2007; Solar et al., 2009; Kopp et al., 2011), the question of whether and how these findings hold at the level of individual cells remain unclear. Recently, a clonal lineage tracing study based on the use of ubiquitous and gene-specific promoters has argued that pancreatic founder cells may become sub-lineage-restricted as early as E9.5 (Larsen et al., 2017), with evidence

This is an open access article under the CC BY license (http://creativecommons.org/licenses/by/4.0/).

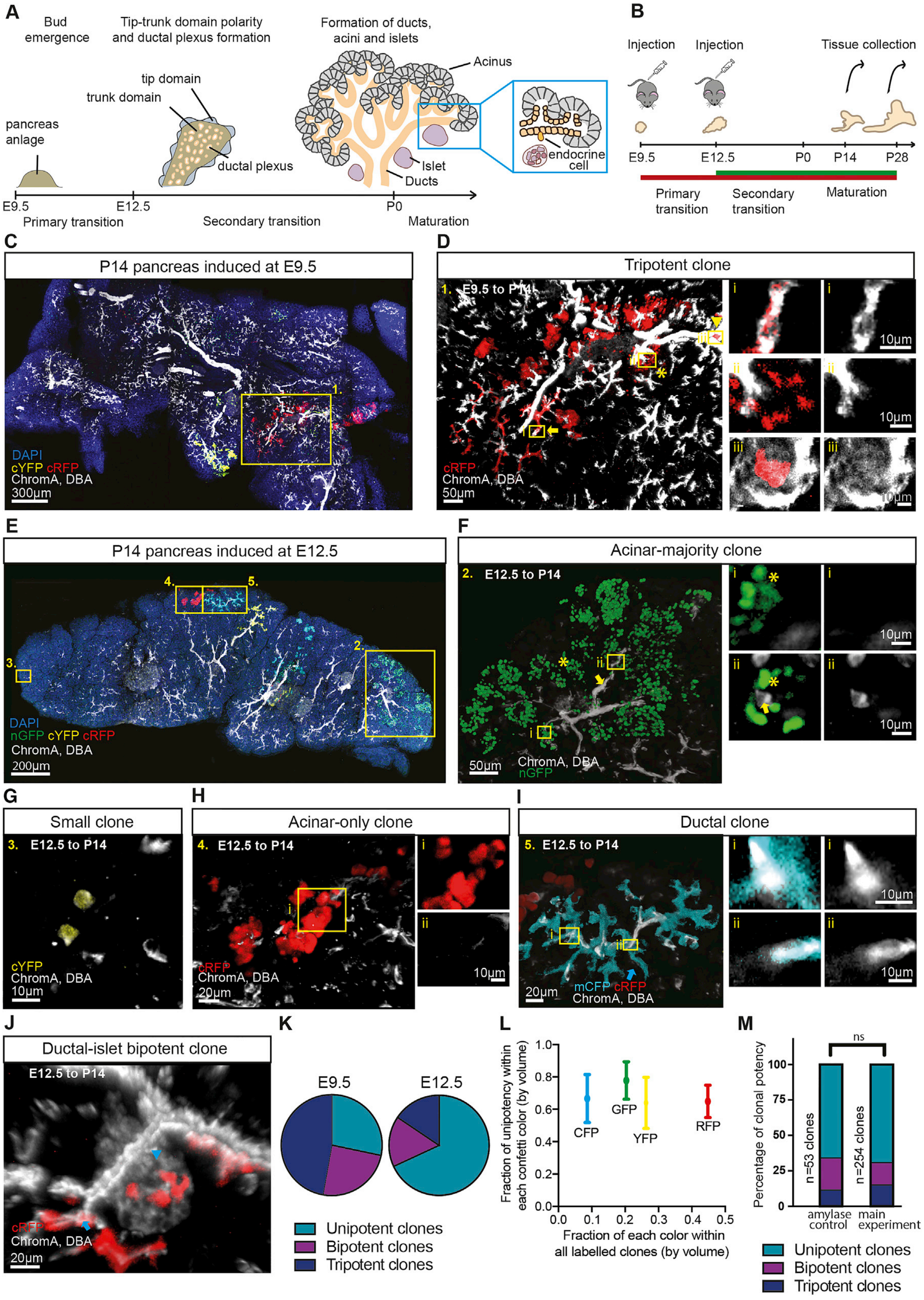

(legend on next page)

for early commitment to the ductal and islet lineages. However, clonal tracing studies spanning the entire developmental time window remain unaddressed.

The interpretation of static lineage tracing data as a reflection of cell potency and histogenesis is further confounded by the large-scale cell rearrangements that take place during pancreatic morphogenesis. Combining genome-wide transcription factor expression analysis with clonal tracing studies, Zhou et al. have argued that the first phase of ductal branching is controlled by multipotent Pdx1^{+}Pft1a^{+}cMyc^{High}Cpa1^{+} progenitors located within tip domains that give rise to acinar cells and fate-restricted trunk cells that form the trailing ducts and islets (Zhou et al., 2007). However, the question of whether and how these progenitors act collectively to coordinate the prolonged phase of branching morphogenesis (Bankaitis et al., 2015) remains unclear: how does cell fate restriction during development correlate with changes in the proliferative potential of progenitors to achieve spatio-temporal patterning of tissue? To what extent do the mechanisms of fate specification and ductal patterning in pancreas mirror the morphogenesis of other branched organs such as the lung, mammary gland, or kidney (Iber and Menshykau, 2013)?

Here, by combining clonal lineage tracing using whole-mount reconstructions with proliferation kinetics, single-cell gene expression profiling, and quantitative biophysical modeling, we define both the lineage hierarchy and the spatio-temporal dynamics of pancreatic precursors during development. Based on the statistical properties of the complex branched ductal network, we use these insights to develop a theoretical basis to understand the large-scale organization of the pancreas. Finally, by drawing a quantitative comparison with the statistical organization of the mouse mammary gland ductal epithelium, we argue that the basic mechanism of ductal patterning is conserved across different tissues.

RESULTS

Clonal Lineage Tracing Provides Evidence for Early and Progressive Lineage Restriction

To study the lineage and proliferative potential of pancreatic precursors, previous studies have exploited transgenic mouse models based on targeted promoters (Zhou et al., 2007; Solar et al., 2009; Kopp et al., 2011; Kopinke et al., 2011; Pan et al., 2013). Here, to capture the dynamics of precursors in an unbiased manner, we made use of the *Rosa26*-CreERT2/*Rosa26*-Confetti mouse model to label all cell types in the developing pancreas (Figures 1B–1J and S1A–S1L), turning later to consider a cell-specific promoter. To monitor changes in the potency of precursors and the dynamics driving branching morphogenesis, clones were induced around the start of the two key stages of development, E9.5 and E12.5 (Figures 1A and 1B). However, due to the time delay in activating and clearing Tamoxifen (Nakamura et al., 2006; Gu et al., 2002), we note that cells could be induced as much as a day after injection. To achieve clonal density labeling, we titrated the Tamoxifen dose so that labeled cells were scarce (<3% by volume using Tamoxifen injection at 0.025 mg/g of pregnant female for E12.5 induction, Figures S1M–S1O; 0.020 mg/g for E9.5 induction). Tissue was then harvested at post-natal day (P) 14 and P28, and clones were reconstructed in 3D using thick serial sections (Figures 1C–1J, S1B–S1O, and Videos S1, S2, S3, and S4).

By exploiting the confetti labeling system to control for potential clone merger and fragmentation events, we used a rigorous assessment to identify clones with defined statistical confidence (Figure S2 and STAR Methods). Additionally, we verified that our assessments of potency were independent of the color quantified, even though there was a nearly 10-fold difference in the GFP (green fluorescent protein) and RFP (red fluorescent protein) induction frequency. Clones were quantified with respect to position, size (by volume), and cell composition based on chromogranin A (islet), Dolichos biflorus agglutinin (DBA, ductal), and DAPI (nuclear staining, 4′,6-diamidino-2-phenylindole) staining (Figures 1C–1J and S1; STAR Methods). Throughout, our assessments of cell composition were obtained from the analysis of high-resolution images. Amylase staining confirmed that nearly all non-ductal and non-islet cells of the dissected pancreas at the stages analyzed were of acinar type (Figures S3 and S4A–S4C). Ductal and islet lineages could also be clearly distinguished morphologically when stained in a single channel, based on control stainings (Figures S4D–S4F). To further verify the integrity of lineage assignments and exclude the possibility

Figure 1. Quantitative Lineage Tracing Reveals Evidence of Early Lineage Commitment of Heterogeneous Pancreatic Precursors
(A) Schematic depicting key stages in pancreas development.
(B) Schedule of lineage tracing experiments using the *Rosa26*-CreERT2/*Rosa26*-Confetti mice.
(C) Thick 100-μm section of P14 pancreas induced at E9.5.
(D) Zoom on tripotent RFP clone from (C), containing islet, acinar, and ductal components (indicated by an arrowhead, asterisk, and arrow, respectively) with zoom on single Z-sections demonstrating the contribution of clones to different pancreatic compartments.
(E) Thick 100-μm section of P14 pancreas induced at E12.5 reveals heterogeneous clonal outcomes of various potencies.
(F) Acinar majority nGFP clone from (E) (acinar compartment indicated with an asterisk) surrounding a network of ducts with rare labeled ductal cells (indicated by arrow) and shown in zoom on single Z-section. High-resolution images were used in (D)–(F) for the assessment of potency.
(G) Low-resolution image of a small cYFP clone from (E) containing a few cells.
(H) Representative unipotent acinar-only cRFP clone from (E) surrounding unlabeled ducts. The lack of overlap between clone and ducts is indicated in single Z-section images on the right.
(I) Ductal-only mCFP clone from (E) containing monoclonal ducts (indicated by arrow) and ductal overlap shown on single Z-sections on the right.
(J) Bipotent cRFP clone from (E) in red (cRFP) (islet and ductal compartments indicated by an arrowhead and arrow, respectively).
(K) Global potency of clones induced at E9.5 and E12.5, indicating a shift toward unipotency.
(L) Fraction of unipotent clones versus induction probability for each confetti color. Bars indicate mean and SD. Nuclei stained with DAPI (blue), islets immunostained using chromogranin A antibody (white), and ducts stained with DBA (white). While 41 clones were counted from n = 3 mice for the E9.5 to P14 tracing, 254 clones were counted from n = 3 mice for the E12.5 to P14.
(M) Comparison of global potency between both quantification methods (Figures S2O and S2P) showed no statistically significant difference in the potency outcome (P = 0.37, Chi-square test; 53 clones from n = 4 mice). See also Figures S1–S6; Videos S1, S2, S3, and S4; and Table S1.

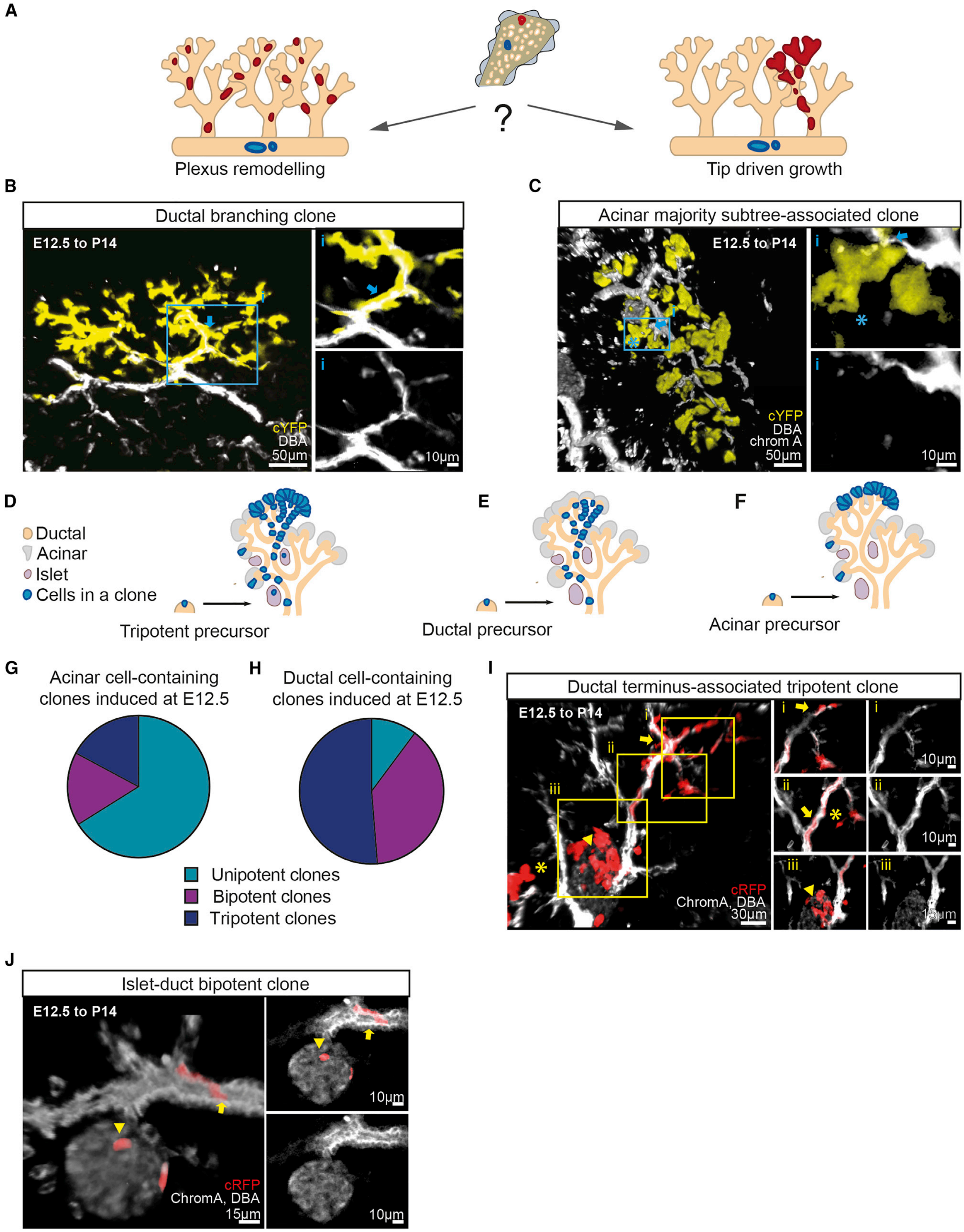

(legend on next page)

that clones arose from non-pancreatic lineages such as stroma, endothelium, or nerves, we confirmed our results quantitatively with an additional and independent clonal analysis using amylase, chromogranin A, and DBA co-immunostaining (Figure S3 and STAR Methods). Finally, we checked that the overall increase in average clone size of the acinar compartment (Figure S2L) tracked proportionately the increase of pancreas volume between E13.5, P14, and P28 (Figures S2M and S2N), confirming the representative nature of the clonal tracing assay and the integrity of clonal assignment.

From clonal reconstructions, two features were common to both early and late induction and collection times. First, clones remained remarkably cohesive in both ductal and acinar compartments (Figures 1C–1J, S1B–S1O; Videos S1, S2, S3, and S4), indicating that despite large-scale cell rearrangements, the dispersion of proximate cells during the secondary transition between disconnected ductal branch structures must be limited; however, a degree of bidirectional dispersion of cells along a connected ductal branch could not be ruled out. Second, clones were observed over a wide range of sizes, with some containing thousands of cells spanning large areas of tissue, whereas others contained only a few cells; many clones displayed a branching morphology (Figures 1F, 1G, and S5A–S5C).

Based on cell composition, we identified uni-, bi-, and tripotent clones, with permutations across all three lineages (Figures 1D, 1F–1J, S5D–S5F, S2O, and S2P). Strikingly, examining the composition of clones traced from E12.5 to P14 and P28, we found that the majority were unipotent, based on chromogranin A, DBA, and amylase stainings (68% at P14 and 72% at P28; Figures 1K–1M, S5D, and S5E). (For a complete list of clone types and sizes, see Table S1 and Figures S2O, S2P, S5D, and S5F.) This argues that the lineage potential of most precursors has already become restricted by E12.5, contrasting with previous findings (Zhou et al., 2007; Solar et al., 2009; Kopp et al., 2011; Furuyama et al., 2011). Indeed, these results suggest that reports of extensive tripotency as late as E12.5–14.5 may be an artefact of high-density labeling, tracing the evolution of populations instead of individual cells. Examination of the clonal composition from the E9.5 to P14 tracing (Figures 1K and S5F) performed under the same conditions revealed that the majority of clones remained bi- or tripotent (64%), as expected from the lack of tip-trunk segregation at this early stage of development (Pan and Wright, 2011). Yet, even at this time point, some 25% of induced cells generated unipotent clones (see Discussion).

Could heterogeneity in potency explain the wide variability of clone sizes, with multipotent progenitors giving rise to large clones and unipotent progenitors generating small clones? Although multipotent clones were, on average, slightly larger than unipotent clones, each category still contained clones spanning three orders of magnitude in size (Figure S5B), suggesting that the observed heterogeneity does not derive from the labeling of distinct progenitor types within a developmental hierarchy. Therefore, to understand the source of clone size heterogeneity and gain insight into the dynamics and large-scale patterning of tissue during the secondary transition, we examined the location and spatial organization of clones.

Branching Morphogenesis Is Driven by Both Multipotent and Fate-Restricted Precursors Localized at Ductal Termini

We first considered whether the specification of the pancreas relies on an early phase of expansion that precedes plexus remodeling or whether the bulk of expansion takes place through the later phase of ductal branching once the plexus is formed. We reasoned that if clonal expansion occurs mainly before plexus remodeling, clones would become fragmented and dispersed across adjacent ductal subtrees. If, on the other hand, ductal expansion is derived from the activity of precursors localized to side-branches of the plexus, clones induced at E12.5 would track segments of individual ductal subtrees (see Figure 2A for a schematic, where subtrees are identified as the connected networks of branches and sub-branches emanating from central ducts.). Notably, we found that the vast majority of ducts that contained labeled cells (ca. 80%) were characterized by a high degree of monoclonality (Figure 2B) and that these clonally labeled ducts were restricted to the same subtree, generally extending to the periphery of the ductal network (Figures 2B and 1I). Remarkably, we also found that the majority (ca. 90%) of acinar cell-containing clones, including unipotent (acinar-only) clones, were closely associated with single ductal subtrees across multiple generations of consecutive branching events (Figures 2C, 1F, and 1H), indicating a tight correlation between the branching program and the expansion of the acinar compartment. Importantly, ductal and acinar clones at the periphery of the pancreas were consistently larger and more branched than clones in the center ($p < 0.01$, Mann-Whitney test, Figure S5C). Together, these results suggest that during the secondary transition phase, pancreas development follows a coordinated process of branching morphogenesis, driven by precursors at, or near, the termini of ducts that self-renew through serial rounds of branching, giving rise to proliferative cells that form the trailing ducts as well as acinar cells that associate with the ends of terminated ducts.

Figure 2. Morphology and Potency of Clones Reveal Evidence for Ductal End-Driven Branching Morphogenesis

(A) Schematic depicting the different types of clones that would be expected to arise during pancreatic development being driven either solely by plexus remodeling or by ductal end-driven branching morphogenesis.

(B) Ductal branching clone, monoclonally labeling the distal parts of ducts with zoomed-in single Z-section on the right showing the overlap of DBA with cYFP. Ducts indicated with an arrow.

(C) Ductal terminus-associated acinar clone with acinar cells (asterisk) closely surrounding ducts stained with DBA (white, arrow), zoomed-in single Z-section on the right showing a very small overlap of DBA with cYFP.

(D–F) Schematics demonstrating the distinct clone morphologies arising from the labeling of different types of ductal-end precursors, with (D) depicting a tripotent clone, (E) a unipotent ductal clone, and (F) a unipotent acinar clone.

(G and H) Potency of (G) acinar cell- and (H) ductal cell-containing clones induced at E12.5. Ductal cells remain bi- and tripotent, whereas acinar precursor clones become increasingly lineage-committed.

(I and J) Ductal-terminus associated tripotent cRFP (I) and bipotent cRFP (J) clones. Islet, ductal, and acinar compartments indicated by an arrowhead, arrow, and asterisk, respectively. See also Figure S1 and Table S1.

We then assessed the fate potential of self-renewing precursors at the ductal termini. Concentrating on the E12.5 induction, we reasoned that if branching morphogenesis is driven by multipotent progenitors, clones tracking whole subtrees should contain a mixture of acinar and ductal cells (see schematic in Figure 2D). If, on the other hand, branching morphogenesis is driven by the coordinated dynamics of fate-restricted progenitors as occurs, for example, during the pubertal development of mouse mammary gland (Scheele et al., 2017), clones tracking whole ductal subtrees should be of either purely acinar or ductal type (Figures 2E and 2F). In support of the second hypothesis, some 70% of acinar-containing clones induced at E12.5 were found to be unipotent (Figure 2G, as well as Figure S5G for E9.5 induction). The fact that these clones often spanned multiple rounds of consecutive branching of the same ductal subtree suggests that acinar cells arise from acinar-committed precursors that associate with ductal termini and undergo renewal during ductal bifurcation (Figure 2F). By contrast, the vast majority of ductal cell-containing clones traced from E12.5 were found to be multipotent (Figure 2H, as well as Figure S5H for E9.5 induction) and included tripotent clones (Figures 2I and S1J), bipotent ductal-acinar clones with typically sparsely labeled ductal cells (Figures 1F and S1G–S1I), as well as some bipotent ductal-islet clones (Figure 2J). Altogether, these findings suggest that at E12.5, growing ductal termini host both multipotent progenitors and fate-restricted acinar and ductal progenitors, which act cooperatively to drive pancreatic growth.

While clonal tracings based on a ubiquitous promoter allow for an unbiased assessment of lineage potential, it does not reveal whether the potency of cells correlates with defined molecular signatures. Therefore, to consolidate our findings, we turned to a targeted lineage tracing strategy using a *Sox9*-CreERT2 mouse model (Kopp et al., 2011). Although Sox9 expression has been associated with tripotency (Kopp et al., 2011; Furuyama et al., 2011), an examination of clones induced at E12.5 at low induction frequency (Tamoxifen injection at 0.025 mg/g of pregnant female) revealed morphologies and potencies qualitatively similar to that found using the *Rosa26* model (compare Figures 3A, 3B, S5K–S5O with Figures 2B and 2C), identifying tree shaped clones (Figures S5K–S5O), with a slight majority of individual *Sox9*-targeted cells already fate-restricted to either the acinar or ductal-islet lineage. As expected, when compared to the *Rosa26* tracing, we noticed an enrichment of multipotent clones (Figures S5P–S5R, $p < 0.0001$, chi-square test) and ductal cell-containing clones (Figure S5S, $p < 0.0001$, chi-square test), arguing that *Sox9* targets a heterogeneous cell population biased toward the ductal lineage. As well as supporting the representative character of the Rosa26 tracings, these findings further emphasize the importance of using a clonal assessment of cell fate potential.

We then investigated whether the fate-restriction of pancreatic precursors was accompanied by a decreased proliferative potential. Strikingly, we found that although there were differences between the average size of unipotent clones and that of bi- or tripotent clones (for both the ductal and acinar compartments), these differences were small (2-fold maximum) compared to the large clone size heterogeneity (spanning four orders of magnitude) (Figures 3C and 3D). This shows that self-renewing fate-restricted acinar and ductal precursors retained a growth potential similar to that of multipotent self-renewing progenitors. This observation implies that the former must be specified early in development (during tip-trunk segregation) and are replenished by the latter only rarely during the later stages of development.

In summary, the potency and spatial organization of clones are suggestive of a cellular hierarchy in which, during the secondary transition, minority populations of both multipotent and fate-restricted self-renewing precursors localized to growing ductal termini act in concert to coordinate a process of branching morphogenesis. This gives rise to ductal progenitors that locally expand the maturing ducts, as well as to delaminating islet precursors (Figure 3E).

Correlation of the Distribution of Clone and Ductal Subtree Sizes Suggests that They Share a Common Origin

To understand the origin of clone-size heterogeneity, we turned to a quantitative analysis of the clone size distributions (Figure S6). Comparison of ductal and acinar cell-containing clones showed that the size distributions of the respective compartments, rescaled by the average clone size, was strikingly similar both between lineages at P14 (Figures S6A–S6C, $p = 0.68$, Mann-Whitney test), as well as within the same lineage between the P14 and P28 collection time points (Figure S6D, $p = 0.63$ for acinar and $p = 0.95$ for ductal, Mann-Whitney tests; STAR Methods). That is, while their average sizes differ by more than an order of magnitude (E12.5 to P14 tracing), the chance of finding a clone larger than some multiple of the average remains the same for both the acinar and ductal lineage; and the same was true for E9.5-traced clones (Figures S6E–S6H). This suggests that the gross heterogeneity in clone sizes of the acinar and ductal compartments may share a common origin in the chance collective fate decisions of self-renewing progenitors during the phase of branching morphogenesis.

To challenge this hypothesis, we assessed the emergence and degree of gross heterogeneity of the growing ductal network itself. In line with previous reports (Puri and Hebrok, 2007; Kesavan et al., 2009; Villasenor et al., 2010; Bankaitis et al., 2015; Shih et al., 2016), an analysis of whole-mount tissue at E13.5 and E15.5 revealed a transition from a plexus structure to central ducts from which multiple smaller ducts start to branch (Figures 3F and 3G). By E18.5, whole-pancreas ductal reconstructions revealed a strikingly complex and intricate ductal network, reflecting multiple (>10) rounds of serial branching events tracking back to the central duct (Figure 3H), consistent with a phase of branching morphogenesis. Notably, focusing on measurements made from whole-mounts at E18.5, we found that some ductal subtrees remain small and localized to the center of the pancreas and comprise only a limited number of branch segments (Figure 4A, arrow). By contrast, other subtrees expanded radially, reaching out to the periphery of the organ and colonizing a large volume of tissue (Figure 4A, arrowhead). We then quantified the raw distribution of subtree sizes, defined as the total number of branches (i.e., segments between two bifurcation points) within each subtree emanating from the central ducts (Figures 4B and 4C). Strikingly, when compared to the clonal data, we found that the rescaled subtree size distribution matched closely the rescaled clone size distributions of both the ductal and acinar compartments (Figure 4D), suggesting that clone size

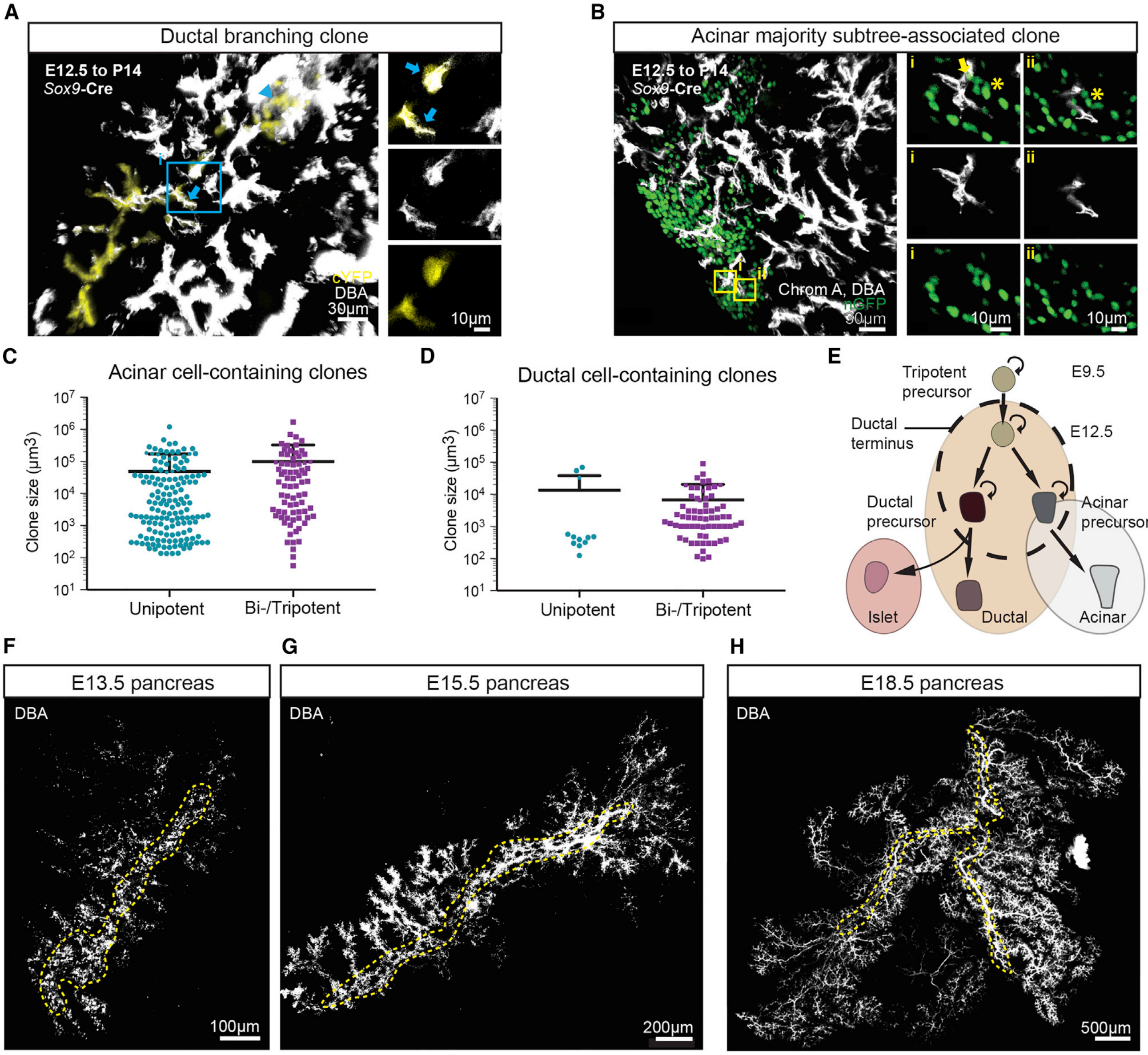

Figure 3. Establishing the Hierarchy of Progenitor Cells in the Pancreas

(A and B) *Sox9*-CreERT2; *Rosa26*-Confetti lineage tracing recapitulates the basic morphology and cell type composition of clones in Figures 2B and 2C with both (A) branched ductal clones and (B) acinar subtree-associated clones present in abundance, supporting the hypothesis that self-renewing ductal and acinar precursors at ductal termini drive pancreatic branching morphogenesis. Ductal and acinar compartments are indicated by an arrow and asterisk, respectively.

(C and D) Comparison of sub-clone sizes in the acinar (C) and ductal (D) compartment for unipotent versus bi- or tripotent clones. Bars indicate mean and SD.

(E) Hierarchy of multipotent precursors and their lineage-restricted progeny inferred from lineage tracing data.

(F–H) Evolution of pancreatic ductal network stained in whole-mount with Dolichos biflorus agglutinin (DBA) at (F) E13.5, (G) E15.5, and (H) E18.5. Central plexus indicated in outline by dashed blue lines. See also Figure S5 and Table S1.

heterogeneity indeed derives from the stochasticity of the branching process.

Dynamics of Branching Morphogenesis

Altogether, these findings suggest that during the phase of branching morphogenesis, clonal dynamics may be more usefully understood at the ‘‘mesoscopic level,’’ thereby focusing on the organizing principles directing the collective cell dynamics of the ducts rather than the individual fate decisions of their constituent cells. To proceed, we therefore sought to define a minimal model that captured the dynamics of the ductal branching process. To this end, we noted that morphogenesis takes place in a largely two-dimensional setting (Figures S2L–S2N), with ductal branches showing remarkably few crossovers above and below each other (Figure 4A). Moreover, ductal subtrees with small numbers of branches extended very little spatially and often terminated abruptly when ‘‘shadowed’’ by much larger and expanding subtrees. Based on these observations, we

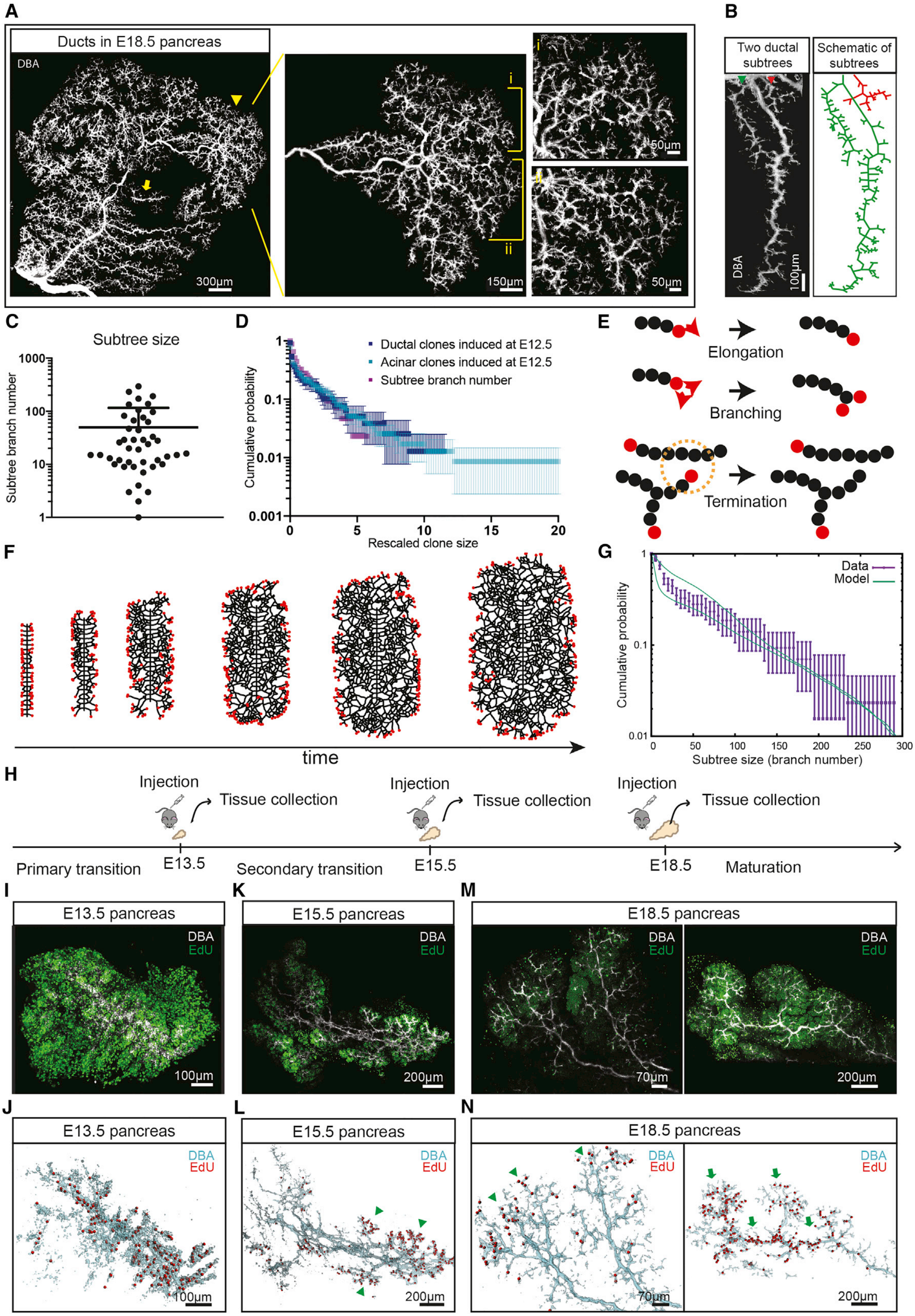

(legend on next page)

proposed a quantitative model where equipotent ductal termini, initiating as side-branches from a few central, plexus-derived ducts specified early in development, drive a process of stochastic ductal branching and elongation, which terminates when termini move into proximity of neighboring ducts. Specifically, we asked whether this model, whose sole key parameter is the measured average separation of independent adjacent subtrees along the central duct, could recapitulate quantitatively the observed subtree growth heterogeneity, as has been shown previously for mouse mammary gland and kidney (Hannezo et al., 2017), without the need to invoke the existence of engrained cellular heterogeneity (see STAR Methods for details).

Notably, numerical simulations of the model showed that, consistent with experiment, (1) active ductal termini "self-organize" during development into a traveling pulse at the periphery of the expanding ductal network, while central regions are formed at a roughly constant density (Figures 4E and 4F); and (2) some subtrees become rapidly overshadowed by neighbors, terminating early, while others expand to host hundreds of branch segments formed over multiple generations of branching. Strikingly, a comparison of the subtree size distribution revealed a nearly perfect agreement between the model prediction and experiment (R^2 = 0.99, Figures 4G and S6I–S6K; STAR Methods). This shows that the statistical heterogeneity of pancreas subtree (as well as clone) sizes can be explained quantitatively by a simple paradigm in which all ductal termini have *a priori* the same growth potential, but their branching activity is terminated by arresting signals from neighboring ducts.

To probe the second prediction from the model, we studied proliferation within ducts, using short-term EdU incorporation (2-hr chase) and whole-mount imaging at E13.5, E15.5, and E18.5 (Figure 4H). At E13.5, we found a uniform pattern of proliferation (Figures 4I and 4J). However, at E15.5, ductal proliferation (and, to a lesser degree, acinar proliferation) was greater in peripheral regions of ductal subtrees, with an enrichment of activity at the ends of ducts (Figures 4K and 4L, arrowheads), consistent with ductal end-driven morphogenesis and the predictions of the model (Figure 4F). At E18.5, EdU showed a more heterogeneous pattern, with some parts of the pancreas characterized by enhanced proliferation at ductal termini (Figures 4M and 4N, arrowheads), while other regions were characterized by a more uniform low-level of proliferation (Figures 4M and 4N, arrows). Together, these results support the hypothesis that the early stages of branching morphogenesis (around E15.5) are fueled by self-renewing precursors positioned at ductal termini, which drive a process of ductal elongation and bifurcation while, at later stages, growth is dominated by the local expansion of ducts, as well as acini and islets.

Based on these insights, we then turned to consider the number of self-renewing precursors within a given ductal terminus. Since the ends of ducts appeared roughly constant in size throughout development and were frequently cleft-shaped (Bankaitis et al., 2015), we posited that ductal bifurcation segregates precursors approximately equally, after which they undergo a round of symmetric duplication to recover their original size. Using the inferred branching dynamics, we then simulated clonal dynamics based on the random segregation of labeled cells (Scheele et al., 2017). Chance segregation and expansion of clonally labeled precursors during ductal bifurcation allows the fraction of lineage-labeled cells in newly-formed ducts to "drift" in size, leading to a gradual process of "monoclonal conversion" in which, with increasing branch generation along the network, ducts eventually become either fully labeled by a single confetti color or completely unlabeled (Figure 5A). Importantly, the rate of monoclonal conversion along the ducts is predicted to scale in inverse proportion to the number of self-renewing ductal precursors contained in each terminus (Figures 5A, 5B, S6L, and S6M; STAR Methods).

An inspection of the experimental data showed that labeled subtrees are initially "mosaic" (both in the acinar and ductal compartments), containing both labeled and unlabeled cells but showing evidence of rapid monoclonal conversion along the ductal network so that, in many cases, more distal ends are entirely comprising confetti+ cells marked by a single color

Figure 4. Correlation of the Distribution of Clone and Ductal Subtree Sizes Suggests that They Share a Common Origin

(A) Branching structure of an E18.5 pancreas (ducts stained by DBA, white), with an arrow and arrowhead pointing to a small and large subtree, respectively, with insets i-ii highlighting the heterogeneity of branch and subtree sizes.

(B) Zoomed-in image of two pancreatic subtrees of E18.5 pancreas (left) and a sketch of the two branches used in subtree size quantification (right), demonstrating the heterogeneity of branching morphogenesis. Subtrees are identified as a duct and its daughter ducts diverging from the central duct.

(C) Subtree sizes (scored by the number of branches arising from a large central duct) determined from E18.5 pancreas. Bars indicate mean and SD.

(D) Cumulative size distribution of ductal and acinar clones induced at E12.5, overlaid with the cumulative subtree size distribution (number of branches) determined from E18.5 pancreas. Distributions were determined from sizes rescaled by the ensemble average size, e.g., the point at (3,0.1) indicates that some 10% of clones or subtrees have a size three times larger than the average, etc. Bars indicate mean and SD.

(E) Sketch of the three theoretical rules used to model pancreatic branching morphogenesis: active ductal termini (red) elongate and branch stochastically as a default state, giving rise to less proliferative ductal cells (black) that contribute to the local expansion of the trailing ductal network, whereas ducts irreversibly terminate when in proximity to other ducts.

(F) Left to right: snapshots from the model simulation, starting from 40 active ductal termini initiating as side branches from a central duct, which create a complex branching structure in a self-organized manner.

(G) Theoretical subtree size distribution based on 500 simulations, such as that shown in (F), compared to the experimental subtree distribution, showing quantitative agreement. Thin lines represent the 95% confidence interval. For details of the model implementation, see STAR Methods. Bars indicate mean and SD.

(H) Schedule of EdU incorporation experiments.

(I–N) Short-term (2-hr) EdU-incorporation shows proliferative activity of ductal and acinar cells. Top: whole-mount pancreatic ducts (stained for DBA, white; and EdU, green); bottom: ductal surface reconstruction (blue) with ductal EdU+ cells marked in red. At E13.5 (I and J), proliferation is evenly distributed throughout the ducts (n = 3 mice) whereas, at E15.5 (K and L), proliferation occurs predominantly at the periphery of the pancreas in the tip regions of ducts (n = 3 mice, arrowheads). At E18.5 (M and N), proliferation patterns become heterogeneous with some regions displaying ductal end-enrichment (n = 4 mice, arrowheads, left), while others proliferate more homogeneously (n = 5 mice, arrows, right). See also Figure S6 and Table S1.

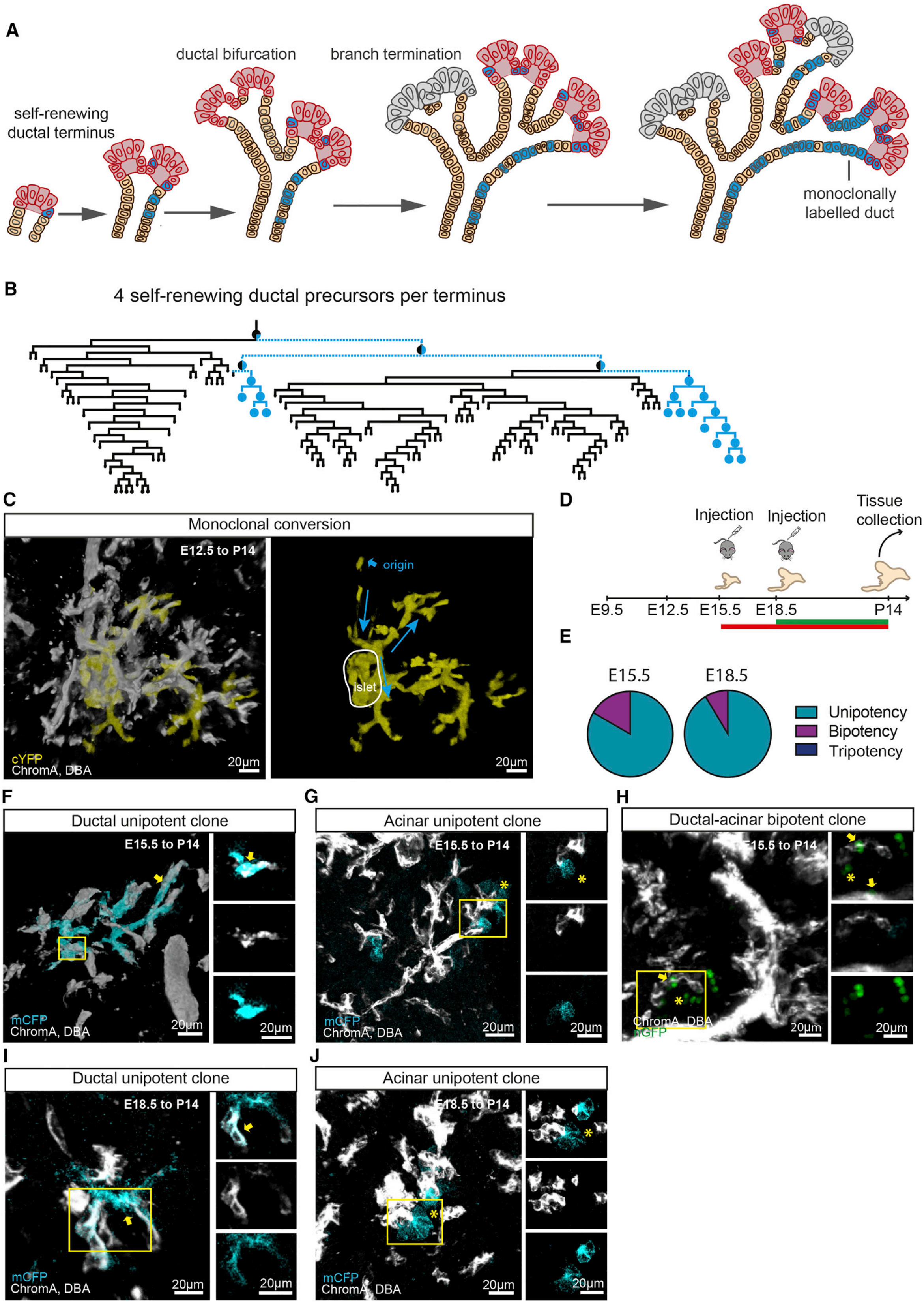

(legend on next page)

(Figures 2B, 2C, and S1J–S1L). Importantly, the clonal data suggested that in contrast to the large number of fate-restricted "stem cells" hosted in each active terminal end-bud of the mammary gland epithelium (Scheele et al., 2017), pancreatic branching morphogenesis is reliant on ductal termini hosting as a few as 4–6 self-renewing ductal precursors (Figures 5B and 5C; STAR Methods). Note that here we apply a functional definition of self-renewing progenitors as cells that contribute long-term to the development of a subtree via branching morphogenesis. Although we cannot rule out the possibility that self-renewing progenitors shuttle back and forth between ductal termini and the proximate branch, as observed during kidney morphogenesis (Packard et al., 2013), to ensure that they are appropriately replicated during ductal bifurcation, it seems most likely that self-renewing progenitors lie clustered at the distal end of the terminus, which constitutes a localized niche.

Finally, to better characterize the temporal dynamics of branching morphogenesis, we analyzed clones induced at other developmental time points (Figure 5D), which revealed that branching was far less prevalent at later stages of development (Figures 5E–5J, S5I, and S5J). Tracing from E15.5 to P14, we observed smaller clones that still tracked subtrees over several rounds of consecutive branching events, albeit less frequently and forming smaller trees than at E12.5 ($p = 0.01$, Mann-Whitney test, Figures S5I and S5J). These clones were either of unipotent ductal (Figure 5F) or acinar (Figure 5G) composition or, more rarely, of bipotent ductal-acinar or islet-ductal composition (Figures 5E, 5H and S6N). Tracing from E18.5 to P14, we found that clones displayed minimal but non-vanishing (ductal-acinar) bipotency and were even less branched (Figures 5E, 5I, 5J, S5I, S5J, and S6O), consistent with branching morphogenesis being superseded by a process of expansion via local dilation of an existing tree structure.

Molecular Signature of Pancreatic Precursors Revealed by Single-Cell RNA Sequencing

To investigate the molecular basis of the observed functional heterogeneity of pancreatic precursors, we turned to single-cell RNA-seq, focusing on E13.25 (as a matching time point for the lineage tracing analysis, given the predicted time delay in the expression of Cre [Danielian et al., 1998]) and E15.25 to investigate whether and how the molecular signature of progenitors evolves during development (Figures 6 and S7). Combining data from E13.25 and E15.25 embryos, we first performed dimensionality reduction by principal-component analysis (PCA). This identified two distinct clusters of cells. Based on the complementary expression of *Epcam* and *Vimentin*, one of these clusters was identified as mesenchyme, while the remaining cluster was pancreatic (Figures 6A and S7A). Filtering for pancreatic cells, we performed dimensionality reduction by t-distributed stochastic neighbor embedding (t-SNE), which groups together cells of similar gene expression (Van Der Maaten et al., 2008), followed by k-means clustering of 303 cells. This identified 4 distinct clusters of cells corresponding to an early endocrine-committed cluster (based on *Neurog3* expression) (Figures 6B and 6D, upper-left), an early (E13.25) and late (E15.25) acinar cluster (*Ptf1a, Cpa1, Myc*) (Figures 6B upper right, and 6D; Figures S7B and S7C), and a cluster expressing elevated trunk or ductal markers (*Sox9, Hes1, Nkx6-1*) (Figures 6B lower left, and 6D; Figures S7D and S7E). This pattern of expression was consistent with early lineage commitment of pancreatic cells, as observed from the lineage tracing data.

We then quantified the degree of gene expression variability within individual clusters (Figure S7I–S7L) (Lun et al., 2016, see STAR Methods). This analysis showed that for the ductal cluster, *Sox9 and Cpa1* were heterogeneously expressed (Figure S7I), while *Sox9* was homogeneously expressed in acinar cells (Figures S7K and S7L). *Ngn3* was expressed heterogeneously within the islet cluster (Figure S7J), as previously reported (Bankaitis et al., 2015), while *Ptf1a* showed homogenous expression in the E15.25 acinar cluster (Figure S7L). Notably, the pan-proliferation *MKi67* marker showed significant variation, with the ductal cluster separating into two subclusters of high and low *MKi67* expression (Figure 6B lower-right corner and S7I).

To resolve the regional basis of molecular heterogeneity, we examined high-resolution images of tissue immunostained for Ki67, Sox9, Cpa1, and Ptf1a. This analysis showed that within ductal termini, both Cpa1 and Sox9 expression levels were elevated, usually reciprocally expressed, and spatially heterogeneous at the cellular scale, with the majority of these cells high in Ki67 (Figure 6E). These observations were consistent with proliferative acinar lineage-restricted and ductal or islet-restricted self-renewing precursors becoming localized at ductal termini during branching morphogenesis, as suggested by the lineage tracing analysis. Furthermore, we distinguished double positive $Cpa1^{high}/Sox9^{high}$ cells (Figure 6E, yellow arrows), which could constitute the presumptive rare multipotent cells, as suggested by the lineage tracing analysis. Consistent with the literature, within the trunk areas, we also observed $Sox9^{high}$ cells with reduced levels of Ki67 expression (Figure 6E, white arrows).

To study proliferative heterogeneity, we examined immunostainings of Ki67, Cpa1, and E-cadherin. At E15.25, areas of tissue

Figure 5. Branching Morphogenesis in the Ductal Termini Is Driven by a Small Number of Self-Renewing Progenitors

(A) Schematic depicting the process of monoclonal conversion.

(B) Model simulation of a subtree containing 4 self-renewing ductal precursors per ductal terminus with one initially labeled precursor cell (for details, see STAR Methods).

(C) Representative ductal cYFP clone showing monoclonal conversion; the direction of ductal growth is indicated with an arrow. Comparison with simulations, (B) and Figures S1J–S1L, suggest that growing ductal termini are composed of as few as 4 self-renewing ductal precursors.

(D) Outline of experiment with induction at E15.5 and E18.5.

(E) Clones induced at E15.5 and E18.5 were mainly unipotent.

(F–H) P14 pancreas induced at E15.5 showing only a few rounds of branching events in (F) ductal and (G) acinar compartment. In addition to unipotent clones as in (E) and (F), bipotent ductal-acinar clones were also observed (H).

(I–J) P14 pancreas induced at E18.5, revealing only a single bifurcation event within traced clones and unipotency in the (I) ductal and (J) acinar compartments. Ductal and acinar compartments indicated by an arrow and asterisk, respectively. 78 clones were counted from n = 2 mice for E15.5 to P14 tracing, and 61 clones were counted from n = 2 mice for E18.5 to P14 tracing. See also Figure S6 and Table S1.

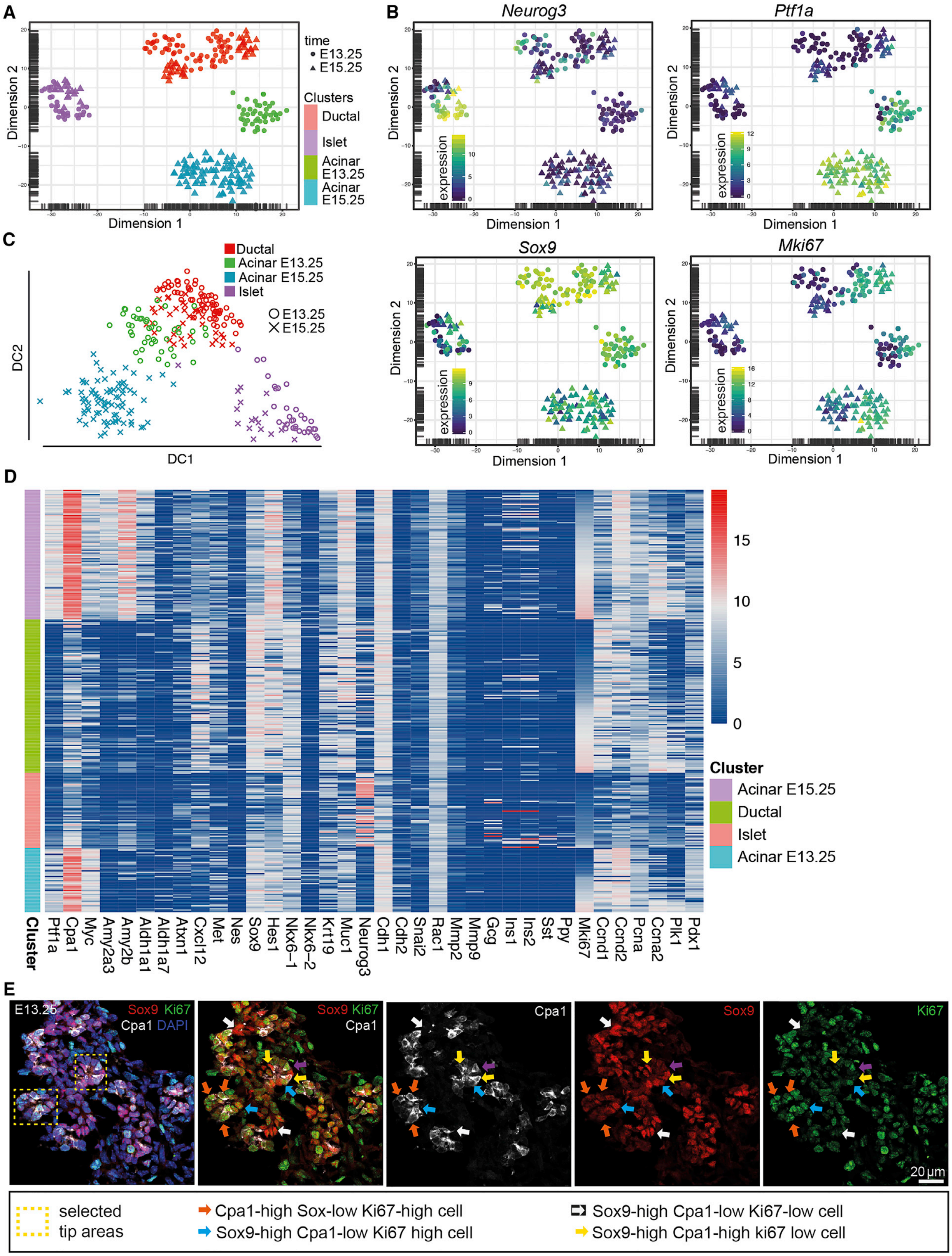

(legend on next page)

rich in Cpa1+ cells showed elevated levels of Ki67 expression, consistent with the single-cell sequencing data and the literature (Figures S7I, S7M, and S7N) (Zhou et al., 2007). With respect to E-cadherin+Cpa1− branches, we saw a modest degree of enrichment of Ki67^{high} cells at the more peripheral areas of the pancreas at E15.25 (Figure S7M), consistent with the branching morphogenesis program. We also observed heterogeneity of Ngn3 expression, as previously reported, and consistent with the single-cell sequencing data (Bankaitis et al., 2015) (Figures S7Q and S7R). There were no significant variations in Ptf1a expression for both time points within the Cpa1^{high} termini (Figures S7O and S7P).

To further dissect the lineage relationship between the acinar, ductal, and islet cells (Haghverdi et al., 2016) at both E13.25 and E15.25, we employed diffusion pseudo-time (DPT) ordering of the single-cell RNA-seq data. The results were consistent with ductal cells standing at the apex of the lineage hierarchy (Figure 6C). Consistent with the tracing data, we found *Sox9* expression in both the ductal and acinar cluster, becoming progressively down-regulated in opposition to the up-regulation of *Cpa1* ($p < 5.5\ 10^{-12}$ for both, Figures S7F and S7G). Interestingly, *MKi67*-high cells in the ductal cluster plotted on DPT segregate toward the acinar branch, whereas *MKi67*-low cells segregate toward the islet branch (Figure S7H), consistent with ductal- and acinar-committed progenitors residing in the proliferative ductal terminus and islet cells deriving from less proliferative trailing ducts (Figure 3E). Further segregation of the acinar clusters between E13.25 and E15.25 was consistent with maturation, signaled by the enhanced expression of *amylase* and *Ptf1a*, as well *Aldh1* and *Hes1* (Figure 6D).

DISCUSSION

Previous studies have resolved the major events in pancreas development from the specification of the central duct, plexus formation, and early side-branching (Puri and Hebrok, 2007) to the later stages of ductal expansion (Villasenor et al., 2010) and the mechanisms of differentiation into the endocrine and exocrine lineages (Zhou et al., 2007; Johansson et al., 2007; Solar et al., 2009; Schaffer et al., 2010; Kopp et al., 2011; Kopinke et al., 2011; Pan et al., 2013; Desgraz and Herrera, 2009). However, the functional basis of pancreatic development at the cellular level—ductal branching and acinar proliferation—have remained in question (Shih et al., 2013). Using a quantitative lineage tracing strategy, we have used 3D clonal reconstructions, statistical analysis, and biophysical modeling to define the cellular basis of pancreas morphogenesis.

Our results show that during the secondary transition, the expansion of the ductal network from a central plexus is driven by minority subpopulations of self-renewing precursors localized at active ductal termini (Figure 7). Through continuous rounds of ductal bifurcation, these distinct precursor populations are segregated between newly formed ductal termini and expanded, leading to rapid monoclonal conversion. After E12.5, the bulk of the expansion of the acinar compartment is brought about by self-renewing acinar-committed precursors. Based on the correlation of the size distribution and spatial organization of ductal and acinar-only clones, it follows that acinar-committed precursors must be co-localized with, and undergo the same dynamics as, self-renewing ductal precursors during bifurcation, in a manner reminiscent of the behavior of lineage-restricted basal and luminal stem cells during the postnatal development of the mouse mammary gland epithelium. However, in contrast to the overwhelming unipotency of acinar precursors, at E12.5, most self-renewing ductal precursors still retain multipotency. This hypothesis is reinforced by single-cell RNA-seq and complements the findings of Zhou et al. (Zhou et al., 2007) on how multipotent ductal precursors guide early organogenesis. In particular, these findings show that during the later phase of organogenesis, the growth potential of cells residing at ductal termini, and thus their contribution to branching morphogenesis, is not correlated with their lineage potential.

Lately, a similar clonal lineage tracing strategy was used to address the earliest phase of pancreas development, tracing clonal dynamics from E9.5 to E14.5 (Larsen et al., 2017). Although the findings of Larsen et al. are in general qualitative agreement with the current study, the relative abundance of individual clone types contrasts with the present findings. Several factors may contribute to this apparent discrepancy: first, since Larsen's study made use of a 4OH-Tamoxifen administration protocol, the actual induction time may differ by as much as a day between the two studies. Second, to assess potency, Larsen et al. make use of markers that are lineage-unspecific at E14.5. In particular, previous studies have shown that at E14.5 and until birth, cells positive for the ductal marker Sox9 may give rise to all the pancreatic lineages (Kopp et al., 2011; Solar et al, 2009; Furuyama et al., 2011). Similarly, Cpa1, used by Larsen et al. as a marker of mature acinar fate, has been shown to mark multipotent progenitors at the lineage level at least until E14 (Zhou et al., 2007). Finally, it has been shown that a fraction of Ngn3+ endocrine-associated precursors may revert to ductal and acinar fate (Wang et al., 2010; Beucher et al., 2012). Therefore, with an early collection time point, some labeled cells may be unresolved into lineages, whereas others may have already

Figure 6. Single-Cell RNA Sequencing Analysis Suggests Early Fate Restriction and Ductal Origin of Progenitors

(A and B) (A) t-SNE plot obtained from combining 516 single cells from pancreas obtained from n = 7 mice (n = 5 embryos at E13.25 and n = 2 embryos at E15.25) showing ductal, islet and early (E13.25) and late (E15.25) acinar clusters, as identified by the expression of marker genes shown in (B), *Neurog3* (upper left), *Ptf1a* (upper right and Figures S7B and S7C), and *Sox9* (lower left and Figures S7D and S7E). Lower right of (B) shows *MKi67* proliferation marker expression, showing evidence of segregation of the ductal cluster. Cell color indicates log transformed normalised read counts. Note that clusters of islet and ductal cells overlap at the two time points.

(C) Diffusion pseudo-time plot reveals evidence of lineage segregation with ductal cells at the apex of a hierarchy that branches separately into acinar and islet lineages.

(D) Expression levels of pancreas genes for individual cells at E13.25 and E15.25.

(E) 7-μm-thick pancreatic section immunostained for Sox9, Cpa1, and Ki67 expression reveals molecular heterogeneity within ductal termini, with Ki67+ cells showing reciprocal expression of Sox9 and Cpa1 (blue and red arrows) as well as some cells positive for both markers (yellow arrows), and Sox9+ Ki67-high cells in the trunk areas (white arrows). See also Figure S7.

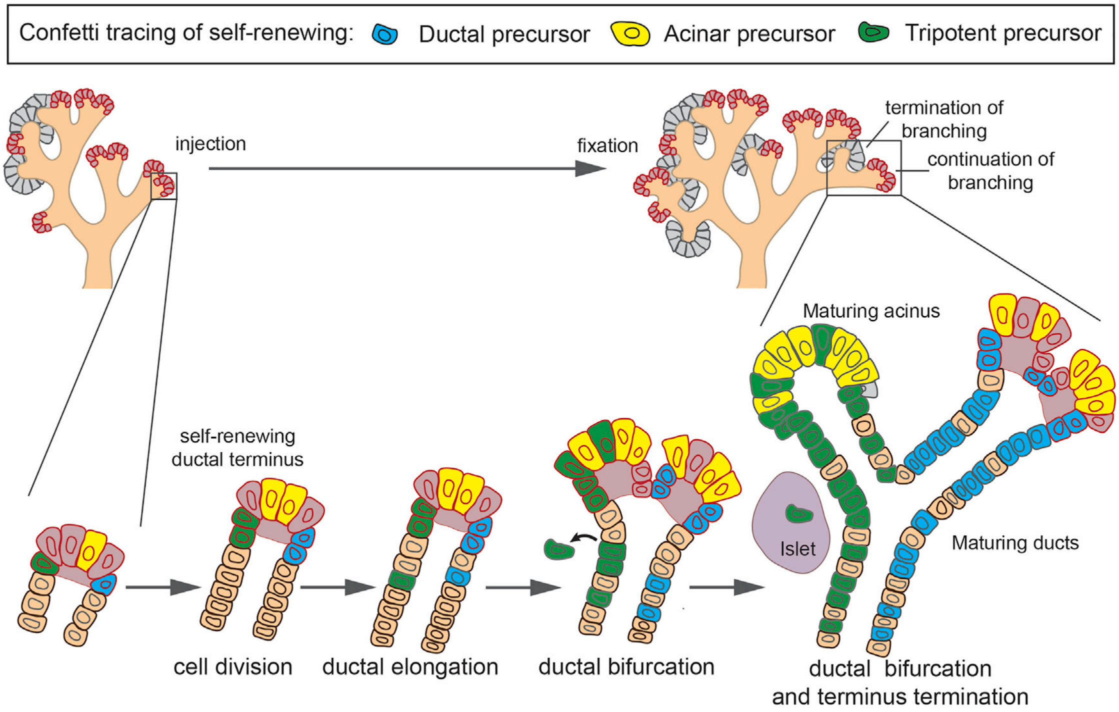

Figure 7. Summary Schematics Depicting Morphogenic Events during the Second Phase of Pancreatic Development

Pancreas morphogenesis follows from a process of stochastic ductal bifurcation and termination driven by self-renewing fate-restricted ductal and acinar precursors, as well as rarer tripotent precursors, which become depleted after E15.5. The figure presents the potential evolution of a single labeled mCFP+ ductal precursor, cYFP+ acinar precursor, and cRFP+ tripotent precursor. The pink area denotes proliferatively active cells in ductal termini.

committed to a definitive fate outcome but not yet up-regulated their corresponding lineage markers.

In summary, we propose that the large-scale organization of the pancreas is coordinated by a process of ductal end-driven branching morphogenesis, which may continue in concert with central plexus remodeling (Bankaitis et al., 2015). In this paradigm, the proliferative potential of precursors is not specified early in development but emerges from chance collective fate decisions made during branching morphogenesis. The statistical properties of the pancreatic ductal network are predicted quantitatively by a model in which ductal termini function as distinct niche-like domains, self-renewing through stochastic dichotomous branching until they terminate when in proximity with neighboring ducts. These dynamics mirror closely the quantitative behavior resolved in the mouse mammary gland and kidney (Hannezo et al., 2017), providing evidence for a conserved paradigm of branching morphogenesis in different mammalian tissues. Based on the current findings, we conclude that the regulation of ductal branching and termination are not directed by autonomous cell-fate decisions but occur cooperatively at the level of ductal termini via negative growth signals. Interestingly, a related model based on similar short-range inhibition was used recently to explain branching patterns in pancreatic organoids (Dahl-Jensen et al., 2016; Dahl-Jensen and Grapin-Botton, 2017). How environmental and signaling factors regulate these collective cellular decisions to promote ductal branching and termination thus remains an interesting question for future studies, as does the spatio-temporal interplay between branching morphogenesis, central plexus remodeling, and islet formation (Bankaitis et al., 2015).

The capacity of precursors to act cooperatively to achieve long-term self-renewal while giving rise to more differentiated progeny suggests that these cells may rely on regulatory programs similar to those borne by adult stem cell populations. Indeed, the controlled duplication of the self-renewing pool following ductal bifurcation suggests that the regulation of precursor number may be linked to localized factors at ductal termini, which constitute a closed niche environment. Resolving these factors will form the focus of future investigations.

STAR★METHODS

Detailed methods are provided in the online version of this paper and include the following:

- KEY RESOURCES TABLE
- CONTACT FOR REAGENT AND RESOURCE SHARING
- EXPERIMENTAL MODEL AND SUBJECT DETAILS
- METHOD DETAILS
  - Induction of Lineage Tracing
  - EdU Administration
  - Tissue Preparation
  - Tissue Staining
  - Confocal Microscopy and Image Analysis
  - Cell Fate Assessment
  - Single-Cell RNA Sequencing

- QUANTIFICATION AND STATISTICAL ANALYSIS
  - Statistics Reporting
  - Computational Analysis of Sequence Reads
  - Statistical Analysis of the Clonal Data
- DATA AND SOFTWARE AVAILABILITY

SUPPLEMENTAL INFORMATION

Supplemental information includes seven figures, one table, and four videos and can be found with this article online at https://doi.org/10.1016/j.devcel.2018.06.028.

ACKNOWLEDGMENTS

The authors would like to thank Peter Humphreys for help with image capture, processing, and analysis; Evangelia Diamanti for mapping sequencing data and producing QC plots; Maike Paramor and Joaquin Martinez Herrera for single-cell RNA sequencing sample submission and advice; Chiara Cossetti for FACS sorting; Heather Large and Julie Soffe for animal maintenance; Kenneth Jones for help, support and advice with animal colony management, and Bertrand Blondeau for providing the Ptf1a antibody. This work was supported by the Wellcome Trust (grant numbers 098357/Z/12/Z, B.D.S.; 110326/Z/15/Z, E.H.; and 097922/Z/11/Z, B.G.), Clinical Research Infrastructure Single-cell Facility (MR/M008975/1, B.G.), the MRC (MR/K018329/1, A.P.), and the Rosetrees and Stoneygate Trusts. A.P., M.K.S., and S.N. are MRC funded. E.H. is funded by a Junior Research Fellowship from Trinity College, Cambridge, a Sir Henry Wellcome Fellowship from the Wellcome Trust, and the Bettencourt-Schueller Young Researcher Prize for support. M.H. is a Sir Henry Dale fellow and is jointly funded by the Wellcome Trust and Royal Society (104151/Z/14/Z). This project received support through core funding of the Wellcome Trust-Medical Research Council Cambridge Stem Cell Institute (097922/Z/11/Z).

AUTHOR CONTRIBUTIONS

B.D.S., A.P., M.K.S., and E.H. designed the experiments. M.K.S. performed all the experiments, and R.A. contributed to Figures 6 and S7. S.N. prepared cDNA libraries for single-cell RNA-seq, and B.G. guided the single-cell sequencing experiments. M.H. and C.J.H. contributed to Sox9-Cre lineage tracing experiments, and J.N. was involved in the optimization of *Rosa26*-lineage tracing experiments. E.H., S.R., and M.K.S. performed analyses. M.K.S. and E.H. made the figures. A.P. and B.D.S. supervised the study. All authors discussed results and participated in the preparation of the manuscript.

DECLARATION OF INTERESTS

The authors declare no competing interests.

Received: August 28, 2017
Revised: February 15, 2018
Accepted: June 29, 2018
Published: July 26, 2018

REFERENCES

Anders, S., Pyl, P.T., and Huber, W. (2015). HTSeq-a Python framework to work with high-throughput sequencing data. Bioinformatics *31*, 166–169.

Angerer, P., Haghverdi, L., Büttner, M., Theis, F.J., Marr, C., and Buettner, F. (2016). Destiny: diffusion maps for large-scale single-cell data in R. Bioinformatics *32*, 1241–1243.

Bankaitis, E.D., Bechard, M.E., and Wright, C.V.E. (2015). Feedback control of growth, differentiation, and morphogenesis of pancreatic endocrine progenitors in an epithelial plexus niche. Genes. Dev. *29*, 2203–2216.

Beucher, A., Martín, M., Spenle, C., Poulet, M., Collin, C., and Gradwohl, G. (2012). Competence of failed endocrine progenitors to give rise to acinar but not ductal cells is restricted to early pancreas development. Dev. Biol. *361*, 277–285.

Dahl-Jensen, S., and Grapin-Botton, A. (2017). The physics of organoids: a biophysical approach to understanding organogenesis. Development *144*, 946–951.

Dahl-Jensen, S.B., Figueiredo-Larsen, M., Grapin-Botton, A., and Sneppen, K. (2016). Short-range growth inhibitory signals from the epithelium can drive non-stereotypic branching in the pancreas. Phys. Biol. *13*, 016007.

Danielian, P.S., Muccino, D., Rowitch, D.H., Michael, S.K., and McMahon, A.P. (1998). Modification of gene activity in mouse embryos in utero by a tamoxifen-inducible form of Cre recombinase. Curr. Biol. *8*, 1323–1326.

Desgraz, R., and Herrera, P.L. (2009). Pancreatic neurogenin 3-expressing cells are unipotent islet precursors. Development *136*, 3567–3574.

Edlund, H. (2001). Developmental biology of the pancreas. Diabetes *50*, S5–S9.

Flicek, P., Amode, M.R., Barrell, D., Beal, K., Billis, K., Brent, S., Carvalho-Silva, D., Clapham, P., Coates, G., Fitzgerald, S., et al. (2014). Ensembl 2014. Nucleic Acids Res. *42*, D749–D755.

Furuyama, K., Kawaguchi, Y., Akiyama, H., Horiguchi, M., Kodama, S., Kuhara, T., Hosokawa, S., Elbahrawy, A., Soeda, T., Koizumi, M., et al. (2011). Continuous cell supply from a Sox9-expressing progenitor zone in adult liver, exocrine pancreas and intestine. Nat. Genet. *43*, 34–41.

Gu, G., Dubauskaite, J., and Melton, D.A. (2002). Direct evidence for the pancreatic lineage: NGN3+ cells are islet progenitors and are distinct from duct progenitors. Development *129*, 2447–2457.

Haghverdi, L., Büttner, M., Wolf, F.A., Buettner, F., and Theis, F.J. (2016). Diffusion pseudotime robustly reconstructs lineage branching. Nat. Methods *13*, 845–848.

Hannezo, E., Scheele, C.L.G.J., Moad, M., Drogo, N., Heer, R., Sampogna, R.V., van Rheenen, J., and Simons, B.D. (2017). A unifying theory of branching morphogenesis. Cell *171*, 242–255.e27.

Iber, D., and Menshykau, D. (2013). The control of branching morphogenesis. Open Biol. *3*, 130088.

Johansson, K.A., Dursun, U., Jordan, N., Gu, G., Beermann, F., Gradwohl, G., and Grapin-Botton, A. (2007). Temporal control of neurogenin3 activity in pancreas progenitors reveals competence windows for the generation of different endocrine cell types. Dev. Cell *12*, 457–465.

Kesavan, G., Sand, F.W., Greiner, T.U., Johansson, J.K., Kobberup, S., Wu, X., Brakebusch, C., and Semb, H. (2009). Cdc42-mediated tubulogenesis controls cell specification. Cell *139*, 791–801.

Kopinke, D., Brailsford, M., Shea, J.E., Leavitt, R., Scaife, C.L., and Murtaugh, L.C. (2011). Lineage tracing reveals the dynamic contribution of Hes1+ cells to the developing and adult pancreas. Development *138*, 431–441.

Kopp, J.L., Dubois, C.L., Schaffer, A.E., Hao, E., Shih, H.P., Seymour, P.A., Ma, J., and Sander, M. (2011). Sox9+ ductal cells are multipotent progenitors throughout development but do not produce new endocrine cells in the normal or injured adult pancreas. Development *138*, 653–665.

Krijthe, J.H. (2015). Rtsne: T-distributed stochastic neighbor embedding using a Barnes-hut implementation. R Package Version 0.10. http://CRAN.R-project.org/package=Rtsne.

Larsen, H.L., Martín-Coll, L., Nielsen, A.V., Wright, C.V.E., Trusina, A., Kim, Y.H., and Grapin-Botton, A. (2017). Stochastic priming and spatial cues orchestrate heterogeneous clonal contribution to mouse pancreas organogenesis. Nat. Comm. *8*, 605.

Livet, J., Weissman, T.A., Kang, H., Draft, R.W., Lu, J., Bennis, R.A., Sanes, J.R., and Lichtman, J.W. (2007). Transgenic strategies for combinatorial expression of fluorescent proteins in the nervous system. Nature *450*, 56–62.

Lun, A., and Bach, K. (2016) Scran: Methods for Single-Cell RNA-Seq Data Analysis. R Package Version 1.2.0.

Lun, A.T., McCarthy, D.J., and Marioni, J.C. (2016). A step-by-step workflow for low-level analysis of single-cell RNA-seq data with Bioconductor. F1000Res *5*, 2122.

McCarthy, D.J., Campbell, K.R., Lun, A.T.L., and Wills, Q.F. (2017). Scater: pre-processing, quality control, normalization and visualization of single-cell RNA-seq data in R. Bioinformatics *8*, 1179–1186.

Nakamura, E., Nguyen, M.T., and Mackem, S. (2006). Kinetics of tamoxifen-regulated Cre activity in mice using a cartilage-specific CreER(T) to assay temporal activity windows along the proximodistal limb skeleton. Dev. Dyn. *235*, 2603–2612.

Packard, A., Georgas, K., Michos, O., Riccio, P., Cebrian, C., Combes, A.N., Ju, A., Ferrer-Vaquer, A., Hadjantonakis, A.K., Zong, H., et al. (2013). Luminal mitosis drives epithelial cell dispersal within the branching ureteric bud. Dev Cell *27*, 319–330.

Pan, F.C., and Wright, C. (2011). Pancreas organogenesis: from bud to plexus to gland. Dev. Dyn. *240*, 530–565.

Pan, F.C., Bankaitis, E.D., Boyer, D., Xu, X., Van de Casteele, M., Magnuson, M.A., Heimberg, H., and Wright, C.V.E. (2013). Spatiotemporal patterns of multipotentiality in Ptf1a-expressing cells during pancreas organogenesis and injury-induced facultative restoration. Development *140*, 751–764.

Phan-Hug, F., Guimiot, F., Lelièvre, V., Delezoide, A.L., Czernichow, P., Breant, B., and Blondeau, B. (2008). Potential role of glucocorticoid signaling in the formation of pancreatic islets in the human fetus. Pediatr. Res. *64*, 346–351.

Picelli, S., Faridani, O.R., Björklund, A.K., Winberg, G., Sagasser, S., and Sandberg, R. (2014). Full-length RNA-seq from single cells using Smart-seq2. Nat. Protoc. *9*, 171–181.

Puri, S., and Hebrok, M. (2007). Dynamics of embryonic pancreas development using real-time imaging. Dev. Biol. *306*, 82–93.

Schaffer, A.E., Freude, K.K., Nelson, S.B., and Sander, M. (2010). Nkx6 transcription factors and Ptf1a function as antagonistic lineage determinants in multipotent pancreatic progenitors. Dev. Cell. *18*, 1022–1029.

Scheele, C.L.G.J., Hannezo, E., Muraro, M.J., Zomer, A., Langedijk, N.S.M., van Oudenaarden, A., Simons, B.D., and van Rheenen, J. (2017). Identity and dynamics of mammary stem cells during branching morphogenesis. Nature *542*, 313–317.

Shih, H.P., Wang, A., and Sander, M. (2013). Pancreas organogenesis: from lineage determination to morphogenesis. Annu. Rev. Cell. Dev. Biol. *29*, 81–105.

Shih, H.P., Panlasigui, D., Cirulli, V., and Sander, M. (2016). ECM signaling regulates collective cellular dynamics to control pancreas branching morphogenesis. Cell. Rep. *14*, 169–179.

Solar, M., Cardalda, C., Houbracken, I., Martín, M., Maestro, M.A., De Medts, N., Xu, X., Grau, V., Heimberg, H., Bouwens, L., et al. (2009). Pancreatic exocrine duct cells give rise to insulin-producing beta cells during embryogenesis but not after birth. Dev. Cell. *17*, 849–860.

Van Der Maaten, L., Hinton, G., and van der Maaten, G.H. (2008). Visualizing Data using t-SNE. J. Mach. Learn. Res. *18*, 2579–2605.

Ventura, A., Kirsch, D.G., McLaughlin, M.E., Tuveson, D.A., Grimm, J., Lintault, L., Newman, J., Reczek, E.E., Weissleder, R., and Jacks, T. (2007). Restoration of p53 function leads to tumour regression in vivo. Nature *445*, 661–665.

Villasenor, A., Chong, D.C., Henkemeyer, M., and Cleaver, O. (2010). Epithelial dynamics of pancreatic branching morphogenesis. Development *137*, 4295–4305.

Wang, S., Yan, J., Anderson, D.A., Xu, Y., Kanal, M.C., Cao, Z., Wright, C.V., and Gu, G. (2010). Neurog3 gene dosage regulates allocation of endocrine and exocrine cell fates in the developing mouse pancreas. Dev. Biol. *339*, 26–37.

Wilson, N.K., Kent, D.G., Buettner, F., Shehata, M., Macaulay, I.C., Calero-Nieto, F.J., Sánchez Castillo, M., Oedekoven, C.A., Diamanti, E., Schulte, R., et al. (2015). Combined single-cell functional and gene expression analysis resolves heterogeneity within stem cell populations. Cell Stem Cell *16*, 712–724.

Wu, T.D., and Nacu, S. (2010). Fast and SNP-tolerant detection of complex variants and splicing in short reads. Bioinformatics *26*, 873–881.

Zhou, Q., Law, A.C., Rajagopal, J., Anderson, W.J., Gray, P.A., and Melton, D.A. (2007). A multipotent progenitor domain guides pancreatic organogenesis. Dev. Cell *13*, 103–114.

STAR★METHODS

KEY RESOURCES TABLE

REAGENT or RESOURCE	SOURCE	IDENTIFIER
Antibodies		
Chromogranin A	Abcam	Cat# ab15160
Amylase	Santa Cruz	Cat# sc-12821
Ki67	Thermo Fisher	Cat# 14-5698-82
E-cadherin	BD Biosciences	Cat# 610181
E-cadherin	Abcam	Cat# ab11512
Ngn3	Santa Cruz	Cat# sc-13793
Cpa1	RD systems	Cat# AF2765
Sox9	Millipore	Cat# AB5535
Ptf1a	Bertrand Blondeau (Phan-Hug et al., 2008)	N/A
Dolichos biflorus agglutinin (DBA), biotinylated	Vectorlabs	Cat# B-1035
Tie2-APC	Biolegend	Cat# 124010
CD45-FITC	Biolegend	Cat# 103108
Chemicals, Peptides, and Recombinant Proteins		
Tamoxifen	Sigma	Cat# T5648-1G
Corn oil	Sigma	Cat# C8267
EdU	ThermoFisher Scientific	Cat# A10044
Tryple	ThermoFisher Scientific	Cat# A1217702
RapiClear 1.52	SunJin Lab	Cat# RC152001
Critical Commercial Assays		
EdU Click-iT Alexa Fluor 488 Imaging kit	Life technologies	Cat# C10337
Nextera XT DNA Preparation Kit	Illumina	Cat# FC-131-1096
Deposited Data		
Mapped reads from single-cell RNA sequencing analysis	This paper	National Center for Biotechnology Information GEO -accession number GEO: GSE89798
Experimental Models: Organisms/Strains		
Mouse: *Rosa26*-CreERT2; *Rosa26*-Confetti (pancreatic tissue)	Livet et al. (2007); Ventura et al. (2007)	N/A
Mouse: *Sox9*-CreERT2 (Kopp et al., 2011) (pancreatic tissue	The Jackson Laboratory	018829 strain
Software and Algorithms		
Volocity	Perkin Elmer	N/A
Imaris	Bitplane	N/A
Prism 7	Graphpad	N/A
R's statistics package	N/A	N/A
scater package	McCarthy et al. (2017)	N/A
scran package	Lun and Bach (2016)	N/A
destiny package	Laleh Haghverdi et al	N/A
G-SNAP	Wu and Nacu (2010)	N/A

CONTACT FOR REAGENT AND RESOURCE SHARING

Further information and requests for resources and reagents should be directed to, and will be fulfilled by, the Lead Contact, Benjamin D. Simons (bds10@cam.ac.uk).

EXPERIMENTAL MODEL AND SUBJECT DETAILS

Rosa26-CreERT2, and *Rosa26*-Confetti mice were described previously (Livet et al., 2007; Ventura et al., 2007). *Sox9*-CreERT2 (Kopp et al., 2011) mice were obtained from The Jackson Laboratory (018829 strain). Littermates were housed together and tissues collected before weaning. Since we did not find gender differences in the clonal data, results from mice of mixed gender were pooled. All mice were housed under specific pathogen-free conditions and all procedures were performed according to United Kingdom Home Office regulations. This research has been regulated under the Animals (Scientific Procedures) Act 1986 Amendment Regulations 2012 following ethical review by the University of Cambridge Animal Welfare and Ethical Review Body (AWERB).

METHOD DETAILS

Induction of Lineage Tracing

Tamoxifen (Sigma, T5648-1G) was prepared at 10mg/ml in corn oil (Sigma, C8267). Mice were intraperitoneally injected with Tamoxifen at 0.020, 0.025, 0.015 and 0.010mg per gram of pregnant female for E9.5, E12.5, E15.5 and E18.5 induction respectively. Analysis of pancreas from non-injected mice confirmed that *Rosa26*-CreERT2/*Rosa26*-Confetti and *Sox9*-CreERT2/*Rosa26*-Confetti system were not leaky (Figures S2A–S2D)

EdU Administration

EdU (ThermoFisher Scientific, A10044) was prepared at 10mg/ml in PBS. For EdU administration, mice were intraperitoneally injected at 20μg per gram of pregnant female.

Tissue Preparation

Embryonic and neonatal pancreas was fixed in 4% Paraformaldehyde (PFA) from 45 minutes to overnight, dependent on its developmental stage, and then washed in PBS extensively. For cryostat sectioning, samples were sucrose-equilibrated (30%) and mounted in OCT, and subsequently 100μm cryostat sectioned, or 7μm cryostat sectioned for Figures 6 and S7.

Tissue Staining

Thick 100μm cryostat sections were rehydrated in PBS. Sections and whole mount pancreata were blocked overnight in PBS, 2% donkey serum and 0.5% Triton-100X. The samples were incubated in primary antibodies (Chromogranin A from Abcam, ab15160 and Amylase from Santa Cruz, sc-12821) and Dolichos biflorus agglutinin (DBA), biotinylated (from Vectorlabs, B-1035) for 3 days at 4°C. Secondary antibodies were applied (from Life Technologies) and in AF647-Streptavidin (from Life Technologies) for 2 days at 4°C. EdU staining was performed on whole mount pancreata using EdU Click-iT Imaging Kit, according to the manufacturer's instructions (Life technologies, C10337), but with extended incubation of 5 hours at room temperature. Following the staining, all tissues were cleared with RapiClear 1.52 (from SunJin Lab, RC152001).

Thin sections were incubated in 2% Serum, 0.1% Triton-100X in PBS for 30 mins, subsequently incubated in primary antibody in 0.01%Triton-100X in PBS overnight (Ki67 from Thermo Fisher 14-5698-82, E-cadherin from BD Biosciences 610181, E-cadherin from Abcam ab11512, Ngn3 from Santa Cruz sc-13793, Cpa1 from RD systems AF2765, Sox9 from Millipore AB5535 and Ptf1a from Bertrand Blondeau (Phan-Hug et al., 2008), washed 3 times for 10 minutes the next day, and incubated with secondary antibody in 0.01%Triton-100X for 3 hours, washed and mounted.

Confocal Microscopy and Image Analysis

Images of whole mount and thick section pancreas were acquired using Leica TCS SP5 confocal microscope, using the tiling module. The images were analyzed with Volocity software. Parameters from imaged tissue such as volumes of objects, the 3D (x,y,z) coordinates of centers of objects, as well as tissue boundary points were recorded. By setting intensity thresholds manually for every image, the differently colored clusters were identified and the required parameters computed by the software. Information about the cluster constituent cell types was collected by looking at co-localization of clusters with a pancreatic marker in the Z-layer mode of Volocity. To obtain 3D reconstructed images from Z stacks from an image captured by the Leica SP5 microscope, Imaris management software (v8, Bitplane) was used.

Cell Fate Assessment

All images were analyzed. The fate assignment was based on DAPI, DBA and Chromogranin A stainings, and amylase in additional experiments (n=53 clones, 4 mice). To assess clonality with confidence, it was important to make use of the four-color confetti reporter system. As a result, it was necessary to image Chromogranin A and DBA in the same color channel. Nevertheless, ductal and islet cell types could be readily distinguished by morphology. The integrity of these cell-type assignments were verified by separate experiments co-staining with different colors: DBA and Chromogranin A; DBA and amylase; DBA, chromogranin A and amylase (Figure S4). Furthermore through careful optimization, the islets and ducts were stained and imaged at different and exclusive intensity ranges, as demonstrated in Figures S4D–S4F. The visualization reveals a close overlap between the heatmap and bimodal presentations (based on intensity) of ducts and islets within the same channel when compared with ducts and islets stained in two distinct and wave length-distant imaging channels.

To test more directly that cell identity could be reliably discriminated based on these markers, and, in particular, that acinar and ductal cells could be distinguished, the lineage tracing experiment was repeated from E12.5 to P14, and clones imaged together with amylase, DBA and chromogranin A co-staining. *53* clones were quantified (Figures 1M, S2O, S2P, and S3), and tested for whether the potency distributions (segregated into unipotent, bipotent and tripotent) were same as that observed in the original tracing. Crucially, no statistically significant difference (P=0.37, chi-square test) was found between the two sets of experiments. We also segregated both datasets to compare specifically the potency of acinar-containing clones in each. Similar to our results on global potency comparisons, we found no statistically significant differences (P=0.38, chi-square test)

Single-Cell RNA Sequencing

E13.25 and E15.25 pancreatic buds were collected from embryos of $n = 5$ and $n = 2$ pregnant females, respectively, and cells were dissociated in TrypLe-5X (ThermoFisher Scientific, A1217702) for 10 minutes at 37°C and the cell suspension immunostained. Single cells were individually sorted by fluorescence-activated cell sorting into wells of a 96-well polymerase chain reaction plate containing lysis buffer, with negative selection against blood cell marker Tie2-APC (clone Tek4, Cat: 124010, Biolegend), endothelial marker CD45-FITC (from clone 30-F11, Cat: 103108, Biolegend) and DAPI. scRNA-seq analysis was performed as described previously (Wilson et al., 2015; Picelli et al., 2014). The Illumina Nextera XT DNA Preparation Kit (Illumina, FC-131-1096) was used to prepare libraries. Pooled libraries were sequenced using the Illumina HiSeq 4000 system (single-end 50 bp reads). Reads were aligned using G-SNAP (Wu and Nacu, 2010) and the mapped reads were assigned to Ensembl genes (release 81) (Flicek et al., 2014) by HTSeq (Anders et al., 2015).

QUANTIFICATION AND STATISTICAL ANALYSIS

Statistics Reporting

In Figures 1K–1M, 2G and 2H, 3C and 3D, 4D, S2F–S2J, S2P, S5A and S5B, S6A–S6D, and S6G–S6J, *254* clones induced at E12.5 and traced until P14 were scored from n=3 mice. In Figures 1K, S5F–S5H, and S6E–S6H, 41 clones induced at E9.5 and traced until P14 were scored from n=3 mice. Error bars represent mean and S.D. or S.E.M. (outlined in Figure legends). Representative images from Figures 1C–1J, 2B, 2C, 2I, 2J, 5C, and S1B–S1O are based on observations from n=3 mice. Representative images from Figures 3A and 3B and S5K–S5O are based on observations from n=4 mice. Representative images from Figures 3F,3G, 3H, and 4A are based on observations from n=3 mice, each. In Figures 4I,4K, 4M representative images are based on observation from *n=3, 3, and 4* mice respectively. In Figures 5F–H, 5I and 5J, S2A–S2D, S3, and S4, representative images were based on *n=2, 2, 3, 4, and 6* mice respectively. Representative images from 6E, S7M-R are based on observations from n=3 mice.

In Figure S2M, pancreas mass was measured for n=3 mice (E18), n=5 mice (P7), n=11 mice (P14) and n=4 mice (P28). Error bars represent mean and S.E.M. In Figure S2N pancreas length, width and height were measured for n=3 mice (E13), n=3 mice (P14) and n=3 mice (P28). Error bars represent mean and S.E.M. In Figures 4C, 4D, and 4G and S6B, S6C, S6E, S6F, and S6I subtree sizes were measured for n=3 mice and *43* independent subtrees. Error bars represent mean and S.D.

In Figures 6A–6D and S7A–S7L, 516 single cells were isolated from n=7 mice for the single-cell RNA sequencing (*n=5* for E13.25 and *n=2* for E15.25). In Figures S5D and S5E, 84 clones induced at E12.5 and traced until P28 were scored from n=2 mice. Error bars represent mean and S.D. In Figures 5E and S6N, 78 clones induced at E15.5 and traced until P14 were scored from n=2 mice. In Figures 5E and S6O, 70 clones induced at E18.5 and traced until P14 were scored from n=2 mice. In Figures 1N and S2O 53 clones induced at E12.5 and traced until P14 were scored from n=4 mice.

Computational Analysis of Sequence Reads

To identify poor quality cells, three metrics were used: (1) the proportion of aligned reads, (2) the number of endogenous reads and (3) the number of features with more than 1 read. We filtered for cells with (1) more than 20% aligned reads, (2) more than 200,000 endogenous reads and (3) more than 5000 detected features. We only considered genes that were detected in at least 2 cells, with a variance greater than 0.001.

Out of the 672 cells that were captured in the experiment, 516 (77%) were used for downstream analysis. Reads were normalized using the deconvolution method as implemented in the scran package (Lun and Bach, 2016).

Dimensionality reduction was then performed using PCA as implemented in the scater package (McCarthy et al., 2017), resulting in two distinct groups of cells. Using the epithelial marker *Epcam*, one of these clusters was identified as being composed of pancreatic cells (303 cells). Focusing on the pancreatic cells alone, t-SNE was performed (Krijthe 2015) followed by k-means clustering from R's statistics package on the 500 most variable genes to identify the cellular identity of cells in the three clusters using known markers for acinar, ductal and islet cells.

To identify co-varying genes, Spearman's ρ was computed for a list of genes which, as per the literature, are associated with pancreatic development, and $2 - \rho$ was used as a dissimilarity index for hierarchical clustering. Using the destiny package (Angerer et al., 2016), a diffusion map (parameters: sigma=95, k=302) was employed and the DPT function was used to arrange cells in pseudo-time. Three distinct branches were identified and, based on the expression of proliferation markers (such as *Mki67*) and various specification and differentiation markers (such as *Cpa1* or *Neurog3*), the branch containing sample "SLX-11256.N701_S506" was identified as the one "earliest" in its developmental stage and used as a starting point to calculate the diffusion pseudo-time.

For the analysis of biological variance of gene expression, for each cluster and gene, the total variance was decomposed into technical and biological components using the trendVar and decomposeVar functions of the scran package (Lun et al., 2016).

Statistical Analysis of the Clonal Data

Given the observed cohesiveness of clones and relatively low labeling fraction used in the confetti lineage tracing assay, the spatial x,y and z coordinates of individual clones were first manually assessed, as well as their relative volume in terms of acinar, ductal and islet compartments (for the E9.5 to P14, E12.5 to P14 and E12.5 to P28 tracings). Detailed information for all tracings considered is supplied in a Table S1. All the plots presented in the main and supplemental figures were performed in Prism 8 (Graphpad).

The clonality of our manual reconstructions was challenged in several ways. Taking advantage of the fact that cells were labeled in four different colors (CFP, RFP, GFP and YFP), the probability of finding two clones of the same color at a given distance for all clonal pairs in a given pancreas was computed. It was reasoned that if clonal fragmentation had been underestimated in our reconstructions, then there should be an excess of clones of the same color at small distances (i.e. these would be fragments of the same clones that should be pooled together in a single unit). In the converse case, there should be a lack of clones of the same color at small distances. In the ideal case of a perfect clonal assay, all clones should have been independently induced, so that the probability of finding clones of the same color should be independent of distance, i.e. *P(r)* constant. In order to statistically assess such clonality, it was therefore necessary to build statistically upon what the null-hypothesis would give, i.e. "our assay is clonal".

In order to rescale the probability of finding two clones of the same color at a given distance, and in order not to be influenced by the complex shape and convolution of the pancreas, the probability of finding two clones of any color at a given distance was computed and used to rescale the probability of finding two clones of the same color. In the clonality hypothesis, clones are considered independent, so the resulting rescaled probability should be independent of distance. Next, a non-parametric bootstrapping method was used to build confidence intervals on the prediction from the null hypothesis. Thus, for each individual pancreas, the probability for a clone to have one of the four confetti colors was calculated, and colors of clones were randomly re-assigned according to this average property. This procedure was performed 1000 times, and in each case the rescaled probability *P(r)* was calculated. Finally, a 95% confidence interval at each distance *r* was calculated from this resampled probability distribution to assess how much experimental deviation of *P(r)* from a constant value can be due to random statistical variations. In Figure S2F these confidence intervals for each individual mouse induced at E12.5 and traced until P14 were plotted, together with the experimental measurements of *P(r)*. Notably, the experimental probabilities, although showing statistical variations, were found to be consistently included within the 95% confidence interval from the null-hypothesis; although, in some mice (such as mouse 1), a statistically significant excess of clones of the same color at short distances was observed. Thus, statistical correction was performed for this bias by grouping all clones of the same color within a given radius, in order to get back to the probability value expected from clonal induction (values shown by black dashed lines in Figure S2F).

To further test the clonality of the data, two independent controls were performed. First, it was noticed that one of the mice was stochastically induced at a dose twice as low as its two other counterparts (Figure S2G). The potency data was therefore segregated for each individual mouse in order to check whether the labeling dose influenced our results. For instance, one could have assumed that tripotency observed in the data resulted from unwanted merger of independent clones. Notably, however, the potency described in the main text was found to be faithfully reproduced for each individual mouse (Figure S2I), lending additional credence to our results.

Second, it was also noticed that the four confetti colors were represented in very different proportions (Figure S2H, with all three mice grouped for this statistical assay). In particular, there were more than 5 times as many RFP clones than CFP clones, with GFP and YFP clones sitting at an intermediate probability. The potency data was therefore segregated for each individual color, and it was found that that the degree of unipotency was independent of the color examined (Figure 1L) and that, in general, the ratio of unipotency, bipotency and tripotency were consistent in all colors examined (Figure S2I). This, again, argues strongly in favor of the clonality of the data, given that CFP cells consisted of less than 0.3% of all pancreatic cells.

Finally, to compare the rescaled distributions of clone sizes at different time points, as well as the clone size versus subtree size distribution in the theory and pancreas subtree data, a Mann-Whitney test was used and P-values reported. (Binning was used for very small clone sizes, as the distribution for rescaled acinar clone size reached much smaller values than ductal clone and subtree size distributions.) In all cases, P-values were also calculated from two-sample Kolmogorov-Smirnoff tests to check our results: 1. rescaled ductal versus acinar compartment size distribution at P14: P=0.68, Mann-Whitney test and P=0.18, Kolmogorov-Smirnoff test; 2. rescaled ductal compartment size distribution at P14 versus P28, P=0.95, Mann-Whitney test, P=0.44, Kolmogorov-Smirnoff test; 3. rescaled acinar compartment size distribution at P14 versus P28, P=0.63, Mann-Whitney test, P=0.78, Kolmogorov-Smirnoff test; 4. Rescaled ductal compartment (P14) versus subtree size distribution, P=0.6, Mann-Whitney test, P=0.34, Kolmogorov-Smirnoff (note that here we excluded small-unbranched clones, as we wished to assess the large-scale heterogeneity from branching morphogenesis); 5. distribution of number of branches per clone induced at E12.5 versus E15.5: P=0.01, Mann-Whitney test, P=0.04, Kolmogorov-Smirnoff test (Figure S5I); 6. distribution of the number of branches per clone (induced at E12.5, Figure S5C) – center (inner two-thirds of the pancreas) versus periphery (outer third of the pancreas): P=0.007, Mann-Whitney test, P=0.04, Kolmogorov-Smirnoff test.

Quantile-quantile plots were also computed to compare the coefficient of determination R^2 of to the predicted curve, *f(x)=x*, if the distributions were perfectly identical. It was found that R^2=0.87 for the E18 subtree size distribution versus the P14 acinar clone size

distribution, R^2=0.83 for the E18 subtree size distribution versus the P14 ductal clone size distribution, and R^2=0.99 for the P14 acinar clone size distribution versus the P14 ductal clone size distribution (Figures S6B and S6C). Moreover, it was found that R^2=0.89 (respectively R^2=0.93) when comparing the P14 acinar (respectively ductal) clone size distribution versus the subtree size distribution measured for mammary gland branching morphogenesis (Figures S6I and S6J). (Data from mammary gland was acquired from Scheele et al., 2017.) These findings are indicative of a close correspondence between the shape of these various distributions; although small differences are bound to arise naturally, for example, due to differential dynamics in ductal and acinar precursors on the background of branching morphogenesis, or specific geometric constraints in mammary gland compared to pancreatic branching morphogenesis.

Note that for the E15.5 to P14 and E18.5 to P14 tracings, the potency was only assessed via the same methods as described before. In order to ensure clonality, given that the density of labeled clones was higher, only the rare colors were used in the quantifications of Figure 5E (CFP and GFP, see Figures S6N and S6O).

For details on the theoretical analysis of branching morphogenesis, the comparison between model and experiment, and the theoretical analysis of monoclonal conversion during branching morphogenesis, see Methods S1.

DATA AND SOFTWARE AVAILABILITY

The accession number for the mapped reads from single-cell RNA sequencing analysis reported in this paper has been uploaded to National Center for Biotechnology Information GEO (accession number GEO: GSE89798).

Developmental Cell, Volume *46*

Supplemental Information

Defining Lineage Potential and Fate Behavior of Precursors during Pancreas Development

Magdalena K. Sznurkowska, Edouard Hannezo, Roberta Azzarelli, Steffen Rulands, Sonia Nestorowa, Christopher J. Hindley, Jennifer Nichols, Berthold Göttgens, Meritxell Huch, Anna Philpott, and Benjamin D. Simons

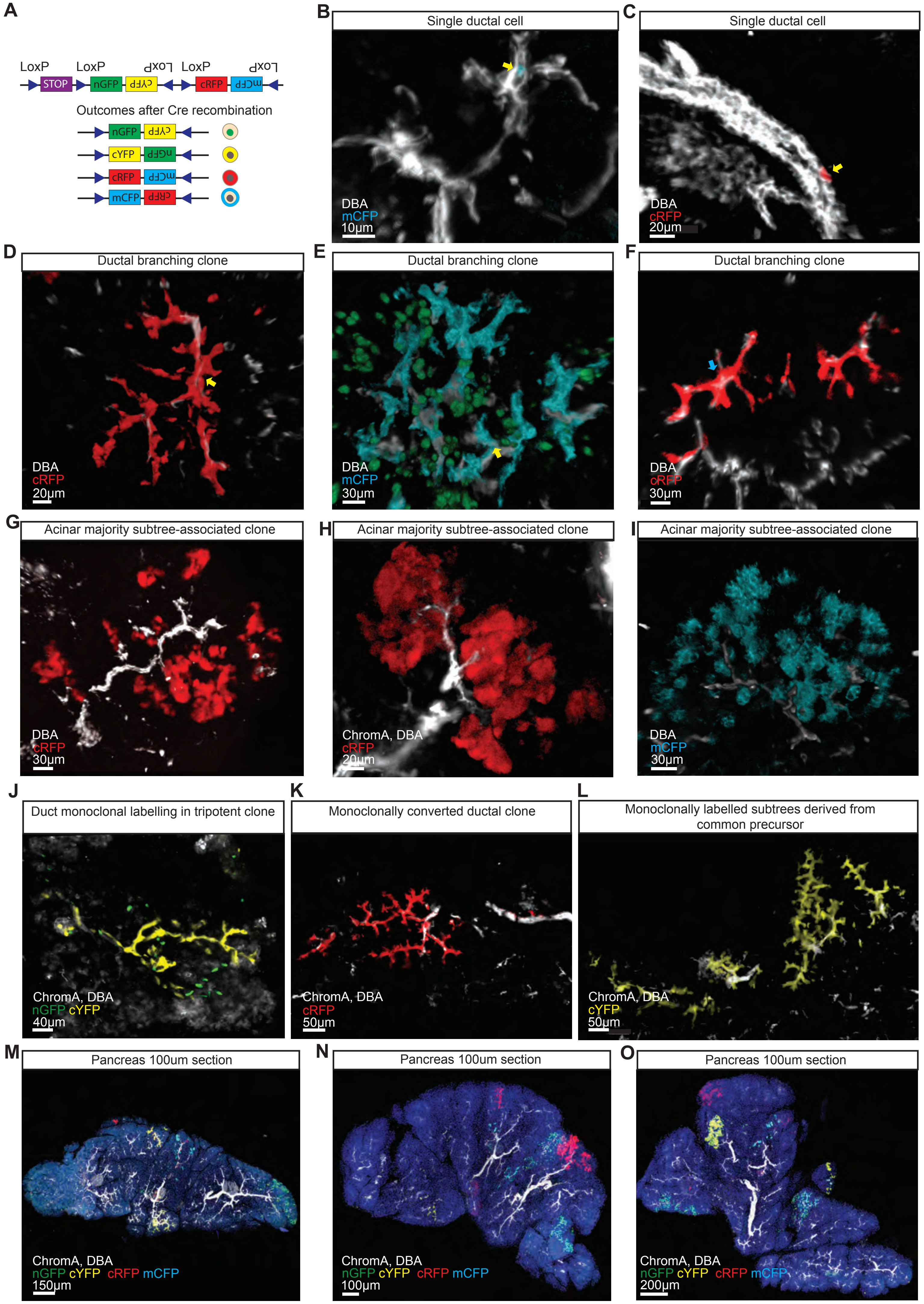

Supplemental Figure 1

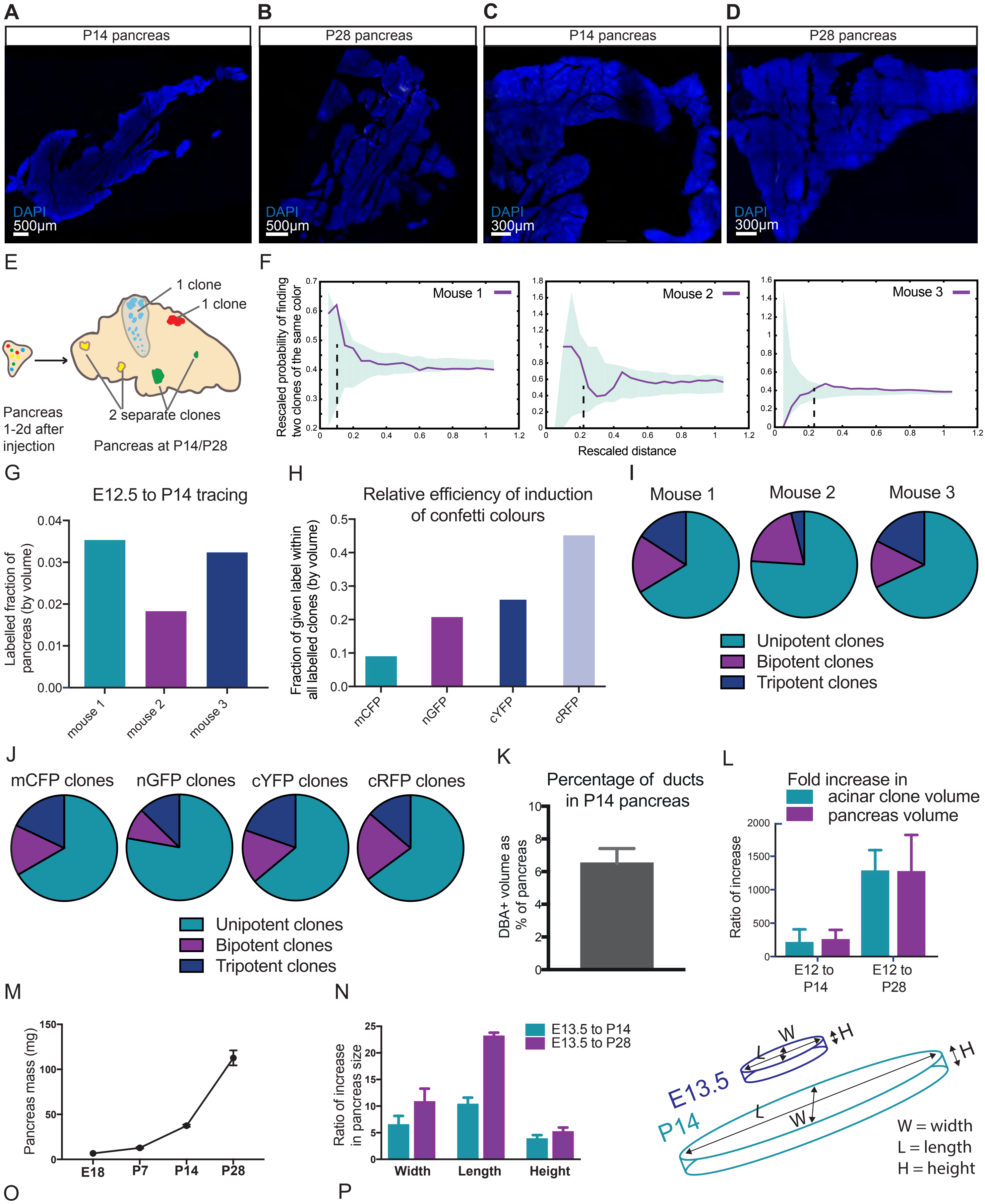

O

E12.5 to P14 - amylase control

Clone type	Frequency (%)	Number
Unipotent acinar	54.7	29
Unipotent ductal	7.5	4
Unipotent islet	3.8	2
Bipotent acinar-ductal	20.8	11
Bipotent acinar-islet	0.0	0
Bipotent ductal-islet	1.9	1
Tripotent	11.3	6

P

E12.5 to P14

Clone type	Frequency (%)	Number
Unipotent acinar	59.9	154
Unipotent ductal	3.1	8
Unipotent islet	5.1	13
Bipotent acinar-ductal	10.5	27
Bipotent acinar-islet	4.7	12
Bipotent ductal-islet	1.2	3
Tripotent	15.6	40

Supplemental Figure 2

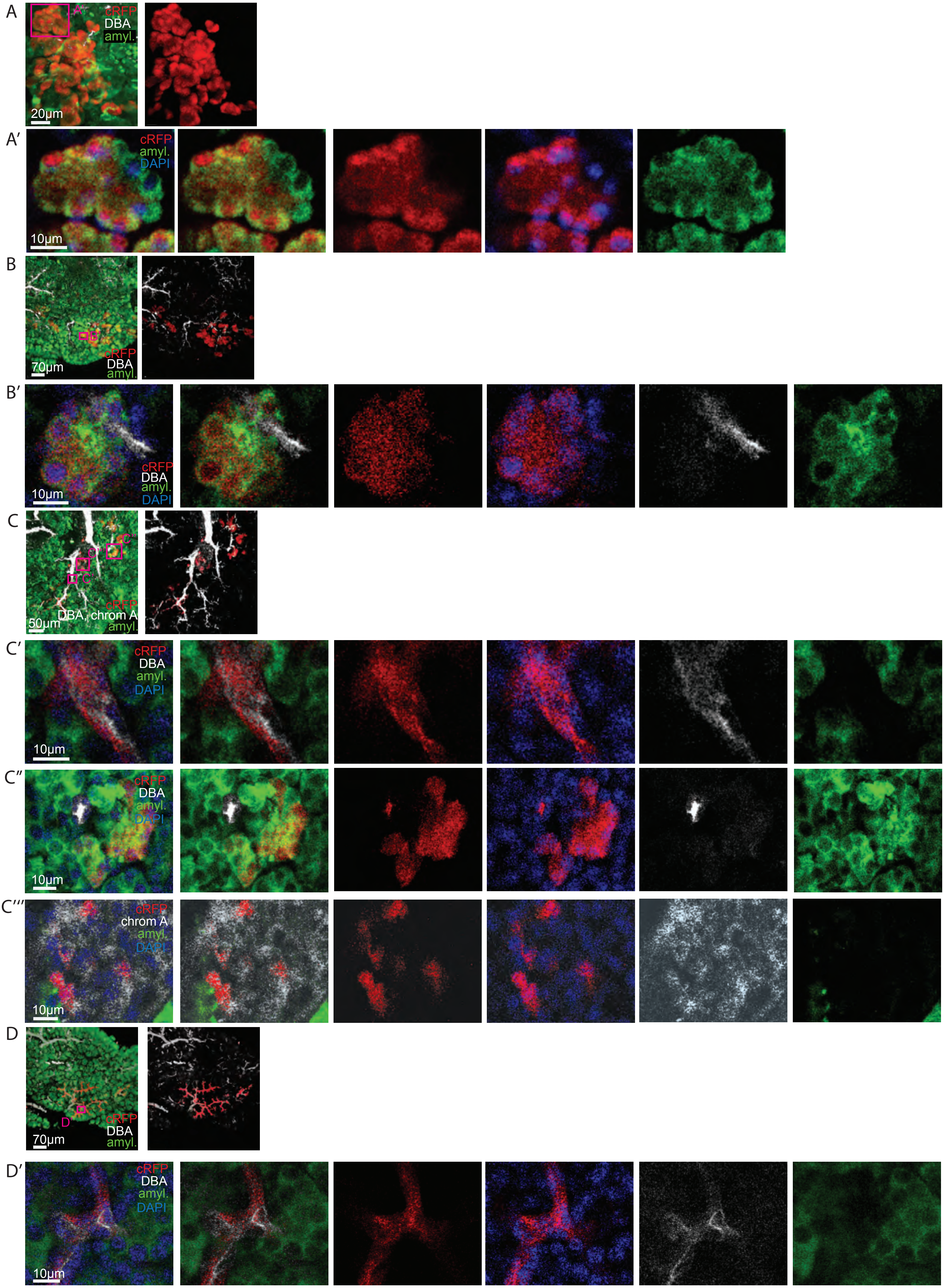

Supplemental Figure 3

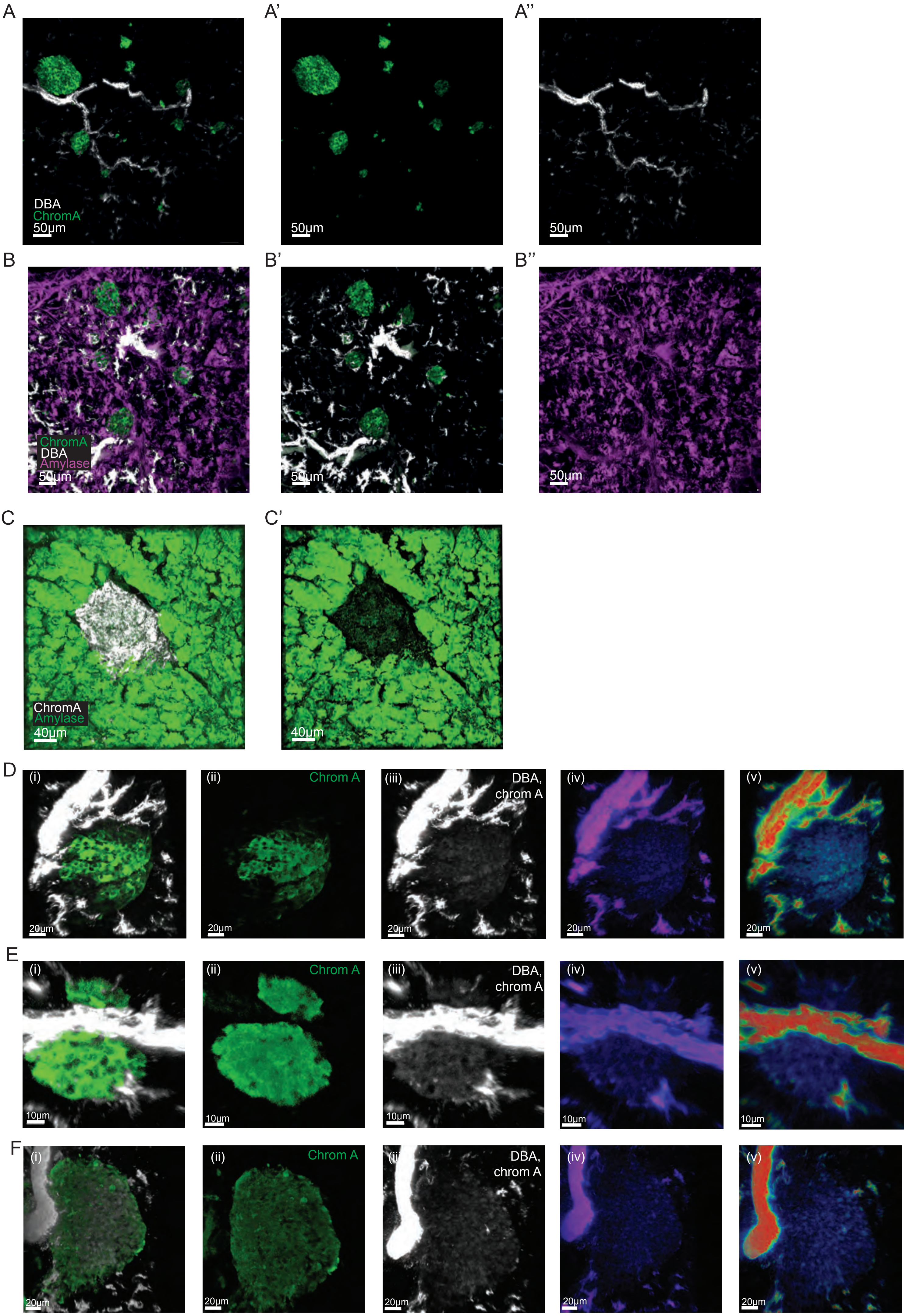

Supplemental Figure 4

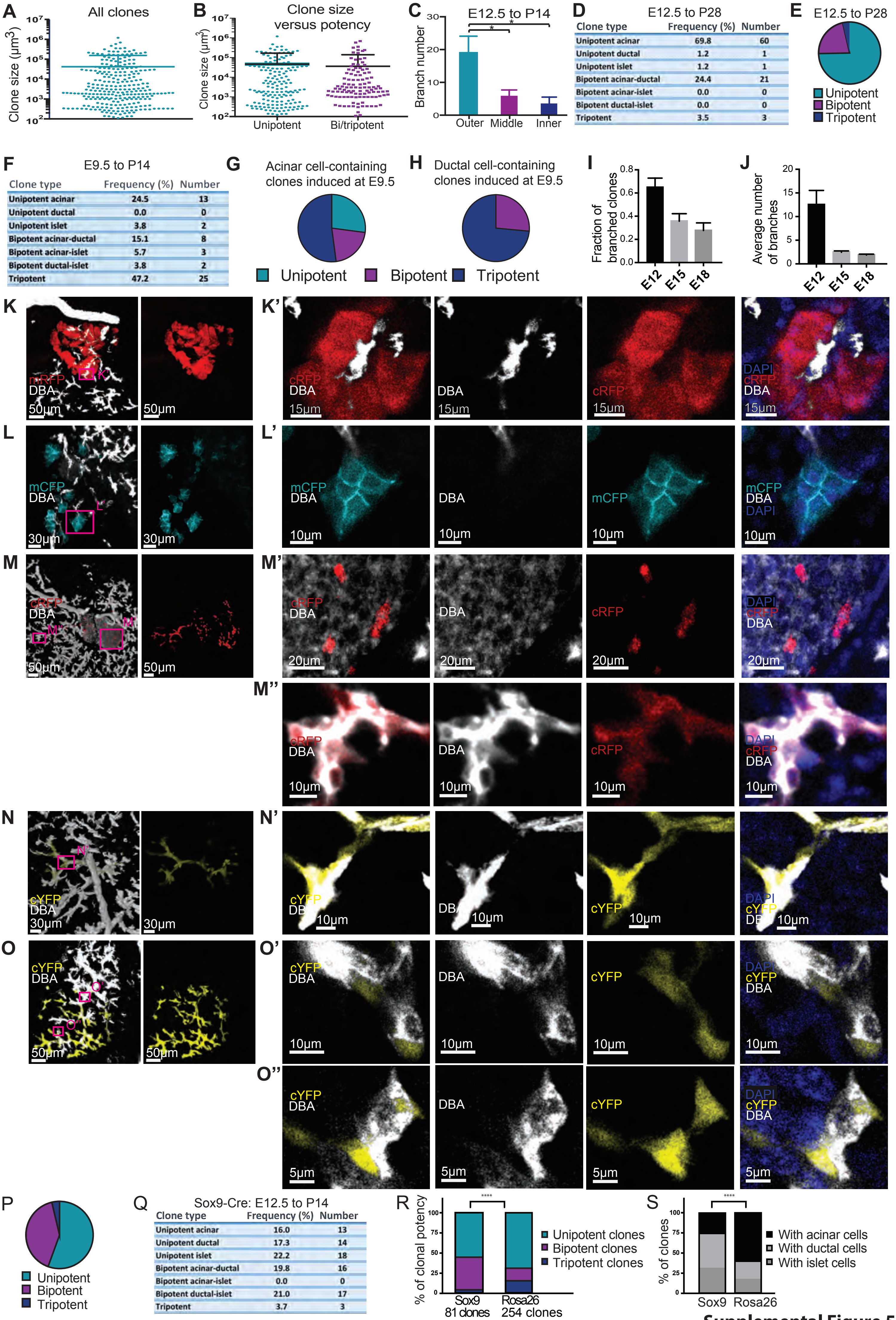

D — E12.5 to P28

Clone type	Frequency (%)	Number
Unipotent acinar	69.8	60
Unipotent ductal	1.2	1
Unipotent islet	1.2	1
Bipotent acinar-ductal	24.4	21
Bipotent acinar-islet	0.0	0
Bipotent ductal-islet	0.0	0
Tripotent	3.5	3

F — E9.5 to P14

Clone type	Frequency (%)	Number
Unipotent acinar	24.5	13
Unipotent ductal	0.0	0
Unipotent islet	3.8	2
Bipotent acinar-ductal	15.1	8
Bipotent acinar-islet	5.7	3
Bipotent ductal-islet	3.8	2
Tripotent	47.2	25

Q — Sox9-Cre: E12.5 to P14

Clone type	Frequency (%)	Number
Unipotent acinar	16.0	13
Unipotent ductal	17.3	14
Unipotent islet	22.2	18
Bipotent acinar-ductal	19.8	16
Bipotent acinar-islet	0.0	0
Bipotent ductal-islet	21.0	17
Tripotent	3.7	3

Supplemental Figure 5

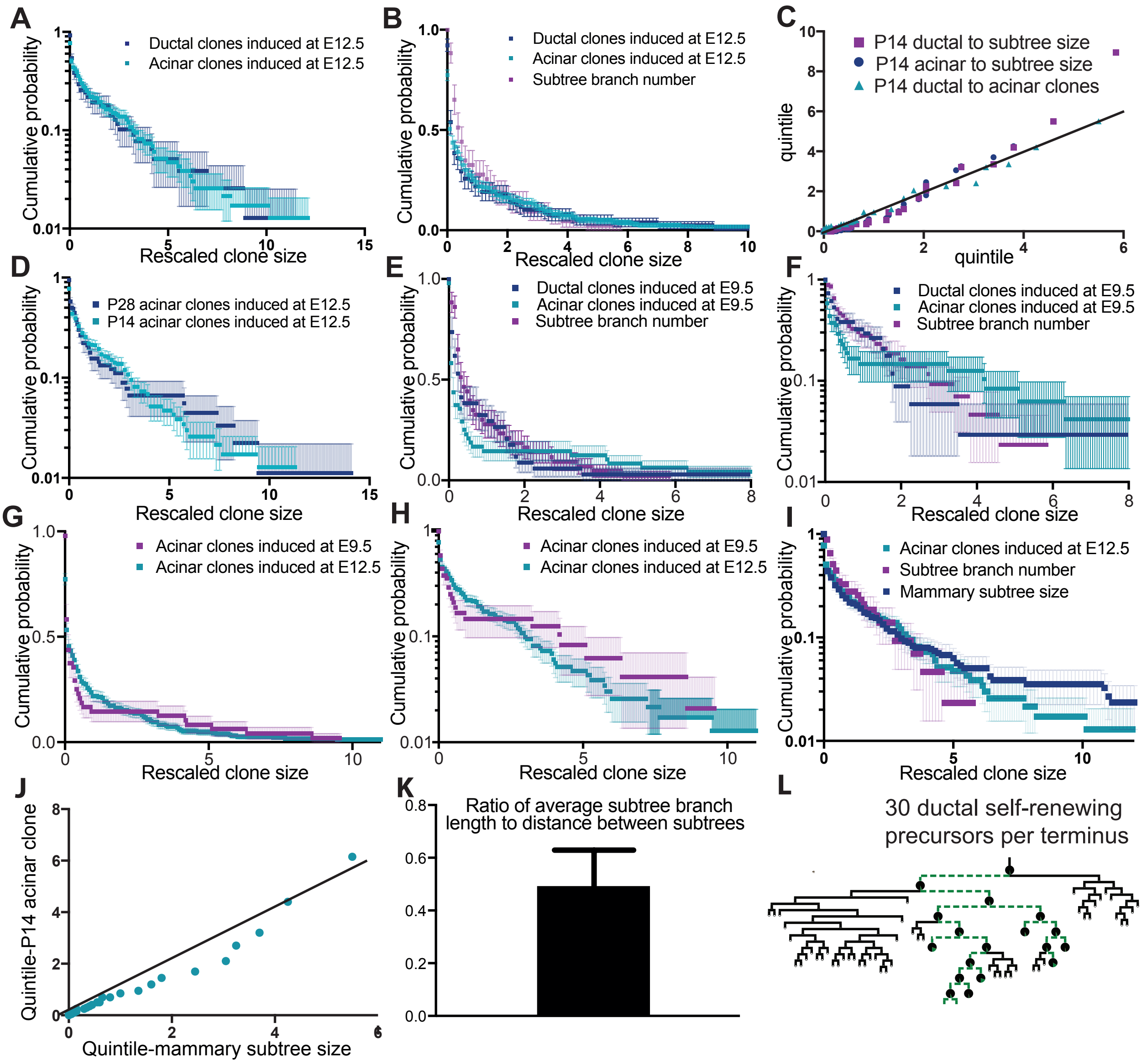

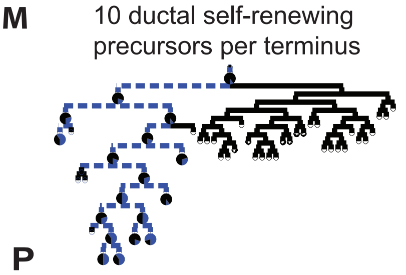

N E15.5 to P14

Clone type	Frequency (%)	Number
Unipotent acinar	80.8	63
Unipotent ductal	2.6	2
Unipotent islet	0.0	0
Bipotent acinar-ductal	14.1	11
Bipotent acinar-islet	0.0	0
Bipotent ductal-islet	2.6	2
Tripotent	0.0	0

O E18.5 to P14

Clone type	Frequency (%)	Number
Unipotent acinar	77.0	47
Unipotent ductal	9.8	6
Unipotent islet	3.3	2
Bipotent acinar-ductal	9.8	6
Bipotent acinar-islet	0.0	0
Bipotent ductal-islet	0.0	0
Tripotent	0.0	0

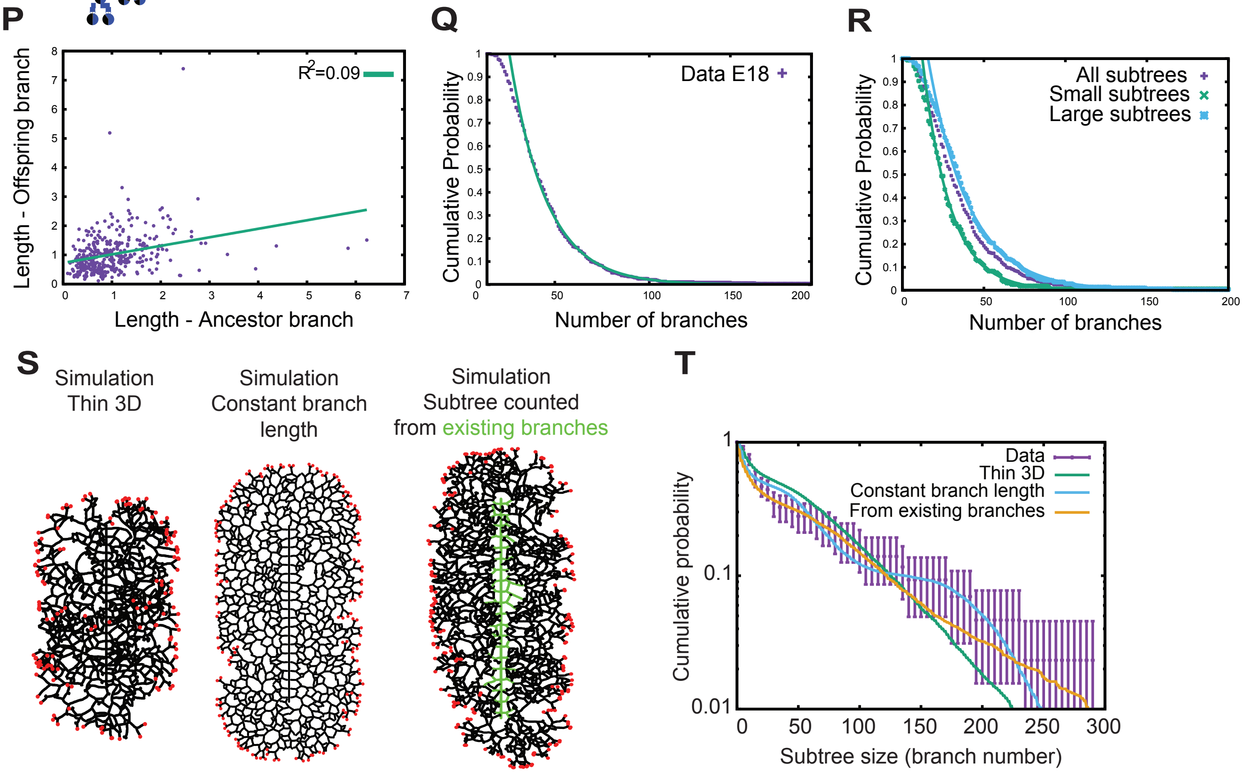

Supplemental Figure 6

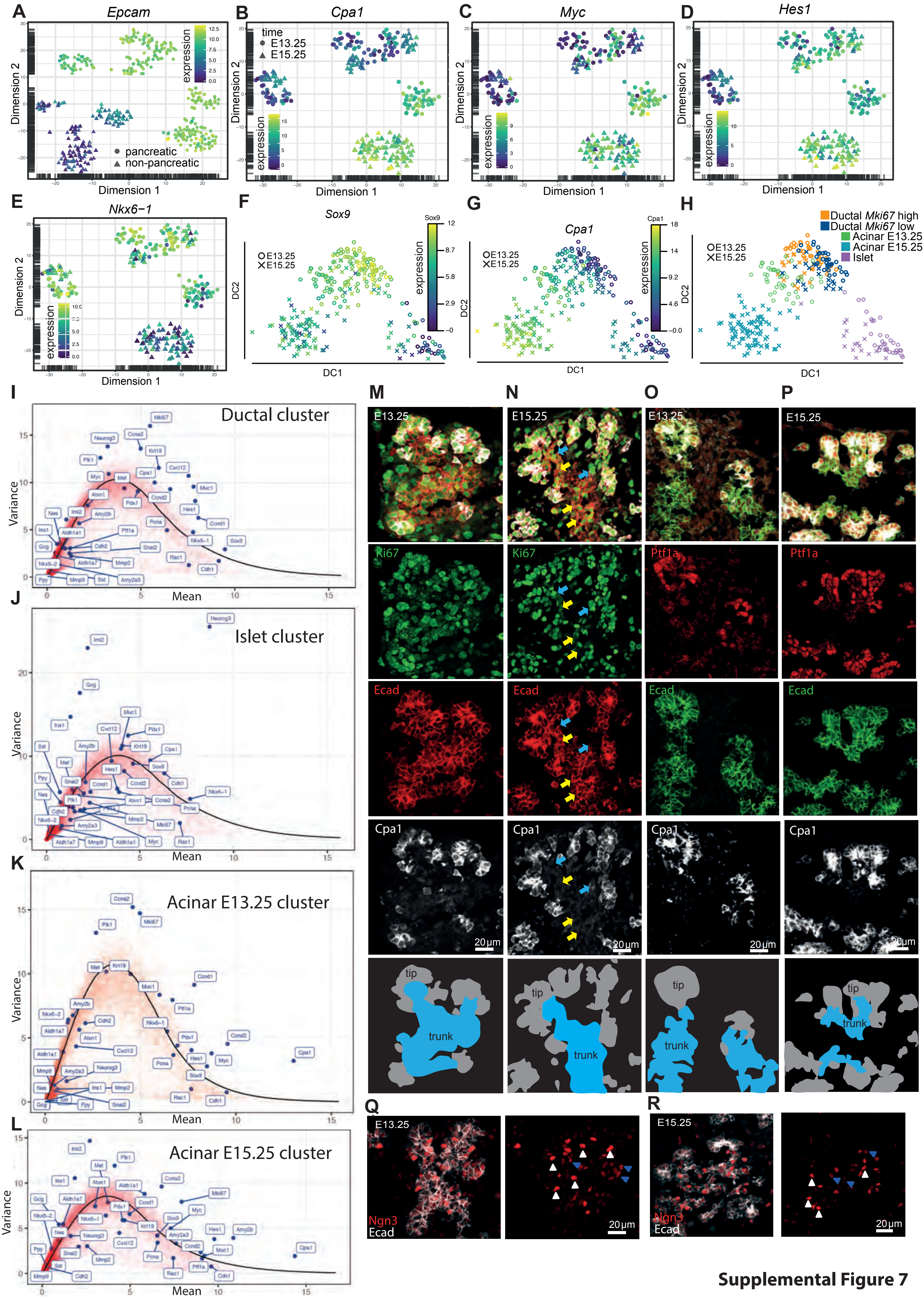

Supplemental Figure 7

Supplemental Figure 1. Related to Figures 1 and 2. Typical examples of acinar, ductal and islet clones.

(**A**) The basis of the *Rosa26*-Confetti reporter system and fluorescent protein expression (nuclear GFP – nGFP, cytoplasmic YFP – cYFP, cRFP, and membrane CFP – mCFP). (**B,C**) Single-cell ductal clones, (**D-F**) ductal monoclonal clones, (**G-I**) subtree-associated acinar-majority clones, and (**J-L**) ductal terminus-associated clones with (J) islet contribution – cYFP clone. (**M-O**) Thick 100µm sections of P14 pancreas of *Rosa26*-Cre; *Rosa26*-Confetti mice induced at E12.5 that were used for clonal analysis. The images were created with the tiling panel on a confocal microscope Leica SP5 and reveal the morphology of clones. Ducts, islets and acini indicated with arrow, arrowhead and asterisk, respectively.

Supplemental Figure 2. Related to Figure 1. Verifying the integrity of clonal assignments and robustness of the potency results.

(**A,B**) 100µm pancreatic sections from *Rosa26*-CreT2; *Rosa26*-Confetti (n=3mice) and (**C,D**) *Sox9*-CreERT2; *Rosa26*-Confetti (n=3mice) non-injected mouse at, respectively, P14 and P28 shows that the constructs were not leaky. (**E**) Schematic indicating methods of clonal assignment based on cohesiveness and distances between constituent labeled cell clusters (Theory Methods). (**F**) Rescaled probability (purple thick line) of finding another clone of the same color as a function of rescaled distance from a given clone for all three *Rosa26*-Confetti mice induced at E12.5 and traced until P14 (left, center and right panels). For each mouse, the clone-clone distance was rescaled by the average distance in three-dimensions between two clones randomly chosen across sections of a given pancreas. The probability of finding a clone of the same color at a given distance was rescaled by the probability of finding two clones of any color at the same distance. The shaded lines represent the 95% confidence interval of rescaled probability under the assumption that the data was clonal. The dashed black line shows the distance used for fragment grouping to define a clone. (**G**) Fraction of fluorescently labeled cells at E12.5 in the P14 pancreas of different mice. (**H**) Color balance of confetti clones induced at E12.5 and traced until P14, grouping all three pancreases. Cyan is the least represented color while Red is the most represented. (**I**) Global potency of P14 clones induced at E12.5, segregated for each individual mouse, showing that the potency of each is consistent with the ensemble average (Figure 1K), and independent of the frequency of induction shown in H. (**J**) Global potency of P14 clones induced at E12.5, segregated for each individual confetti color. (**K**) Percentage of pancreas that belongs to the ductal compartment at P14. (**L**) Fold increase in the size of the pancreas as a whole and in the acinar compartment of clones between E13.5 and P14, and E13.5

and P28, indicating that clones are representative of tissue. Bars indicate mean and S.E.M. Growth characteristics of the murine pancreas including (**M**) changes in pancreatic mass between E18.5 and P28; (**N**) fold-change in pancreas width, length and height at E13.5, P14 and P28, as defined in the schematic inset. Bars indicate mean and S.E.M. Frequency of different clone types by cell composition for (**O**) E12.5 induction with 53 clones (*n=4* mice) quantified with additional amylase co-immunostaining in additional to DBA and chromogranin A, and (**P**) E12.5 induction with 254 clones (*n=3* mice) quantified (same dataset as Figure 1K).

Supplemental Figure 3. Related to Figure 1. Additional verification of clone types with amylase immunostaining.

(**A,B**) Branch-associated cRFP clones in 3D projection and in (**A',B'**) individual Z-stack zoomed from the blue box in (A,B) reveal complete overlap with the acinar amylase immunostaining (green). (**C**) shows a 3D projection of a tripotent clone with a ductal branching component (re-stained clone from Figure 2I) with individual Z-stack sections presented in (**C',C''**) zoomed from a blue box in (C). (C') shows a clone part overlapping completely with the ductal DBA (white), and showing no overlap with acinar amylase (green). (C'') shows a clone part overlapping with amylase (green, arrowhead) and a part overlapping with islet (white, arrow). (**D**) 3D projection of ductal branching clone (re-stained clone from Figure S1K) with individual Z-stack sections presented in (**D',D''**) zoomed from the blue box in (D). (D',D'') show complete overlap of a clone with the DBA (white) ductal labeling. DBA, chromogranin A are grey, amylase is green, DAPI is blue. Note that individual nuclei can be observed with cytoplasmic amylase surrounding the nucleus in a cloud-like fashion.

Supplemental Figure 4. Related to Figure 1. Verification of marker staining.

(**A**) Combination of two markers for islet chromogranin A (green) and ductal DBA (white) alone shows that the two compartments display clear morphological differences and therefore can be stained in one channel for potency analysis of confetti+ cells. Since hallmark morphological differences distinguish ductal and islet compartments, in the clonal lineage study, these features can be imaged using the same channel. This allows us to reserve other channels for DAPI staining and to discriminate all of the fluorescent reporters used in the confetti tracing. (**B**) Combination of all three markers with islet chromogranin A (green), ductal DBA (white) and acinar amylase (pink) shows that all the markers are exclusive and the vast majority of DBA-

chromogranin A- tissue is amylase+. (**C**) The combination of two markers for islet chromogranin A (white) and acinar amylase (green), shows complete mutual exclusivity. Visualization showing exclusive intensity ranges distinguishing imaging of ducts and islets, following careful optimization of the immunostaining protocol. The islets were stained with DBA in 647nm and chromogranin A in both 488nm and 647nm. (**D-F**) Various examples of islets and ducts from left to right for the same section: (i) overlay of white (647nm channel) and green (488nm channel) shows the distinction between the islets and the ducts; (ii) green (488nm) shows islets only; (iii) white (647nm) raw image shows the combination of islet and ductal staining, revealing a lower intensity of islet staining (dimmer area) than the ducts (significantly brighter); (iv-v) representation of image from (iii) white (647nm) channel in (iv) ultra and (v) spectral intensity mode in Imaris, demonstrating that the two pancreatic structures are stained at distinct and exclusive intensities showing a perfect match with the chromogranin A and DBA stained in two distinct and well-separated imaging channels (488nm and 647nm), as in (i).

Supplemental Figure 5. Related to Figures 1 and 3. Quantification of clone types with their relative frequency and respective sizes.

(**A**) Complete size distribution for all clones induced from E12.5 to P14. (**B**) Comparison of the same clones as (A) but segregating unipotent and multipotent clones. Average size +/-S.D. are shown. (**C**) Comparison of branch number of branching clones in outer, middle and inner third of the thick tissue clones reveals statistically significant difference between outer and middle/inner thirds ($p < 0.01$ – Mann-Whitney). Average branch numbers +/-S.E.M. are presented. (**D**) Table of all clone types traced from E12.5 to P28. (**E**) Global potency of clones traced from E12.5 to P28. (**F**) Table of all clone types traced from E9.5 to P14. (**G**) Potency of acinar-containing clones traced from E9.5 to P14. (**H**) Potency of ductal-containing clones traced from E9.5 to P14. (**I,J**) Comparison of the fraction of branched clones (I) and average number of branches per clone (J) from the E12.5, E15.5 and E18.5 tracings (all traced until P14). Branched clones are defined as those that belong to individual subtrees with at least 3 ductal branches containing labeled cells of the same color, or 3 acini clusters at the termini of different branches containing labeled cells of the same color. Averages +/- S.E.M are shown. (**K-O**) Representative images of *Sox9*-CreERT2 traced clones recapitulating the acinar and ductal branching clones observed with *Rosa26*-CreERT2 tracing. (K,L) presents acinar tree shaped clones surrounding the network of ducts with individual Z-stacks presented in (**K’,L’**). (M-O) Clones with a ductal branching element. (M) Ductal-islet bipotent clone with individual Z-stack images presented in (**M’,M’’**) – islet part in (M’) and ductal part in (M’’). (N,O) Ductal-only clones with individual Z-stacks presented in (**N’**) and (**O’,O’’**)

showing co-localization with DBA and no co-localization with amylase. (**P**) Potency quantification for *Sox9*-CreERT2 lineage tracing between E12.5 and P14. (**Q**) Frequency and number of individual clone types for *Sox9*-lineage tracing. (**R**) Comparison of E12.5 to P14 *Rosa26* and *Sox9* lineage tracing reveals statistically significant difference in the potency outcome. (**S**) Fraction of clones with individual compartments present (acinar, ductal, islet). *Sox9*-traced clones reveal an enrichment in the ductal fraction.

Supplemental Figure 6. Related to Figures 1,4 and 5. Comparison between subtree size distributions and clone size distribution at E9.5 and E12.5.

(**A**) Cumulative clone size distribution (plotted on a logarithmic scale) of ductal and acinar compartments from E12.5 induction. Clone sizes here and below were rescaled by their respective ensemble averages. S.D are shown in all cumulative clone size distributions. (**B**) Cumulative clone size distribution (plotted on linear scale) of ductal and acinar compartments from E12.5 induction overlaid with the cumulative subtree size distribution (number of branches) determined from E18.5 pancreas. (**C**) Quintile-quintile plot comparing cumulative size distribution of P14 ductal compartment clones (induced at E12.5) versus P14 acinar compartment clones (induced at E12.5) in cyan; P14 ductal compartment clones (induced at E12.5) versus pancreas subtree size (at E18.5) in pink; P14 acinar compartment clones (induced at E12.5) versus pancreas subtree size (at E18.5) in dark blue. (**D**) Cumulative size distribution (plotted on logarithmic scale) of rescaled P14 and P28 clone sizes both induced at E12.5. (**E,F**) Cumulative size distribution (plotted on linear and log y-axis) of ductal and acinar compartments in clones induced at E9.5 overlaid with the cumulative subtree size distribution (number of branches) determined from E18.5 pancreas. (**G**,**H**) Cumulative size distribution (plotted on linear and logarithmic scale) of acinar compartments in clones induced at E9.5 and E12.5. (**I**) Cumulative size distribution (plotted on logarithmic scale) of acinar compartments in clones induced at E12.5, pancreatic subtree size and mammary gland epithelium subtree size (Scheele et al. 2017), showing similar growth characteristics between developing pancreas and mammary gland. (**J**) Quintile-quintile plot comparing the cumulative size distribution of P14 acinar compartment clones (induced at E12.5) versus experimental mammary subtree size (Scheele et al. 2017). (**K**) Ratio of the average branch length (distance between two branch points) by the average distance between two neighboring subtrees (along the main duct), quantified in E18 pancreas, used as an input for the simulations of Figure 4F,G. Average ratio +/- S.E.M. are shown. (**L,M**) Simulation of a theoretical subtree containing (L) 30 and (M) 10 precursors per active ductal terminus with one labeled self-renewing precursor cell. (**N**) Table of all clone

types traced from E15.5 to P14. (**O**) Table of all clone types traced from E18.5 to P14. (**P**) Relationship between the length of a branch and the length of its offspring branch, showing little correlation. (**Q,R**) Branch length distribution across all subtrees (Q) or segregated between small and larger subtrees (R), showing a consistently good fit by an exponential with small waiting time (thick lines), indicative of a stochastic branching process. (**S,T**) Sensitivity analysis for the branching-annihilating random walk (BARW) simulations, considering alternative models, shown as characteristic simulation output (S) or subtree size distribution (right, 500 simulations each). For left to right: simulation in a thin 3D setting (green theory line), for constant branch length (blue theory line) and for subtrees arising from existing branches (generation 1, shown in green) (orange theory line). The simulations show that these small variations, keeping the core of BARW hypothesis intact, do not significantly impact the predictions.

Supplemental Figure 7. Related to Figure 6. Single-cell RNA sequencing – gene-specific graphs.

(**A-E**) t-SNE plots, where each cell is colored by the log-normalized expression level of (A) *Epcam,* (B) *Cpa1,* (C) *Myc,* (D) *Hes1,* (E) *Nkx6-1*, enabling recognition of (B,C) the acinar cluster together with Figure 6C, and (D,E) the ductal cluster together with Figure 6D. (B-E) t-SNE plots contain E13.25 (circle) and E15.25 (triangle) cells showing close clustering of cells from the two time-points; in (A) circles and triangles represent pancreatic and non-pancreatic cells. The shape indicates cluster association as determined by k-means clustering. (**F-H**) Diffusion pseudotime (DPT) plots with both E13.25 (circles) and E15.25 (crosses) cells indicating that (F) *Sox9* is anti-correlated ($P < 5.5\ 10^{-12}$) with DPT direction, i.e. expression is upregulated at the apex, while (G) *Cpa1* is correlated ($P < 5.5\ 10^{-12}$) with DPT direction, i.e. expression is upregulated in differentiating cells. (F-G) Each cell is colored by the log-normalized expression level. (H) DPT plot for *Mki67* showing *Mki67* high cells segregating towards acinar branch and *Mki67* low cells segregating towards islet branch, consistent with the lineage tracing data, which indicate that acinar and ductal cells retain full proliferative potential. (**I-L**) Variance of genes (red) in a given cluster as a function of the mean expression level for (I) ductal, (J) islet, (K) acinar E13.25, and (L) acinar E15.25 clusters, revealing that *Cpa1* and *Sox9* are indeed heterogeneously expressed in ductal cells (I), *Sox9* is homogeneous in acinar cells (K,L), and *Ptf1a* shows homogenous expression in the E15.25 acinar cluster (J). *Ngn3* is expressed heterogeneously within the islet cluster (J), consistent with the literature (Bankaitis et al. 2015). (**M-R**) Immunostained 7μm thin pancreatic sections of Ecadherin, Cpa1 and Ki67 at (M) E13.25 and (N) E15.25 showing enrichment in Ki67 in Cpa1+ tip areas at E15.25. In Ecadherin+ Cpa1- trunk regions,

there is a slight enrichment of Ki67-high cells at peripheral regions indicated by blue arrows, while Ki67-low cells in more central parts are indicated by yellow arrows. (**O,P**) E-cadherin, Cpa1 and Ptf1a immunostaining at (O) E13.25 and (P) E15.25 shows a relatively homogenous expression of Ptf1a, which also overlaps with Cpa1 expression. (**Q,R**) E-cadherin and Ngn3 immmunostaining reveals Ngn3 expression heterogeneity (as previously reported, Bankaitis et al. 2015) with Ngn3-high cells indicated by blue arrowhead and Ngn3-low cells indicated by white arrowheads at (Q) E13.25 and (R) E15.25.

Methods S1. Related to STAR Methods

Theoretical analysis of branching morphogenesis and monoclonal conversion

Pancreas development involves a two-stage process in which a phase of plexus formation and remodeling precedes a phase of branching morphogenesis (although some temporal overlaps may exist between the two phases, Bankaitis et al. 2015). Although cell fate behavior and clonal dynamics during the phase of plexus remodeling is challenging to resolve, the observed localization of clonal fragments to individual ductal subtrees (see main text) indicates that the large-scale structure of the pancreas is dominated by the secondary phase of branching morphogenesis involving serial rounds of dichotomous ductal bifurcation. In the following, the strategy to define the dynamics of pancreatic progenitors during this secondary phase of expansion is detailed, focusing on clonal data obtained from the E12.5 induction time-point. In developing this program, use was made of a model introduced recently to describe the process of branching morphogenesis in several mammalian organs, including the mouse mammary gland epithelium and kidney, as well as human prostate (Hannezo et al. 2017). Specifically, in the context of the mammary gland (Scheele et al. 2017), it has been shown that the statistics of the branched ductal network are consistent with a remarkably simple paradigm in which functionally equivalent (i.e. equipotent) "active" terminal end-buds of ducts choose stochastically between ductal bifurcation and termination (inactivation), where further bifurcation ceases. Based on the success of this model in predicting quantitatively the statistical distributions of branching patterns and clonal data (Hannezo et al. 2017), here the same basic paradigm was tested to determine if it could capture quantitatively the branching dynamics of pancreatic morphogenesis.

In particular, our modeling approach was underpinned by two key observations: First, at E18, the ductal network in pancreas appeared surprisingly two-dimensional, with very few "cross-overs", where ducts would stand on top of one another in close proximity, a behavior reminiscent of the mammary gland epithelium. Second, there was huge subtree size heterogeneity within individual ductal trees emanating from the main central ducts (primordial structures derived from reorganization of the central plexus). In particular, some subtrees terminated after just a few branches, while others branch extensively, colonizing large fractions of a given pancreatic lobe (see Figure 4B,C for quantification). In the case of purely ductal end-driven morphogenesis, this suggests that some ductal ends "terminated" early during development, while others continued to branch and expand, with the history of branching decisions encoded in the topology of the ductal network.

A branch generation, n, was defined as the number of branch points separating a given branch from the central ducts from which branching morphogenesis initiates. This generation number could be defined uniquely since, in common with the mammary gland, pancreatic ductal trees form simple graphs, i.e. notably, loops or reconnections at E18.5 between two independent subtrees were not observed starting from the main trunk (although some loops can be found within the central plexus at earlier time-points). The central plexus was defined manually and subtrees were defined as deriving from the first generation of branches that bud from this central plexus. From a physical perspective, it was clear that there must be some termination events occurring; otherwise, the number of ductal termini at generation n would grow exponentially as 2^n. This is not tenable for ducts whose thickness and length are largely independent of the generation number, as observed in both mammary gland and pancreas. Therefore, some ducts must terminate, accounting for the extreme heterogeneity in subtree sizes.

Based on these findings, the further observation that branching morphogenesis takes place in a largely two-dimensional setting, without ductal cross-overs, offers a crucial simplification: Strikingly, when examining small ductal subtrees in pancreas, i.e. ones that had likely terminated early, they appeared to be “trapped” by the spatial constraints imposed by neighboring subtrees. A similar organization is visible in the mammary gland (where secreted TGF-β1 is thought to mediate a feedback signal between ducts and active terminal end-buds), and offers a simple heuristic explanation for how environmental signals can provide a feedback mechanism to arrest ductal bifurcation, viz. ductal termination (Hannezo et al. 2017).

Thus, based on these observations, branching morphogenesis in pancreas is proposed to occur according to three simple local “rules” for the dynamics of active ductal termini that together translate into the formation of the large-scale structure of the organ (see Figure 4E for a sketch):

- An active ductal terminus, A, undergoes a persistent random walk at speed v and persistence time, t_p, and thus randomly explores space is an isotropic (diffusive) manner, leaving behind a trail of “inactive” ducts via cell proliferation. Note that, while the further proliferation of cells in “inactive” ducts may contribute to local dilation and expansion of ducts, the large-scale organization of the ductal network is preserved.
- An active ductal terminus has a probability r_b, presumed constant in time, to branch into two new active ductal termini. One would thus predict, for constant speed v, an exponential distribution of branch size, a feature reproduced well by the experimental data at all length scales above a cut-off at around 20μm (presumably set by the tip/cell size, Figure S6Q).

- An active ductal terminus becomes inactive when it comes into “contact” with another duct, thereby preventing ductal crossovers in the predominantly two-dimensional geometry of the embryonic pancreas. The diffusing signals from maturing ducts may enlarge the contact radius, r_a, to length scales much larger than a cell diameter; but it is still local on the length-scale of the average distance between consecutive branching events.

Note that an important feature of such a model – known as a branching-annihilating random walk – is that it considers all ductal termini to be functionally equivalent, i.e. having an equal probability *a priori* to give rise to a small or large ductal subtree. It is only thought the chance dynamics of the random walk and competition for space with neighboring ductal termini and ducts that subtrees acquire their size heterogeneity. This results in subtree size distributions, expressed in terms of the total number of branches in a given subtree, which are characteristically broad with an exponential tail, fitting qualitatively with the experimental data. Within this framework, most subtrees stochastically stop contributing to branching morphogenesis early in development, while an ever-diminishing surviving population of active ductal termini end up giving rise to nearly all the peripheral regions of the organ.

Comparison between model and experiment

To go beyond such qualitative comparisons, full numerical simulations of the three processes described above were employed. To adapt to the specificity of pancreatic morphogenesis, instead of starting from a single active terminus, as in the study of mammary gland or kidney morphogenesis (Hannezo et al. 2017), an initial condition was taken as a situation in which a central duct is already formed, as in Figure 3H (Figure 4F, left-most panel). In particular, model simulations were performed with 20 active termini located at each side of the central duct, facing outwards, and with a constant spacing L. Note that the ratio, Lr_b/v, defines the only key parameter of the model, comparing the typical branch size to the initial spacing between subtrees. This parameter was calculated experimentally (Figure S6K) and set accordingly in the simulation.

Running the simulation until subtrees reached the average subtree size measured in E18 pancreas networks (Figure 4F, right-most panel), trees were found to self-organize into a pulse of active termini located at the periphery of the growing network, leaving in their wake an internal structure of roughly constant ductal density, as expected from previous work (Hannezo et al. 2017). This organization strongly echoes the results of the EdU kinetics experiments (Figure 4I-N), showing enhanced ductal proliferation at the

periphery of the pancreas during branching morphogenesis. Because of the initial condition chosen, the final configuration of the pancreas was an ellipsoid shape, with large subtree size heterogeneity. To compute with high accuracy the predicted subtree size distribution, averages were made over 500 such simulations. N=20 subtrees were simulated to grow on each side of a central duct. Strikingly, comparing the model prediction with the E18 subtree size distribution data, an excellent agreement (R^2=0.99, S=0.03) was found, arguing that this minimal model is sufficient to recapitulate the statistical heterogeneity of branching patterns and thus, as a consequence, the clone size distributions. Importantly, as Lr_b/v is the key parameter of the model, it was questioned how changes in this parameter would change the predictions of the model. The experimentally measured variance of this parameter (Lr_b/v=1.45+/-0.5, mean +/- 2S.D.) was used to build 95% confidence intervals for the model predictions (see thin lines on Figure 4G), keeping the total simulation time constant for all parameters. The other parameters of the model were set as $r_a r_b/v$=0.25 and a small rotational diffusion (dictating persistence) of π/10. Nevertheless, it should be noted that the exact values of these parameters are largely irrelevant on the resulting dynamics, as it was shown previously via sensitivity analysis that variations of these two parameters had only a very weak effect on the predicted branched structure (Hannezo et al. 2017).

To further demonstrate the robustness of the branching and annihilating random walk model, a number of additional simulations were performed as controls (Figure S6S,T). In particular, a small 3D exploration of space was allowed, to simulate ductal branching within a thin quasi 2D setting, where there is a minimal, but non-vanishing, probability for overlap between ducts. The case was also considered where subtrees where not defined as emerging from the main central duct, but one generation onwards (in green in Figure S6S), to test the effect of a different initial condition (representing for instance a central plexus). Finally, the effect of a constant branch length was simulated (instead of an exponential distribution resulting from stochastic branching at any time-point). Crucially, it was found that the key features of the model, i.e. patterning of the tree via a self-organized propagating wave of peripheral growth, and large-scale heterogeneity of the subtree branching patterns, were conserved in all these variations of the model, suggesting that branching and annihilation were the key features giving rise to the observed phenomenology.

Whether some of the heterogeneity in branch length could be explained by positional effects was also tested. In particular, the distribution of branch lengths in "small" (<30 branches) versus "large" (>60 branches) subtrees (Figure S6R) were analyzed. Interestingly, although there was a slight increase of branch length in large subtrees (Figure S6R), both distributions were well-fit by exponential distributions. We note that this difference in

average size could be due to a slightly larger v (or r_b) for some subtrees; but, alternatively, could be due to subsequent growth/remodeling events within the pancreas. Finally, to probe this question of neutrality further, the correlation in branch lengths of consecutive branches (across all subtrees) was probed and confirmed that it displayed very poor correlation (R^2=0.09 from linear regression, see Figure S6P).

Overall, these results argue that branching morphogenesis in the pancreas and mammary gland, which dominates the large-scale organization of both tissues, is controlled by a similar self-organizing principle, involving sequential rounds of ductal bifurcation checked by ductal termination mediated by local signals from neighboring ducts. Further analogy between mammary gland and pancreatic branching morphogenesis is found at the cellular level. Indeed, the gradual transition of self-renewing multipotent progenitors to fate-restricted ductal- and acinar-committed progenitors during pancreas development mirrors the behavior observed in mammary gland, where bipotent precursors segregate early into fate-restricted self-renewing basal and luminal progenitors, both residing within terminal end-buds and undergoing the same collective branching/termination dynamics.

Theoretical analysis of monoclonal conversion during branching morphogenesis

On this background, the clonal dynamics of self-renewing pancreatic progenitors was then assessed within each individual ductal terminus. Indeed, the above description only deals with an effective description of the collective fate choices made by all progenitors within a given terminus, without making any assumptions or predictions related to the actual number of such progenitors in each terminus. However, the clonal lineage tracing data provides a platform to address this question in further detail. Again, as described previously in the context of the mammary gland epithelium (Scheele et al. 2017), it is supposed that ducts consist of N equipotent self-renewing progenitors, which are randomly allocated to one of the two offspring ducts upon bifurcation, such that each new duct received $N/2$ such self-renewing progenitors. One should note that, relaxing this equal repartition assumption does not give rise to qualitatively different behavior to the one we describe below. Each progenitor then divides once to expand the pool so that there are, again, N progenitors per terminus – the process of renewal. Interestingly, even in the absence of any active competition between progenitors within the terminus, this process would drive monoclonal conversion of lineage-labeled ducts. This is because, upon bifurcation, a clone will be randomly segregated, and thus enriched in one of the termini, and depleted in another. In the limit of randomly allocated cells, i.e. the limit of

strong cell mixing, which is relevant here (as we do not see cohesive streams of clones before monoclonal conversion), monoclonal conversion is expected to occur slowly, as average clone sizes increase only linearly with the number of ductal bifurcations. Therefore, with say $N=30$ progenitors per terminus, monoclonal conversion would almost never be observed over the time-scales relevant for pancreatic morphogenesis (Figure 5C and S6L,M). This is in stark contrast to the experimental data, which is characterized by a seemingly very rapid process of monoclonal conversion of ducts (Figure 5C and S1J-L), on the scale of just a few generations of ductal branching. Therefore, this heavily constrains the number of self-renewing progenitors that are located in each individual ductal terminus. Indeed, simulations of as few as $N=4$ progenitors per ductal terminus yield a rate of monoclonal conversion that is compatible with the experimental data (Figure 5B,C).

One should note that active cell competition during ductal bifurcation would speed up the process of monoclonal conversion, but would not yield qualitatively different results, as average clones sizes would still increase only linearly with generation number. Interestingly, such a behavior of monoclonal conversion of entire ductal subtrees of the pancreas, seen both from ductal-containing clones and acinar-containing clones, validates *a posteriori* such a ductal end-splitting mechanism of morphogenesis.

Lastly, in developing a model of branching morphogenesis, changes that occur during development in the lineage potential of self-renewing progenitors at the ductal termini have not been considered explicitly. From measurements of the clone composition, it is known that termini can play host to multipotent and/or fate-restricted self-renewing progenitors, with the abundance of the former diminishing through development. Clearly, as with the basal and luminal compartments in developing mammary gland epithelium, providing all progenitors at the termini are renewed during the bifurcation process, the branching-annihilating random walk model is agnostic to the potency of the self-renewing population. However, this condition provides a significant constraint on the regulation of the termini supported by purely fate-restricted progenitors – the majority at the later stages of branching morphogenesis. In this case, the niche environment at the ductal termini must be capable regulating independently the multiplicity of both ductal/islet progenitors and acinar-committed progenitors to ensure their renewal during bifurcation, a constraint most easily engineered by having one compartment provide a niche for the other. An interesting extension for further studies would be to refine this model to include explicitly the time-dependent dynamics of multipotency, i.e. how the fate transitions between different cell types occur and are regulated spatio-temporally during branching morphogenesis.
